# Strategy of Transcription Regulation in the Budding Yeast

**DOI:** 10.1371/journal.pone.0000250

**Published:** 2007-02-28

**Authors:** Sagi Levy, Jan Ihmels, Miri Carmi, Adina Weinberger, Gilgi Friedlander, Naama Barkai

**Affiliations:** 1 Department of Molecular Genetics and Department of Physics of Complex Systems, Weizmann Institute of Science, Rehovot, Israel; 2 Howard Hughes Medical Institute, Department of Cellular and Molecular Pharmacology, University of California, San Francisco, California, United States of America; University of Glasgow, United Kingdom

## Abstract

Cells must adjust their gene expression in order to compete in a constantly changing environment. Two alternative strategies could in principle ensure optimal coordination of gene expression with physiological requirements. First, characters of the internal physiological state, such as growth rate, metabolite levels, or energy availability, could be feedback to tune gene expression. Second, internal needs could be inferred from the external environment, using evolutionary-tuned signaling pathways. Coordination of ribosomal biogenesis with the requirement for protein synthesis is of particular importance, since cells devote a large fraction of their biosynthetic capacity for ribosomal biogenesis. To define the relative contribution of internal vs. external sensing to the regulation of ribosomal biogenesis gene expression in yeast, we subjected *S. cerevisiae* cells to conditions which decoupled the actual vs. environmentally-expected growth rate. Gene expression followed the environmental signal according to the expected, but not the actual, growth rate. Simultaneous monitoring of gene expression and growth rate in continuous cultures further confirmed that ribosome biogenesis genes responded rapidly to changes in the environments but were oblivious to longer-term changes in growth rate. Our results suggest that the capacity to anticipate and prepare for environmentally-mediated changes in cell growth presented a major selection force during yeast evolution.

## Introduction

Cellular functionality is tightly coupled to the external environment. The type of nutrients available defines the internal metabolic flow, while their abundance often limits the rate of biomass production and energy available for growth. An abundance of toxins impede upon various aspects of cellular machinery, including metabolic capacity, protein stability or DNA integrity. Over evolutionary time scales, cells may encounter virtually endless environmental states at widely different frequencies. Maintaining optimal functionality in the presence of such external variability is a central evolutionary constraint.

Gene expression plays a central role in the adaptation to changing conditions. Studies in the budding yeast *S. cerevisiae*, for example, have shown that cellular transcription program is dramatically modified by changes in nutrient availability, growth conditions, temperature, and a variety of other environmental condition tested.

How do cells coordinate their gene expression with varying environments? One strategy is to use feedback mechanisms, which directly link gene expression with internal needs. Within such strategy, internal variables, such as the rate of biomass production or the internal pools of nutrients or energy, feedbacks to properly tune gene expression with the corresponding functional needs. Cells then respond directly to the relevant functional parameter that needs to be monitored, regardless of the specific external perturbation that may have altered this parameter. The primary advantage of such internal feedback strategy is the capacity to ensure optimality of response under a wide diversity of external conditions. It may limit, however, the speed of the response since environmental changes will alter internal characters (*e.g.* growth rate) only after a delay (*e.g.* when the intracellular pool of nutrient is diminished).

An alternative strategy for coordinating gene expression with physiological requirements is to infer the expected physiological state from the external environment. This can be done using evolutionary-tuned signaling pathways. For example, in yeast, the TOR and the PKA pathways sense the source and availability of carbon and nitrogen and regulate the expression levels of multiple gene groups [1]–[3]. In principle, the information received by those signaling pathways could coordinate gene expression with the expected growth rate at the specific level of nutrients available. Such a strategy can be used to optimize the speed of the response. It is likely, however, to limit the range of environments that can be properly recognized. In particular, it will hinder optimal response to newly encountered situations.

The need to coordinate gene expression with internal state is best exemplified in the case of ribosomal biogenesis. In rapidly growing yeast cells, the synthesis of ribosomes accounts for the cell's single largest expenditure of biosynthesis energy. With the need to produce ∼2000 ribosomes every minute, 60% of total transcription is devoted to ribosomal RNA. Similarly, 50% of RNA polymerase II transcription, as well as 90% of mRNA splicing, are devoted to ribosomal protein [4]. Over 150 non-ribosomal genes are involved in various aspects of ribosomal RNA processing or transport [5]. Tight coordination of ribosomal biosynthesis with the requirement for protein translation is thus needed to maintain efficient utilization of biosynthesis resources. Indeed, the number of ribosomes in yeast cells appears to be linked to cell size and growth rate [6], [7]. Similarly, tumor cells often express high levels of ribosome and other translation related factors, in accordance with their elevated growth rates [8], [9].

In bacteria, the rate of ribosome synthesis increases approximately with the square of the growth rate [10]. Underlying this growth-dependent control are well-studied examples of regulation by an internal-feedback. The purine nucleotide (ATP and GTP), whose concentration reflect the nutritional state as well as the translational activity of the cell, play a key role in this regulation. In fact, purine NTP levels directly regulates rRNA transcription [11]–[14], and thus determine the rate of ribosomal biogenesis. An additional layer of regulation by internal feedback is provided by the stringent response, which is induced by uncharged tRNA. Uncharged tRNAs accumulate when the internal level of amino-acids is not sufficiently to support the rate of protein synthesis. In those instances, the stringent response feedbacks to repress the transcription of genes associated with the translational apparatus, including tRNAs, rRNAs, ribosomal proteins, translational factors, and synthetases [15]–[17].

Growth-rate dependent control of gene expression was implied also in the regulation of gene expression in the budding yeast [18]. The dependence of gene expression on growth rate was characterized most comprehensively using continuous chemostat cultures growing at steady state [18]. A large multitude of genes exhibited an expression pattern that was strongly correlated with growth rate. In particular, ribosomal biogenesis gene expression increased with increasing growth rate, whereas the expression of stress-related genes decreased. Based on the genomic distribution of growth-correlated genes, it was suggested that growth-rate dependence is achieved through replication-mediated changes in chromatin modification [18]. In the context of the internal vs. external regulatory strategies discussed above, however, the interpretation of the observed correlations is difficult, since changes in growth rate necessarily involved change in the amount of the limiting factor (glucose) in the medium. Gene expression was measured at steady state, and, at least formally, it may be that expression levels were tuned by the external glucose concentration, according to the evolutionary-expected growth rate, rather than as feedback by the growth-rate itself.

More generally, since under most conditions the actual and environmentally-expected states are compatible, it is difficult to discern whether gene expression is regulated predominantly by an internal feedback-mechanism, or whether it is tuned by an environmental signal based on its expected influence on the internal state. To try and overcome this limitation, we undertook two approaches. First, we set to measure gene expression under conditions that decouple the perceived environmental signal from its actual effect on cell growth. This was done by focusing on a specific mutant (*adh1*) which grows better on glycerol than on glucose. Under such conditions, environmental cues dominate, leading to increased level of ribosomal biogenesis gene expression in slowly-growing cells. Second, we examined the correlation between gene expression and growth rate during the dynamic response of chemostat grown culture to environmental changes. The focus on the dynamics, rather than the steady state behavior, allowed us to examine the relative timing of the two responses. Ribosome biogenesis gene expression responded rapidly to changes in the environments but was rather oblivious to longer-term changes in growth rate. Lack of correlation between growth rate and ribosomal biogenesis gene expression was also observed in a compendium of 196 deletion mutants, for which both growth rates and genome-wide expression profiles were reported [19]. Taken together, our results suggest that the apparent coupling of translation-related gene expression and growth rate is not causal, but reflects the evolutionary fine-tuning of signal transduction mechanisms. The capacity to recognize and prepare to conditions that may alter changes in growth rate was probably a major selection force during yeast evolution.

## Results

### Ribosomal biogenesis gene expression is highly responsive to changing environments

We analyzed the expression levels of genes involved in ribosomal biogenesis using an annotated database of over 1500 genome-wide expression profiles in *S. cerevisiae* [20]. Our previous studies had identified two groups of co-regulated genes involved in making the ribosome [20]–[22]. The first group includes genes that code for the ribosomal proteins (RP) themselves. The second group is composed of genes that assist in the proper assembly of the ribosome, such as genes involved in the processing of rRNA, and is denoted here as ribosomal biogenesis gene module.

**Figure 1:**
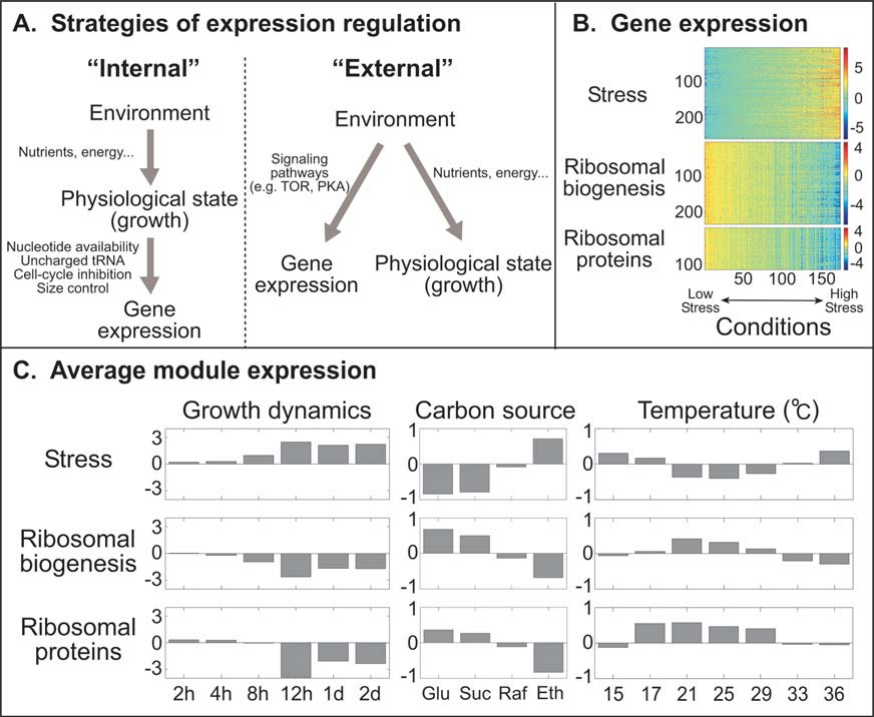
**(a)** Environmental versus growth dependent modulation of gene expression. Gene expression can be modulated by direct signaling pathways which sense the environmental cues. Alternatively, it can be regulated by internal cues which are coupled directly to cell growth. In this study we characterize the relative contribution of external vs. internal sensing to the regulation of gene expression. (b,c) Expression profile of ESR modules during growth at different environmental conditions (reported in [25]; see table S1 for the list of genes). (b) The colormap represents the log_2_-expression-ratio of the respective gene module. The conditions are sorted by the average expression level of the stress module. (c) Average log_2_-expression-ratio of the respective gene module under the indicated condition. Stress-induced genes are activated, while ribosomal biogenesis and RP genes are repressed under conditions associated with slow growth (e.g. non-optimal temperatures, non-fermentable carbon source, or stationary phase).

As described previously, genes of both groups are co-expressed under a large number of conditions, and display a strong inverse correlation with genes induced as part of the general stress response [23]. This correlation pattern was particularly evident in the prototypic responses, termed environmental stress response (ESR), to a variety of environmental stresses (including heat-shock, peroxide shock or high osmolarity) [24], [25]. The ESR is characterized by the repression of ribosomal proteins and ribosomal biogenesis genes together with the induction of a common set of stress-related genes (Fig 1b). We note that although both RP and ribosomal biogenesis genes were repressed by environmental stresses, their responses were characterized by different kinetics (rapid repression of ribosomal biogenesis genes followed by a slower repression of RP genes) leading to their identification as two separate transcription modules (i.e. sets of coordinately expressed genes) [25].

Gene expression in unperturbed cells was also reported [25] (Fig. 1c). The expression of both RP and ribosomal biogenesis genes was maximal during logarithmic growth, and decreased as the cultures approached saturation. As expected, the level of ribosomal biogenesis gene expression was also correlated with the efficiency by which the corresponding carbon is utilized: expression was high in cells grown in a fermentable carbon source (glucose), whereas low expression was observed during growth in non-fermentable carbon sources such as ethanol. Similar differences in the level of ribosomal biogenesis genes were observed also when comparing steady state growth at different temperatures. In all the above conditions, the stress-induced part of the ESR exhibited high expression levels in conditions where ribosomal biogenesis genes expression was low, and low expression levels in conditions where ribosomal biogenesis gene expression was high. Although growth rates were not reported, the gene expression pattern seems consistent with the general notion that the expression of ribosomal biogenesis gene increases with growth rate, whereas the expression of stress genes decreases.

### Gene expression in the presence of internal vs. external conflict

**Figure 2:**
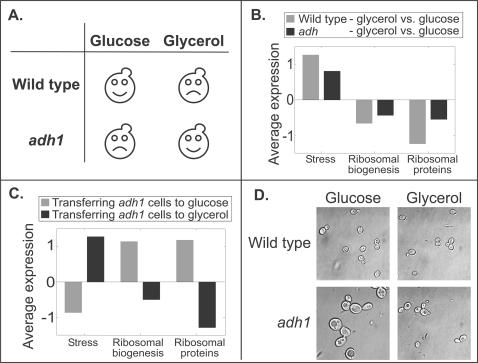
Ribosomal gene expression under “confusing” conditions. (a) Glucose is the preferred carbon source for wild-type yeast cells. In contrast, *adh1* mutant cells grow faster when glycerol is available (See also Fig S1). (b) Wild-type and mutant cells were grown to log phase on media containing glucose or glycerol as the only carbon source (Methods). The expression profile on glycerol vs. glucose was quantified with microarrays. The bars represent the average log_2_-ratio-expression of the ESR modules. Notably, both wild type and mutant cells activate the ESR program. Stress genes are induced while ribosomal biogenesis genes and RP are repressed. (c) Response of *adh1* mutants to change in carbon source. Overnight cultures of *adh1* cells were transferred both to glycerol and to fresh glucose media. The bars represent the average expression after 30 minutes with respect to the initial cell culture expression (log_2_-ratios). As expected, the ESR is repressed upon transfer to fresh glucose medium. However, upon transfer to glycerol, the favorable carbon source of these mutant cells, the ESR is activated. Specifically, stress genes are induced while ribosomal biogenesis and RP genes are repressed. (d) Wild-type and *adh1* mutant cells were monitored upon transfer to glycerol versus their transfer to fresh glucose. As previously reported, the size of slow growing wild type cells (glycerol) is smaller than fast growing ones (glucose) [26]. Interestingly, *adh1* mutants display an opposite behavior. Specifically, slow growing *adh1* mutants (glucose) are appreciably larger than the fast growing ones (glycerol). Thus, it appears that the cell size may also be tuned to the environmental signals rather than to cell division.

To examine whether the apparent correlation between the level of ribosomal biogenesis gene expression and growth rate reflects a feedback-mediated regulation that is sensitive to the growth rate itself, we sought a situation where the recognized environmental signal as perceived by the cell is separated from its actual effect on growth. Wild-type yeast cells grow most rapidly when provided with glucose as a carbon source. We considered a mutant strain deleted of the gene *ADH1*, which codes for an enzyme responsible for ethanol production during glucose fermentation. This strain cannot ferment glucose, and consequently grows faster on non-fermentable carbon sources (*e.g.* glycerol) than on glucose (Fig. 2a, S1).

We reasoned that comparing the gene expression pattern of *ADH1* cells grown in glucose vs. glycerol would allow us to distinguish between internal vs. external strategies of gene expression regulation. If internal, growth-rate dependent feedback dominates, ribosomal biogenesis gene expression will be higher in glycerol than in glucose, in accordance with the higher growth rate of the cells. Importantly, this prediction holds regardless of the mechanism which hinders *ADH1* growth in glucose. Alternatively, if signaling by the external environment dominates, ribosomal biogenesis gene expression will be higher in glucose, in accordance with the evolutionary expectation of higher growth rate of wild-type cells in glucose.

Using full-genome microarrays, we compared the expression profile of log-phase *adh1* mutant cells grown on glycerol vs. glucose medium (Fig. 2b). The expression of ribosomal biogenesis and RP genes was higher in glucose, although the growth rate of the cells was lower. Transfer of cells from glucose to glycerol led to a corresponding repression of ribosomal biogenesis genes (Fig. 2c). Similarly, the expression of stress genes was higher in glycerol (Fig. 2b), and was induced upon transfer from glucose to glycerol (Fig. 2c). Taken together, it appears that the expression of ribosomal biogenesis genes, as well as that of stress genes, was tuned to the environment rather than to the rate of cellular growth.

Interestingly, cell size was also tuned to the environmental signal rather then the growth rate. In *S. cerevisiae*, poor media and low growth rates correspond to smaller cell size. We observed, however, that *adh1* cells growing in glucose were larger than *adh1* cells growing in glycerol (Fig. 2d). Moreover, *adh1* cells growing on glucose were significantly larger than wild-type cells in glucose. This abnormal size may reflect the imbalance between their (high) ribosomal content and their (slow) growth rate, in consistence with the observed impact of ribosomal content on cell size [26].

### Temporal adaptation to perturbation in continuous cultures

Our results suggest that upon conflicting signals, when the expected growth rate at a particular environment is different from the actual rate of growth, the environmental signal dominates. Next we asked whether growth-dependent signals play an important role under non-conflicting situations. As we argue above, however, in the absence of conflict the internal physiological state (*e.g.* growth rate) is compatible with the environmentally-expected one, making it is difficult to disentangle the signals received from the external environment from these that are generated as feedback from internal processes. For examples, changing the steady state growth rate in continuous cultures necessarily involves a change in the amount of limiting factor. It is thus hard to discern whether the changes in gene expression result from the change in growth rate itself, or whether they were generated as a response to the change in the abundance of the limiting factor.

We reasoned that characterizing temporal kinetics, rather than steady state behavior, may overcome this limitation. Consider an environmental perturbation that modulates both the gene expression and the growth rate. At steady state, these changes are likely to be coordinated. By following the temporal evolution of both responses, however, we can conclude whether the change in growth rate followed or precede the changes in gene expression. For example, if internal feedback mechanism is at function, any change in growth rate will be followed by a change in gene expression.

**Figure 3:**
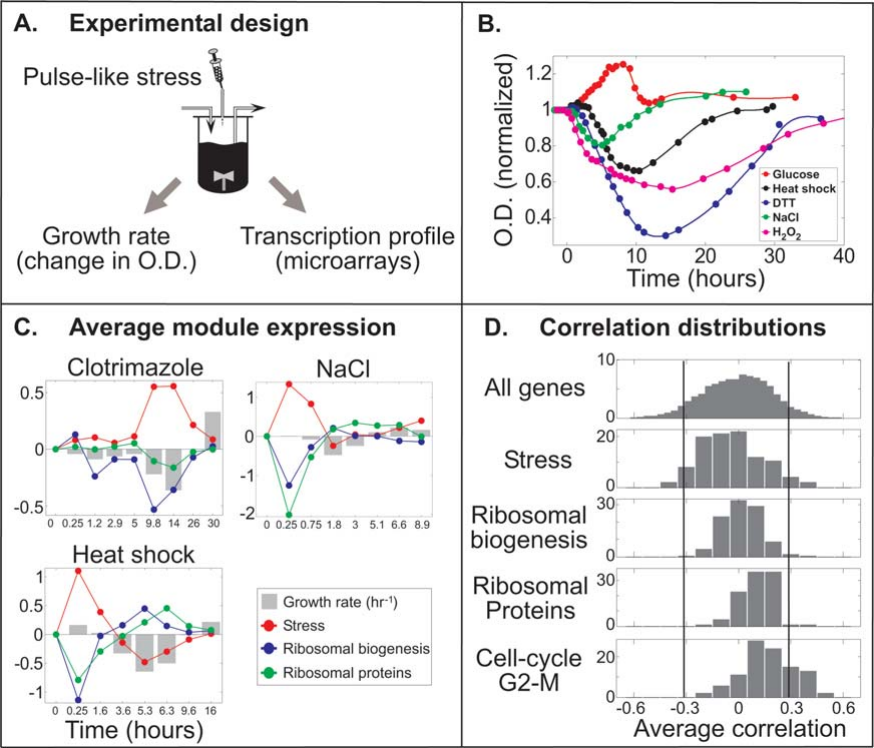
Gene expression versus growth rates in continuous cultures. (a) Steady state growing cells were subjected to different environmental stresses. Growth rate was calculated from the measured optical density. Simultaneously, the profile of gene expression was quantified with microarrays. In our study we looked for possible correlations between growth and gene expression (see Methods for more details). (b) Cells grown at steady state in a glucose-limited chemostat were subjected to 5 different environmental cues. We examined 4 stress pulses: H_2_O_2_ (0.6mM-magenta curve), DTT (6mM-blue curve), NaCl (0.4M-green curve), heat shock (39°C for 5 hours-black curve), and a stress-relieving pulse of glucose (0.2 g/l-red curve). The Y-axis is normalized such that the steady state O.D. (at t<0) is 1. The markers designate O.D. sampling time, and solid lines are spline interpolations of the data. The perturbations in histidine-limited chemostat are shown in figure S2. (c) Dynamics of the average log_2_-ratio-expression for three ESR gene modules. The DTT, NaCl and heat shock pulses were given during glucose depletion, and the clotrimazole perturbation was given in histidine limitation. Bars designate the cells growth rate, which is normalized relative to the steady state growth rate (see Fig. S3 for other perturbations dynamics). (d) Each gene was assigned a growth correlation score (GCR), quantifying the correlation between its expression level and growth rate, averaged over all perturbations (Methods). The GCR distribution of several gene modules is shown. The X-axis represents the calculated average correlation, and Y-axis represents the fraction of genes within the module. The two vertical lines designate the threshold for the 5% of genes with the highest or lowest GCR. Notably, the distribution for the ribosomal biogenesis genes lies between the thresholds, while the cell-cycle G2-M gene group is enriched with positive GCR genes.

To define a well-controlled environment where the kinetics of both gene expression and growth rate can be monitored, we grew cells in continuous cultures in a chemostat (Methods). Cells were grown to steady state, and were subsequently subjected to perturbations. Microarrays were used to follow their genome-wide transcription response to each perturbation at subsequent time points following the perturbation. Notably, the changes in gene expression changes are analyzed relative to the gene expression in the unperturbed (steady state) culture, and thus reflect only the response to environmental cues. In parallel, we measured also the cells growth rate, by following the changes in their optical density (Fig. 3a, 3b, S2). At steady state, the optical density remains constant in time, and the rate of cell growth is set by the dilution rate of the chemostat (0.2 hr^−1^ in our experiment) [27]. Since the cells are continuously diluted, and dilution rate remains fixed throughout the experiment, any change in growth rate will be reflected practically immediate by a change in the optical density.

Since changes in growth rate necessarily involve changes in other factors, such as abundance or level of nutrients or drugs in the media, it was important to diversify the type of perturbations, so that a general correlation with growth-rate could be inferred. We considered four environmental stresses (Heat shock, high osmolarity, peroxide, DTT), a drug (clotrimazole), and supplementation of the limiting factor (histidine or glucose, respectively) (Table 1). Moreover, the experiments were performed using two chemostat cultures, growing in different limiting nutrients (histidine and glucose). Taken together, we analyzed 10 time course experiments, with a total of 83 arrays.

**Table 1:** Environmental cues imposed on steady state growing yeast cells.

Limiting factor	signal	Final conc./Temperature in the chemostat	Notes
Histidine	Clotrimazole	10 µM	Sigma C6019. Dissolved in DMSO
	DTT	6 mM	Sigma D9779.
	NaCl	0.31M	-----
	Heat shock	Shift from 30°C to 37°C for 5 hours	-----
	Histidine	+2 mg/l	Double the limiting factor level
Glucose	H_2_O_2_	0.6 mM	-----
	DTT	6 mM	Sigma D9779.
	NaCl	0.4M	-----
	Heat shock	Shift from 30°C to 39°C for 5 hours	
	Glucose	+0.2 g/l	Double the limiting factor level

All perturbations led to a significant change in culture density, ranging from 15% increase in biomass (addition of histidine to the histidine-limited chemostat) to 70% reduction in biomass (addition of DTT to the glucose-limited chemostat) (Fig. 3c, S3). Significant changes in cell growth were observed about ∼1 hour after the onset of the perturbation. The rate of growth kept changing for long time periods, adapting back on a scale of 10–30 hours, depending on the experiment. Typically, an ‘over-shoot’ was observed before reaching steady state, where cells grew faster than the dilution rate (Fig. 3c, S3).

### Temporal kinetics of ribosomal biogenesis gene expression vs. growth rate

Changes in gene expression were observed immediately following the perturbation (Figs. 3c, S3). The gene expression response was similar to that characterized in batch cultures: environmental stresses led to a strong induction of stress-induced genes and the repression of ribosomal biogenesis genes. Also as expected, the addition of glucose to a glucose-limited chemostat led to a decrease in the ESR genes, and increase in RP and ribosomal biogenesis genes (Fig. S3).

Maximal change in gene expression was typically observed already at the first time point examined (10–20 minutes after the onset of the perturbation). In most perturbations, growth rate was hardly altered at that time. Thus, the initial gene expression response appears to be dominated by direct environmental signals, and not by growth-rate dependent feedbacks.

The strong initial response to environmental changes may mask further growth-dependent signal. We asked whether growth-rate effects may dominate during the recovery from perturbation. The environmental perturbations were given in a pulse-like addition to the media, and were washed out of the media on a time scale of ∼5 hours (corresponding to the dilution rate). The long-time temporal recovery from perturbation varied between experiments, but no consistent correlation between growth rate and ribosomal biogenesis gene expression was observed. During the recovery from heat-shock, for example, slow growth was associated with high levels of ribosomal biogenesis genes (Fig. 3c). During the recovery from DTT, temporal changes in growth rate seem to follow gene expression, rather then vice-versa: gene expression adapted to near its steady state level at 8.6 hours, a time where growth rate was minimal (Fig. 3c). Similarly, steady state-levels of gene expression were observed at 27 hours, a time when growth rate was still much higher than its steady state value. In a large number of perturbations, such as NaCl, gene expression was relatively constant during the long-term adaptation, although significant changes in growth rate were observed (Fig. 3c). Some correlation between gene expression and growth rate was observed during the response to clotrimazole (Fig. 3c). However, even in this case, the large increase in growth rate (over-shoot) during the final adaptation back to steady state (at 31 hours) was not accompanied by a corresponding change in gene expression.

To explore the link between ESR gene expression and growth rate more systematically, we measured the correlation between the average expression of each gene group and growth rate (Table S2). This was done separately for each perturbation and then averaged over all perturbations tested. As expected from the analysis of individual perturbations, no significant correlation was observed for either of the groups considered (RP, ribosomal biogenesis genes and stress genes) (Table S2).

Taken together, our results indicate that environmental signal dominate over possible growth-rate dependent feedback in the modulation of gene expression during response to perturbation.

### Ribosomal biogenesis gene expression vs. growth rates in mutant cells

An additional means to decouple the growth rate from the environmental signal is by genetic mutations. We have asked whether growth-rate dependent feedback on gene expression can be recognized under such conditions. Gene deletion may impact gene expression through a multitude of effects, depending on its precise function. However, if a direct growth-rate dependent feedback significantly contributes to the regulation of gene expression, it would lead to an apparent correlation between expression levels and growth rates when a large number of unrelated mutants are examined.

We considered the previously published compendium of deletion profiles, characterizing the transcription profiles of 270 deletion mutants [19]. Growth rates of 196 of these deletion strains were measured in an independent experiment, allowing us to examine the correlation of gene expression with cell growth rate. Consistent with our results above, no correlation was found between the expression levels of the ribosomal biogenesis genes and the reported growth rates (r = 0.03). A small inverse correlation was observed with the expression of the stress genes (r = −0.19), but this correlation was only marginally significant and considerably lower than those observed with the highest correlated gene groups (e.g. cell cycle group with r = 0.58, see also Table S2).

Interestingly, a strong, and highly significant correlation was observed between the RP gene expression and the growth rate (r = 0.53). This is in contrast to the negligible correlation observed in the chemostat experiment. The decoupling of RP gene expression from ribosomal biogenesis gene expression is also unique to this dataset, as in most other datasets expression of these groups is tightly correlated. A possible explanation is that the mutant strains have undergone further genetic adaptation (secondary mutations *c.f.* Ref [28]) that adjusted the level of ribosomal proteins with growth rates [29]. Further experiments are required to examine this possibility.

### Identifying growth-correlated genes

**Figure 4:**
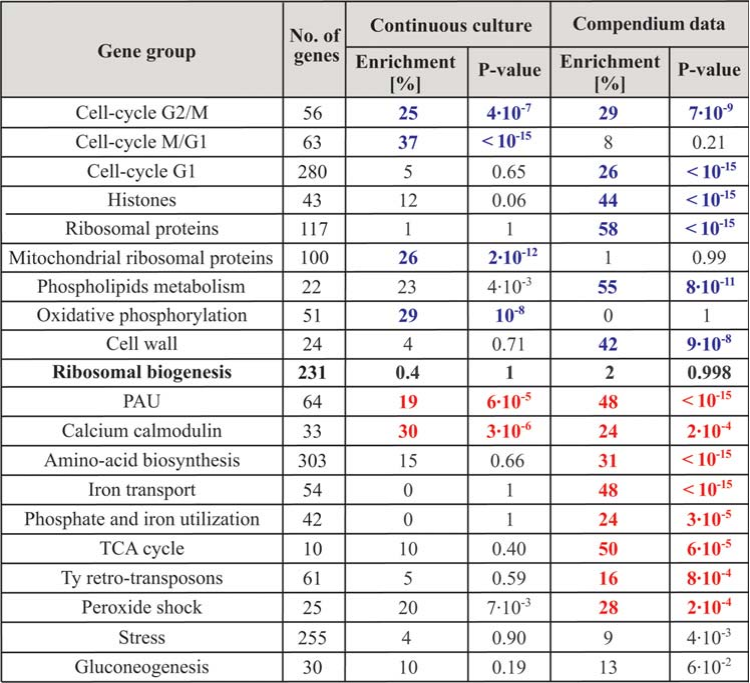
Enrichment of positive and negative correlated genes with previously defined gene modules. The table summarizes the analysis which spanned over hundreds of gene modules. For each gene group and experiment we calculated the enrichment with the positive (blue) and negative (red) correlated genes. Note that none of the gene modules were enriched with both positive and negative correlated genes and only the highest score is displayed. Less significant enrichments are shown in black (P-value>10^−3^) (see also Tables S3).

Taken together, it appears that growth rate *per-se* has a minimal impact on the expression levels of genes involved in ribosomal biogenesis. To examine whether other gene groups display a tighter link to growth rate, we extended the analysis described above to all yeast genes. Specifically, we assigned each gene a growth correlation score (GCR), quantifying the correlation between its expression level and growth rate, averaged over all perturbations (Methods). A positive GCR indicates a correlation with growth rate, whereas a negative GCR indicates an inverse correlation. We focused on the 5% of genes with the highest or lowest GCR, respectively and characterized their properties by analyzing enrichment within different co-expressed gene groups defined previously [20], [22] (Fig. 3d). To examine the generality of the results, we repeated the same analysis also for the compendium of deletion mutants, using the available gene expression and growth rate of the strains, as described above. The results are summarized in Figure 4.

The group of genes that are positively correlated with growth was enriched primarily with cell cycle genes. In the chemostat experiment, enrichment was found primarily for genes regulated during the G2/M and M/G1 transition (p-value of 4?10^−7^ and <10^−15^, respectively), whereas in the compendium data enrichment was found for the G2/M transition as well as G1/S transition (p-values of 7?10^−9^ and <10^−15^, respectively). Other groups that were linked to growth included Mitochondrial ribosomal proteins and oxidative phosphorylation (chemostat experiment), and also gene groups related to histones, ribosomal proteins, phospholipids metabolism and cell wall (deletion mutants). In both experiments, the group of genes that were negatively correlated with growth was enriched with calcium calmodulin related genes (Figure 4).

## Discussion

### Summary

Gene expression is responsive to both external and internal cues. In this study, we wished to characterize the relative importance of these two modes of signaling to the regulation of genes involved in ribosomal biogenesis. Specifically, we sought to characterize the potential importance of internal feedback mechanisms that sense and respond to the cellular physiological state. Such growth-dependent regulation is well characterized in bacteria leading to direct dependency of the rate of ribosomal biogenesis on cellular growth rate. This feedback is mediated, at least in part, by the nucleotide dependency of rRNA transcription, and by the accumulation of uncharged tRNA, through the stringent response [10], [13], [15], [30].

Our results indicate that in yeast, growth-related signals do not contribute significantly to the expression of ribosomal biogenesis genes. Several observations support this conclusion. First, under “confusion” situation, where the environmental signal and actual growth rate conflict, gene expression appears to be linked to the environment and not to the growth rate. Second, even in the absence of conflicting signals, the linkage of ribosomal biogenesis gene expression to growth rate relies on the availability of a proper environmental signal. Thus, no correlation was observed during the long-term recovery of chemostat-grown cells, when large variations in growth rate were observed, and we did not detect situations where ribosomal biogenesis gene expression was clearly dictated by growth rate. Similarly, no linkage between these factors was observed in a compendium of deletion mutants, where both gene expression and growth rate were modified not by changing an environment but rather by deleting specific genes.

We identified several gene groups which displayed tight correlation to growth rate. With the exception of the cell cycle genes, most groups identified were specific to a particular experimental condition (see also Table S3). It may be that correlation with growth is observed only for genes involved in rate limiting processes; For example, gluconeogenesis genes displayed a correlation with growth in the glucose-limited chemostat (Table S3). The correlation of amino-acid biosynthesis and phosphate utilization groups with growth rate in the compendium of deletion mutant may reflect limitation of the media used.

In conclusion, it appears that yeast cells control the level of ribosome biogenesis gene expression primarily by responding to environmental signals. The apparent correlation between gene expression and growth rate, observed under steady state conditions, is likely to reflect the evolutionary fine-tuning of these signals with the expected growth rate, rather than direct growth-dependent signals. This strategy might compensate the exact tuning of gene expression with growth rate, particularly under conditions that are rarely encountered over evolutionary times. On the other hand, it allows for rapid response and preparation to anticipated changes that will impact the growth rate. Our results suggest that the capacity to anticipate and prepare for environmentally-mediated changes in growth rate, before such changes actually occur, presented a major selection force during yeast evolution.

## Methods

### Media and strains

For the limiting histidine and glucose chemostats we used the following two strains, respectively: W303-1 bar1::P_ADH1_-CFP-*KANMX* (*MAT*a, *leu2-Δ1, trp1-Δ63, his3-Δ200, ura3-52, ade2-101*) and BY4741 (Euroscarf; *MAT*a, *his3-Δ1, leu2-Δ0, met15-Δ0, ura3-Δ0*). For the mutant experiments we used BY4741 *adh1*::*KANMX* cells (Euroscarf), and wild-type BY4741 cells were used as a control.

For the *adh1* mutant experiment, YPD and YP-glycerol (2%) media were used. Histidine limited chemostat medium consisted of bacto-yeast nitrogen base without amino acids and with ammonium sulfate (0.67%), glucose (2%) and a drop-out mix (0.2%) of the following combination: L-Histidine (1.3 mg), Adenine sulfate (20 mg), Uracil (20 mg), L-Tryptophan (20 mg), L-Leucine (100 mg), L-Arginine (20 mg), L-Methionine (20 mg), L-Tyrosine (30 mg), L-Isoleucine (30 mg), L-Lysine (30 mg), L-Phenylalanine (50 mg), L-Glutamic acid (100 mg), L-Aspartic acid (100 mg), L-Valine (150 mg), L-Threonine (200 mg), L-Serine (400 mg). The final concentration of histidine in this medium is 2 mg/l. Limited glucose medium consisted of bacto-yeast nitrogen base without amino acids and with ammonium sulfate (0.67%), glucose (0.02%) and a drop-out mix (0.2%) as mentioned above but with 20 mg/l of L-histidine. Media for chemostat experiments included also 20 µl/liter of antifoam (Sigma A5758). Importantly, media composition was chosen such that each chemostat has a single limiting factor. Specifically, the response of the yeast culture to addition of different medium components was tested to assure that the cells are sensitive only to changes in histidine or glucose concentrations.

### adh1 mutant experiment

*adh1* deletion strain and its isogenic wild type strain BY4741 were used to generate genome wide expression profiles. For the first experiment (Fig. 2B), cultures were grown in YPD for 8 hours and then washed and diluted into either YPD or YP-Glycerol. The cells were harvested after 4-5 duplications, upon reaching the concentration of ∼2*10^6^ cells/ml (see also Fig. S1). For the second experiment (Fig. 2C), *adh1* strain was grown on YPD to O.D. 0.4, and then washed and diluted into either YPD or YP-Glycerol. Cells were harvested after 30 minutes of growth and compared to samples of the initial overnight culture (at time t = 0).

### Continuous culture experiments

Continuous cultures were grown in BioFlo 110 fermentors (New Brunswick Scientific) in the following conditions: dilution rate = 0.2 h^−1^, fermentor working volume = 750ml, temperature = 30°C, agitation speed = 500 RPM, air flux = 1 LPM, and the dissolved oxygen level was always above 70% of the saturation level. The pH of the media was titrated with NaOH to values of 5 and 5.3–5.6 for the histidine and glucose limited chemostats, respectively. After reaching steady state, cells were subjected to 10 different environmental cues (Table 1). Cell samples were harvested at various times during each perturbation. As a control, batch culture cells were grown in similar media and harvested at late logarithmic phase (Note that patterns of gene expression in steady-state closely resemble those of corresponding batch cultures just before they exhaust the nutrient [31]).

### Microarray hybridization and data quantification

Samples were collected at various time points and RNA was isolated in order to generate cDNA, which was then labeled by the indirect amino-allyl method. Cy5-and Cy3-labeled cDNA was hybridized onto microarrays of ORFs representing the complete *S. cerevisiae* genome (University Health Network, Ontario), using cDNA from untreated cultures as reference. The arrays were scanned, and expression data was extracted by image analysis. Expression ratios were log_2_ transformed, corrected for technical biases and normalized (see also Text S1).

### Module identification

Transcription modules were identified by applying the Iterative Signature Algorithm to the full expression dataset comprising all conditions [20], [21]. The Iterative Signature Algorithm is an iterative extension of the Signature Algorithm, designed for a global decomposition of large-scale expression matrices into transcription modules, in the absence of a-priory information. Modules are fixed-points of the Signature Algorithm, and are identified in a heuristic search by iterating from a large ensemble of randomly composed input sets until convergence is reached. The gene threshold of the Signature Algorithm determines the resolution of the analysis. The full set of transcription modules used in this work is available in the Supplementary Materials and further details are presented at the following URL: http://barkai-serv.weizmann.ac.il/Modules.

### Correlation between growth rate and gene expression

The measured O.D. was interpolated using the Matlab csaps function (Fig. 3b, S2). The cells growth rate (µ) was calculated using the chemostat cell density equation: 
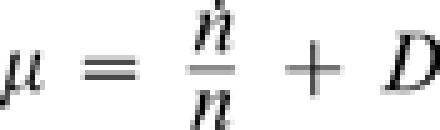
, where n is the cell density (interpolated O.D) and D is the dilution rate [27]. Correlations between growth and gene expression were calculated using the Matlab corrcoef function. Each gene was assigned a growth correlation score (GCR), quantifying the correlation between its expression level and growth rate, averaged over all perturbations. Sorting the genes by their GCR revealed two gene groups: the group of 5% most positive correlated genes (positive GCR) and 5% most negatively correlated genes (negative GCR). In order to reduce noise, we neglected genes that were not affected by the environmental perturbations (see Text S1).

Our study focused on finding the enrichment of positive GCR and negative GCR genes in different co-regulated gene groups, which were previously defined by the signature algorithm [20], [21]. The analysis was unbiased and done over all gene modules within all threshold levels. The P-value for each group enrichment with positive and negative GCR was obtained by calculating the chance of getting at least such an enrichment by chance. Specifically: 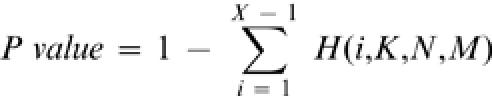, where 
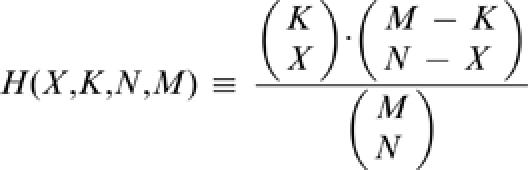
 is the hyper-geometrical distribution designating the probability for enrichment of X of a possible K correlated genes from a group with N genes (module size) without replacement from a pool of M genes.

Gene groups were sorted both by their P-value for enrichment with positive GCR, and by their P-value for enrichment with negative GCR. The analysis was done for the continuous culture experiments considering all 10 perturbations (Figure 4). The analysis was done also for each of the two limiting factor experiments separately (only the 5 perturbations in histidine or glucose depletion, Table S3). The same calculation was repeated for the compendium data. Additional figures and tables are available in the Supplementary Material.

### Accession numbers

All raw data is available through Gene Expression Omnibus (GEO) accession number GSE6302, at the following website: http://www.ncbi.nlm.nih.gov/geo.

## Supporting Information

Figure S1 Growth curves of adh1 and wild type cells
(0.42 MB PDF)

Figure S2 Response of cells grown in steady state to environmental perturbations
(0.46 MB PDF)

Figure S3 Dynamics of average module expression and cell growth upon environmental perturbations. The average log2-ratio-expression is presented for three ESR gene modules. Bars designate the measured cells growth rate, which is normalized relative to the steady state growth rate.
(0.44 MB PDF)

Table S1 List of gene modules
(0.18 MB PDF)

Table S2 Correlations between growth and the modules average expression
(0.07 MB PDF)

Table S3 Effect of medium limitations on the enrichment of positive and negative correlated genes with gene modules.
(0.09 MB PDF)

Text S1 Supplementary Methods
(0.02 MB PDF)
