# Supplementary material for: Strategy of Transcription Regulation in the Budding Yeast
Source: PLoS One. 2007 Feb 28;2(2):e250. doi: 10.1371/journal.pone.0000250 (PMC1803021; doi:10.1371/journal.pone.0000250)
Supplement: Figure S1 — Growth curves of adh1 and wild type cells (0.42 MB PDF) [file pone.0000250.s001.pdf]

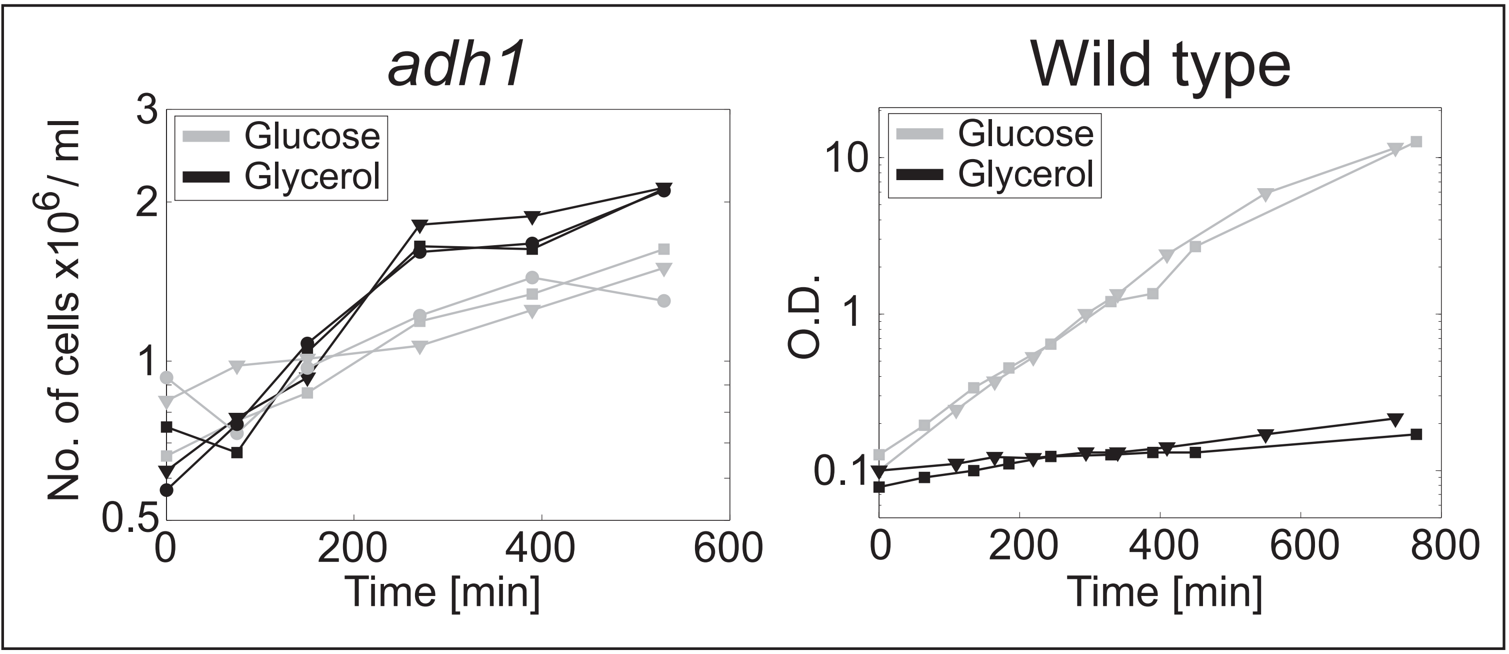

Figure S1. Growth curves of *adh1* mutants **(a)** and wild type cells **(b).** Overnight cell cultures were transferred to glucose (gray) and glycerol (black). Different markers are used to designate different cell cultures.
