# Supplementary material for: Strategy of Transcription Regulation in the Budding Yeast
Source: PLoS One. 2007 Feb 28;2(2):e250. doi: 10.1371/journal.pone.0000250 (PMC1803021; doi:10.1371/journal.pone.0000250)
Supplement: Figure S2 — Response of cells grown in steady state to environmental perturbations (0.46 MB PDF) [file pone.0000250.s002.pdf]

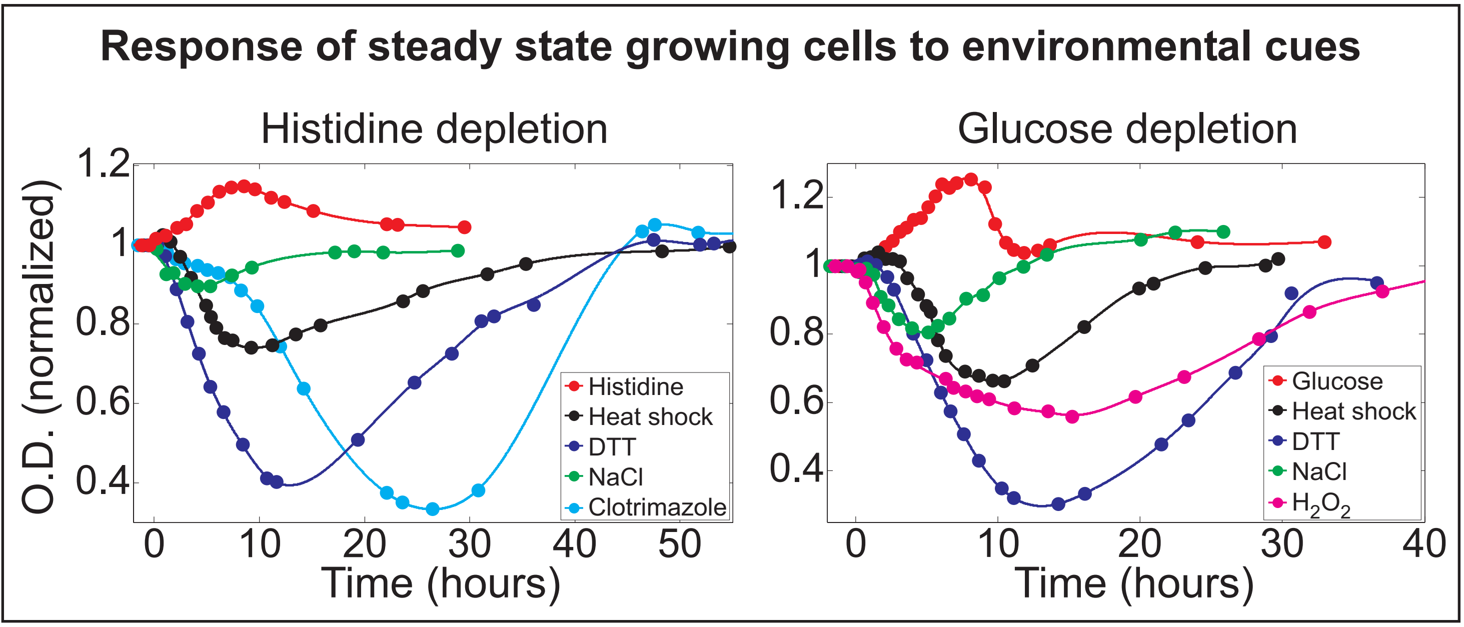

Figure S2. Response of cells grown in steady state to environmental perturbations. The markers designate O.D. sampling time, and solid lines are spline interpolations of the data. The Y-axis is normalized such that the steady state O.D. (at t<0) is 1. Cells were examined in two chemostat environments. Cells in a histidine-limited chemostat were subjected to the following 5 environmental cues: Clotrimazole (10µM - cyan curve), DTT (6mM - blue curve), NaCl (0.31M - green curve), heat shock (37ºC for 5 hours - black curve), and a stress-relieving pulse of histidine (2 mg/l - red curve). Cells in a glucose-limited chemostat were subjected to the following 5 environmental cues: H_2O_2 (0.6mM - magenta curve), DTT (6mM - blue curve), NaCl (0.4M - green curve), heat shock (39ºC for 5 hours - black curve), and a stress-relieving pulse of glucose (0.2 g/l - red curve).
