# Supplementary figures and images for: Strategy of Transcription Regulation in the Budding Yeast
Source: PLoS One. 2007 Feb 28;2(2):e250. doi: 10.1371/journal.pone.0000250 (PMC1803021; doi:10.1371/journal.pone.0000250)

## Average module expression in response to environmental cues

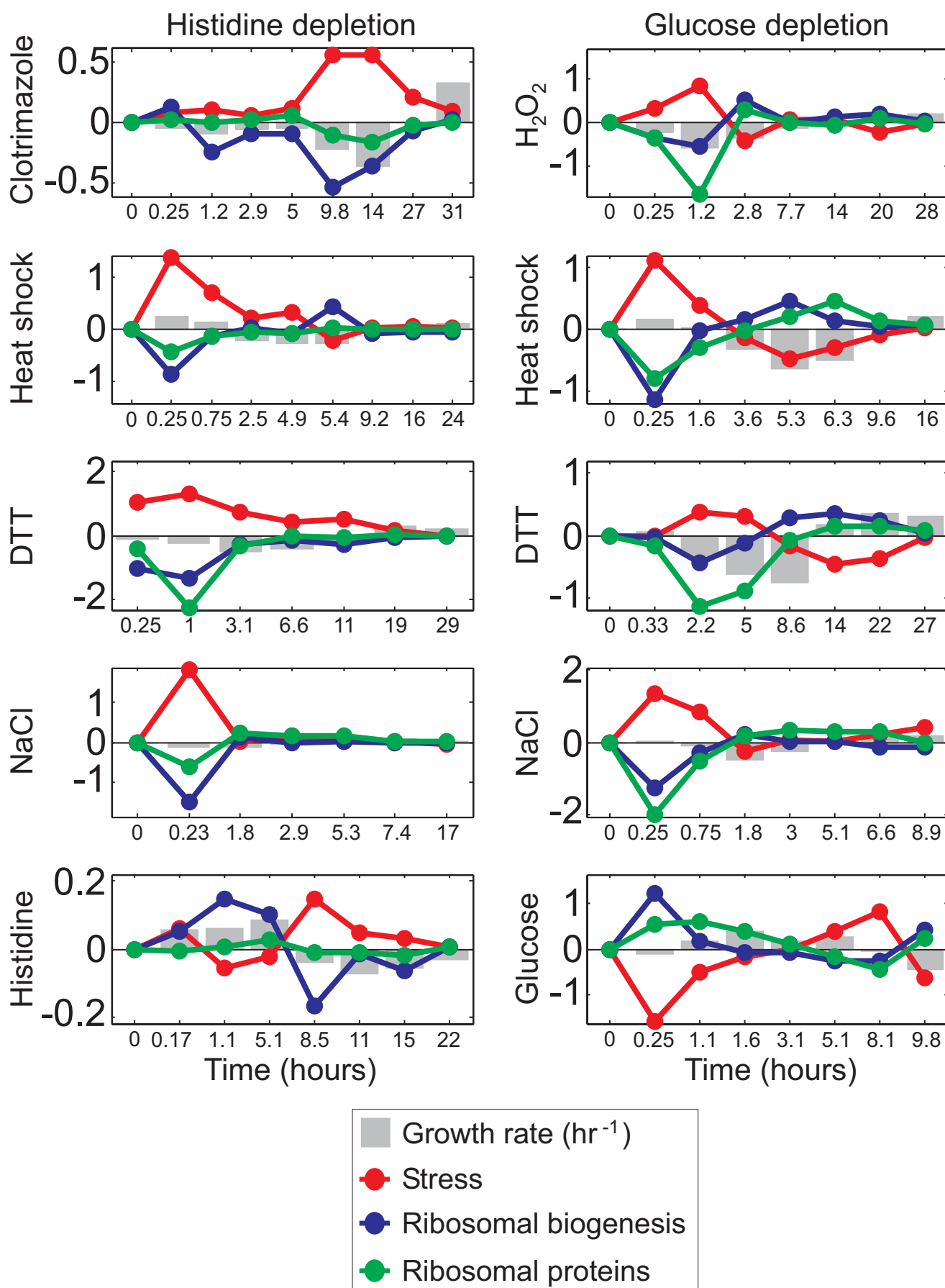

Figure S3

Supplement: Figure S3 — Dynamics of average module expression and cell growth upon environmental perturbations. The average log2-ratio-expression is presented for three ESR gene modules. Bars designate the measured cells growth rate, which is normalized relative to the steady state growth rate. (0.44 MB PDF) [file pone.0000250.s003.pdf]
