# Supplementary material for: Strategy of Transcription Regulation in the Budding Yeast
Source: PLoS One. 2007 Feb 28;2(2):e250. doi: 10.1371/journal.pone.0000250 (PMC1803021; doi:10.1371/journal.pone.0000250)
Supplement: Table S1 — List of gene modules (0.18 MB PDF) [file pone.0000250.s004.pdf]

Stress genes			
**Serial #**	**ORF**	**Name**	**description**
**1**	YAL060W		YAL060W; FUN49 Stereospecific (2R, 3R)-2,3-butanediol dehydrogenase with similarity to alcohol/sorbitol dehydrogenases, member of thezinc-containing alcohol dehydrogenase family
**2**	YAL061W		YAL061W; FUN50 Protein with similarity to alcohol/sorbitol dehydrogenase member of the zinc-containing alcohol dehydrogenase family
**3**	YBL064C		YBL064C; PRX1; YBL0503; MTP1; mTPx YBL0524 Mitochondrial thiol peroxidase
**4**	YBL078C		AUT7; APG8; CVT5; YBL0732; YBL078C Protein that mediates attachment of autophagosomes to microtubules in combination with Aut2p, alsorequired for sporulation
**5**	YBR001C	NTH2	NTH2; YBR0106; YBR001C Putative secondary neutral trehalase (alpha, alpha-trehalase), may catalyze conversion of trehalose to glucose
**6**	YBR006W		UGA2; UGA5; YBR0112; YBR006W Succinate semialdehyde dehydrogenase
**7**	YBR026C	MRF1'	YBR026C; YBR0310; (MRF1) Nuclear protein essential for mitochondrial respiratory function, binds preferentially to single-stranded coresequence of ARS DNA
**8**	YBR052C		YBR052C; YBR0505 Protein with similarity to Ycp4p, S. pombe obr1, and E. coli trp repressor binding protein
**9**	YBR053C		YBR053C; YBR0506 Protein with similarity to rat calcium-binding protein regucalcin and rat senescence marker protein 30
**10**	YBR056W		YBR056W; YBR0510 Protein with similarity to glucan-1,3-beta-glucosidase (exo-1,3-beta-glucanase)
**11**	YBR072W	HSP26	HSP26; YBR0714; YBR072W Heat shock protein of 26 kDa, expressed during entry to stationary phase and induced by osmostress
**12**	YBR126C	TPS1	TPS1; CIF1; FDP1; BYP1; GGS1; GLC6; TSS1; YBR0922; YBR126C Trehalose-6-phosphate synthase, component of trehalose-6-phosphatesynthase/phosphatase complex
**13**	YBR137W		YBR137W; YBR1013 Protein of unknown function
**14**	YBR139W		YBR139W; YBR1015 Protein with similarity to serine-type carboxypeptidases
**15**	YBR149W		ARA1; YBR1127; YBR149W Subunit of NADP+-dependent D-arabinose dehydrogenase
**16**	YBR169C	SSE2	SSE2; YBR1221; YBR169C Heat shock protein of the HSP70 family, present at low abundance at 23 deg but greatly induced after shift to 37 deg
**17**	YBR183W		YPC1; YBR1305; YBR183W Alkaline ceramidase
**18**	YBR204C		YBR204C; YBR1444 Protein with similarity to serine-active lipases, localized to lipid droplets
**19**	YBR214W		SDS24; YBR1501; YBR214W Nuclear protein with similarity to S. pombe sds23/moc1 protein
**20**	YBR230C		YBR230C; YBR1527 Protein of unknown function
**21**	YBR269C		YBR269C; YBR1737 Protein of unknown function
**22**	YBR280C		YBR280C; YBR2017 Protein with similarity to Srm1p/Prp20p
**23**	YCL035C		GRX1; YCL035C Putative glutaredoxin, has similarity to thioltransferase Ttr1p
**24**	YCL040W	GLK1	GLK1; YCL312; YCL040W Glucokinase, specific for aldohexoses
**25**	YCL042W		YCL042W Protein of unknown function

**26**	YCR004C	YCP4	YCP4; YCR042; YCR004C Protein with similarity to S. pombe brefeldin A resistance protein obr1 and E. coli WrbA protein which stimulatesbinding of Trp repressor to DNA
**27**	YCR091W	KIN82	KIN82; YCR1153; YCR091W Serine/threonine protein kinase with unknown role
**28**	YDL021W	GPM2	GPM2; D2835; YDL021W Phosphoglycerate mutase, with similarity to Gpm1p and Gpm3p
**29**	YDL022W	GPD1	GPD1; DAR1; OSG1; OSR5; D2830; YDL022W Glycerol-3-phosphate dehydrogenase (NAD+), cytoplasmic, involved in glycerol productionconverting glycerol-3-phosphate and NAD+ to dihydroxyacetone phosphate and NADH
**30**	YDL027C		YDL027C; D2800 Protein of unknown function
**31**	YDL072C		YDL072C; D2492 Protein of unknown function
**32**	YDL110C		YDL110C; D2320 Protein of unknown function
**33**	YDL124W		YDL124W; D2240 Protein of unknown function
**34**	YDL181W	INH1	INH1; D1305; YDL181W Inhibitor of mitochondrial ATPase that forms a complex with ATP synthase to inhibit enzyme activity
**35**	YDL204W		YDL204W; D1062 Protein of unknown function
**36**	YDL222C		YDL222C; D0850 Protein with similarity to Sur7p
**37**	YDR001C		NTH1; NTH YD8119.07; D2955; YDR001C Neutral trehalase
**38**	YDR003W		YDR003W; D2965; YD8119.09 Protein with similarity to Ybr005p
**39**	YDR031W		YDR031W; D3418 Protein of unknown function
**40**	YDR032C		PST2; D3422; YD9673.02; YDR032C Protein with similarity to Ycp4p and S. pombe OBR1 brefeldin A resistance protein
**41**	YDR070C		YDR070C; YD8554.03; D4275 Protein of unknown function
**42**	YDR074W	TPS2	TPS2; HOG2; PFK3; D4416; YD8554.07; YDR074W Trehalose-6-phosphate phosphatase, component of the trehalose-6-phosphatasesynthase/phosphatase complex
**43**	YDR171W	HSP42	HSP42; YD9395.04; YDR171W Heat shock protein with similarity to Hsp26p, involved in restoration of the cytoskeleton during mild stress
**44**	YDR178W	SDH4	SDH4; YD9395.11; YDR178W Membrane anchor subunit in the succinate dehydrogenase complex
**45**	YDR204W	COQ4	COQ4; YD8142A.01; YD8142.01; YDR204W Protein involved in biosynthesis of coenzyme Q
**46**	YDR231C		COX20; YD9934.15; YDR231C Protein involved in maturation of Cox2p and its assembly into cytochrome c oxidase
**47**	YDR258C	HSP78	HSP78; YD9320A.08; YDR258C Heat shock protein of the ClpB family of ATP-dependent proteases, mitochondrial
**48**	YDR272W	GLO2	GLO2; D9954.5; YDR272W Glyoxalase-II
**49**	YDR343C	HXT6	HXT6; D9651.12; YDR343C High-affinity hexose transporter, member of the hexose transporter family of the major facilitator superfamily (MFS),nearly identical to Hxt7p
**50**	YDR358W		GGA1; D9476.2; YDR358W Protein involved in trafficking of proteins between the trans-Golgi network and the vacuole
**51**	YDR368W	YPR1	YPR1; D9481.8; YDR368W Protein with similarity to members of the aldo/keto reductase family
**52**	YDR391C		YDR391C; D9509.11 Protein of unknown function
**53**	YDR435C		PPM1; D9461.21; YDR435C Carboxy methyltransferase for protein phosphatase 2A catalytic subunit, has similarity to human LCMT

**54**	YDR453C		YDR453C; D9461.38; cTPxII Cytoplasmic thiol peroxidase
**55**	YDR511W		ACN9; D9719.16; YDR511W Protein required for acetate utilization
**56**	YDR512C		YDR512C; D9719.18 Protein of unknown function questionable ORF
**57**	YDR513W	TTR1	TTR1; GRX2; TTR D9719.17; YDR513W Glutaredoxin (thioltransferase, glutathione reductase)
**58**	YDR516C		YDR516C; D9719.21 Protein with similarity to Glk1p
**59**	YDR533C		YDR533C; D9719.36 Protein of unknown function
**60**	YEL011W	GLC3	GLC3; YEL011W alpha-1,4-Glucan branching enzyme
**61**	YEL012W	UBC8	UBC8; GID3; YEL012W Ubiquitin-conjugating enzyme involved in the catabolite degradation of fructose-1,6-bisphosphate
**62**	YEL039C	CYC7	CYC7; (CYC2); (CYP3); YEL039C Cytochrome-c isoform 2, predominant isoform during anaerobic growth
**63**	YER053C		YER053C Member of the mitochondrial carrier (MCF) family of membrane transporters, has similarity to C. elegans mitochondrial phosphate carrierprotein
**64**	YER067W		YER067W Protein of unknown function
**65**	YER079W		YER079W Protein of unknown function, may be involved in signal transduction
**66**	YER103W	SSA4	SSA4; YER103W Protein chaperone of the HSP70 family, cytoplasmic heat-induced form that is not expressed under optimal conditions
**67**	YER141W	COX15	COX15; YER141W Protein required for cytochrome oxidase assembly
**68**	YER142C	MAG1	MAG1; MAG MMS5; YER142C DNA-3-methyladenine glycosylase excises 3-methyladenine, 7-methyladenine, 3-methylguanine and7-methylguanine from alkylation-damaged DNA
**69**	YER150W		SPI1; YER150W Protein induced in stationary phase, has similarity to Sed1p
**70**	YER158C		YER158C Protein with similarity to Afr1p
**71**	YER182W		YER182W Protein of unknown function
**72**	YFL014W	HSP12	HSP12; GLP1; YFL014W Heat shock protein of 12 kDa, induced by heat, osmotic stress, oxidative stress and in stationary phase
**73**	YFR003C		YFR003C Protein of unknown function, may physically interact with Glc7p and Ppz1p
**74**	YFR014C	CMK1	CMK1; YFR014C Calcium/calmodulin-dependent serine/threonine protein kinase (CaM kinase) type I
**75**	YFR015C	GSY1	GSY1; YFR015C UDP-glucose-starch glucosyltransferase (glycogen synthetase) isoform 1
**76**	YFR053C	HXK1	HXK1; HKA YFR053C Hexokinase I, converts hexoses to hexose phosphates in glycolysis repressed by glucose
**77**	YGL006W	PMC1	PMC1; G3749; YGL006W Vacuolar Ca2+-transporting P-type ATPase, member of the cation transporting (E1-E2) P-type ATPase superfamily,functions to pump Ca2+ into the vacuole
**78**	YGL037C		PNC1; G3629; YGL037C Pyrazinamidase and nicotinamidase
**79**	YGL121C		YGL121C; G2913 Protein of unknown function
**80**	YGL156W	AMS1	AMS1; G1861; YGL156W alpha-Mannosidase, hydrolyzes terminal non-reducing alpha-D-mannose residues from alpha-D-mannosides
**81**	YGL248W	PDE1	PDE1; NRB369; G0574; YGL248W 3',5'-Cyclic-nucleotide phosphodiesterase, low affinity

**82**	YGR008C	STF2	STF2; G3858; YGR008C ATPase stabilizing factor, binds to F0-ATPase facilitates binding of inhibitor and 9 kDa protein to F1-ATPase
**83**	YGR019W	UGA1	UGA1; G4027; YGR019W 4-Aminobutyrate aminotransferase
**84**	YGR028W	MSP1	MSP1; YTA4; G4060; YGR028W Intra-mitochondrial sorting protein, member of the AAA family of ATPases
**85**	YGR043C		YGR043C; G4301 Protein of unknown function, may be involved in signal transduction has strong similarity to Tal1p
**86**	YGR052W		YGR052W; G4329 Serine/threonine protein kinase of unknown function
**87**	YGR088W	CTT1	CTT1; G4628; YGR088W Catalase T (cytosolic)
**88**	YGR111W		YGR111W; G6145 Protein with similarity to southern house mosquito carboxylesterase PIR:S53372
**89**	YGR127W		YGR127W; G6370 Protein with similarity to mouse T10 protein PIR:S37448
**90**	YGR130C		YGR130C; G6382 Protein with similarity to Entamoeba histolytica myosin heavy chain PIR:L03534
**91**	YGR132C	PHB1	PHB1; PHB G6390; YGR132C Prohibitin, involved in determination of replicative lifespan member of the prohibitin complex with Phb2p
**92**	YGR149W		YGR149W; G6639 Protein with similarity to hypothetical protein A-288
**93**	YGR174C	CBP4	CBP4; G7122; YGR174C Ubiquinol-cytochrome c reductase assembly factor essential for assembly and stability of ubiquinol-cytochrome creductase
**94**	YGR194C		XKS1; G7584; YGR194C Xylulokinase, required for growth on D-xylulose
**95**	YGR201C		YGR201C; G7727 Protein with similarity to translation elongation factors
**96**	YGR237C		YGR237C; ORF785; G8581 Protein of unknown function, contains a UspA ATP-binding domain
**97**	YGR243W		YGR243W; G8620 Protein of unknown function
**98**	YGR244C		LSC2; G8625; YGR244C Succinyl-CoA ligase beta subunit of succinyl-CoA synthetase complex which has succinyl-CoA ligase and succinatethiokinase activities
**99**	YGR248W	SOL4	SOL4; G9125; YGR248W Possible 6-phosphogluconolactonase
**100**	YGR250C		YGR250C; G9135 Protein with three RNA recognition motif (RRM) domains, has similarity to human 64K polyadenylation factor
**101**	YGR255C	COQ6	COQ6; G9165; YGR255C Monooxygenase required for coenzyme Q (ubiquinone) biosynthesis
**102**	YGR256W	GND2	GND2; G9170; YGR256W 6-Phosphogluconate dehydrogenase, decarboxylating, converts 6-phosphogluconate + NADP to ribulose-5-phosphate+ NADPH + CO2
**103**	YHL021C		YHL021C Protein of unknown function
**104**	YHR016C	YSC84	YSC84; LSB4; YHR016C Protein with possible role in the regulation of actin cytoskeletal organization, has an SH3 domain
**105**	YHR087W		YHR087W Protein of unknown function
**106**	YHR096C	HXT5	HXT5; YHR096C Member of the hexose transporter family of the major facilitator superfamily (MFS)
**107**	YHR097C		YHR097C Protein of unknown function
**108**	YHR104W	GRE3	GRE3; H8263.12; YHR104W Aldose reductase with NADPH specificity induced by osmotic stress
**109**	YHR112C		YHR112C; H8263.18 Protein with similarity to cystathionine gamma-synthase Str2p and other transulfuration enzymes

**110**	YHR113W		YHR113W; H8263.5 Protein with similarity to vacuolar aminopeptidase Lap4p/Ape1p
**111**	YHR138C		YHR138C Protein possibly involved in vacuolar fusion, has similarity to Pbi2p, which is a protease B inhibitor
**112**	YHR140W		YHR140W Protein of unknown function, has 6 potential transmembrane segments
**113**	YHR171W		APG7; CVT2; YHR171W Apg12p-activating enzyme, involved in autophagy, cytoplasm-to-vacuole protein targeting, and peroxisome degradationpathways
**114**	YIL045W	PIG2	PIG2; YI9905.03; YIL045W Protein that interacts with Gsy2p, possible regulatory subunit for the PP1-family protein phosphatase Glc7p
**115**	YIL087C		YIL087C; YI9910.09 Protein of unknown function
**116**	YIL097W		FYV10; YIL097W Protein of unknown function
**117**	YIL101C	XBP1	XBP1; YIL101C Stress-induced transcriptional repressor
**118**	YIL107C	PFK26	PFK26; YIL107C 6-Phosphofructose-2-kinase, isozyme 1
**119**	YIL111W	COX5B	COX5B; YIL111W Cytochrome c oxidase chain Vb, expressed under anaerobic conditions
**120**	YIL113W		YIL113W Dual-specificity protein phosphatase
**121**	YIL124W		AYR1; YI8277.05; YIL124W 1-Acyl dihydroxyacetone phosphate reductase
**122**	YIL136W	OM45	OM45; YIL136W Protein of the outer mitochondrial membrane
**123**	YIR037W	HYR1	HYR1; GPX3; YIR037W Glutathione peroxidase involved in oxidative stress response
**124**	YIR038C		GTT1; YIR038C Glutathione transferase
**125**	YIR039C		YPS6; YIR039C Yapsin 6, GPI-anchored aspartyl protease
**126**	YJL021C		YJL021C; J1286 Protein of unknown function
**127**	YJL048C		YJL048C; J1164 Protein of unknown function
**128**	YJL057C	IKS1	IKS1; J1143; YJL057C Probable serine/threonine protein kinase
**129**	YJL066C		MPM1; HRE252; J1111; YJL066C Protein of unknown function
**130**	YJL067W		YJL067W; HRA116; J1107 Protein of unknown function
**131**	YJL068C		YJL068C; HRE299; J1102 Esterase, may be involved in conversion of formaldehyde to S-formylglutathione
**132**	YJL103C		YJL103C; J0824 Protein with similarity to transcription factors, has Zn[2]-Cys[6] fungal-type binuclear cluster domain in the N-terminal region
**133**	YJL144W		YJL144W; J0646 Protein of unknown function
**134**	YJL151C		YJL151C; J0630 Protein of unknown function, has similarity to Ydr525W-Ap and Ydl123p
**135**	YJL155C	FBP26	FBP26; J0575; YJL155C Fructose-2,6-bisphosphatase
**136**	YJL161W		YJL161W; J0552 Protein of unknown function
**137**	YJL163C		YJL163C; J0544 Protein of unknown function
**138**	YJL164C	TPK1	TPK1; SRA3; PKA1; PK25; J0541; YJL164C Catalytic subunit of cAMP-dependent protein kinase 1, protein kinase A or PKA
**139**	YJR008W		YJR008W; YJR83.9; J1431 Protein of unknown function
**140**	YJR073C	OPI3	OPI3; PEM2; YJR073C Phospholipid-N-methyltransferase, carries out the second and third methylation steps of the phosphatidylcholinebiosynthesis pathway

**141**	YJR085C		YJR085C; J1863 Protein of unknown function
**142**	YJR096W		YJR096W; J1926 Protein with similarity to aldolase reductase
**143**	YKL007W	CAP1	CAP1; YKL155; YKL007W Actin-capping protein, alpha subunit
**144**	YKL026C		GPX1; YKL026C Glutathione peroxidase
**145**	YKL035W		UGP1; YKL035W; YKL248 UDP-glucose pyrophosphorylase (UTP-glucose-1-P uridylyltransferase)
**146**	YKL065C	YET1	YET1; YKL331; YKL065C Transmembrane protein of the endoplasmic reticulum
**147**	YKL067W	YNK1	YNK1; YNK NDK1; YKL333; YKL067W Nucleoside diphosphate kinase, responsible for synthesis of all nucleoside triphosphates except ATP
**148**	YKL085W	MDH1	MDH1; ACN50; YKL085W Malate dehydrogenase, mitochondrial
**149**	YKL091C		YKL091C; (SFH1) Protein with similarity to Sec14p
**150**	YKL100C		YKL100C; YKL450 Protein of unknown function
**151**	YKL103C	LAP4	LAP4; APE1; API YSC1; YKL455; YKL103C Aminopeptidase I (yscI, API) of the vacuole
**152**	YKL133C		YKL133C Protein of unknown function
**153**	YKL141W	SDH3	SDH3; CYB3; YKL4; YKL141W Membrane anchor subunit for Sdh1p in the succinate dehydrogenase complex
**154**	YKL142W	MRP8	MRP8; YKL3; YKL142W Mitochondrial ribosomal protein of the small subunit
**155**	YKL148C	SDH1	SDH1; SDHA HAR2; YKL602; YKL148C Succinate dehydrogenase (ubiquinone) flavoprotein (Fp) subunit, converts succinate plus ubiquinoneto fumarate plus ubiquinol in the TCA cycle
**156**	YKL150W	MCR1	MCR1; YKL605; YKL150W NADH-cytochrome b5 reductase
**157**	YKL151C		YKL151C; YKL606 Protein of unknown function
**158**	YKL193C	SDS22	SDS22; EGP1; YKL193C Regulatory subunit for Glc7p type I protein serine/threonine phosphatase with an essential function in late mitosis
**159**	YKR011C		TOS5; YK111; YKR011C Protein of unknown function
**160**	YKR049C		YKR049C Protein of unknown function
**161**	YKR058W	GLG1	GLG1; YKR058W Self-glucosylating initiator of glycogen synthesis
**162**	YKR067W		YKR067W Protein with similarity to Sct1p
**163**	YKR076W	ECM4	ECM4; YKR076W Protein possibly involved in cell wall structure or biosynthesis
**164**	YLL020C		YLL020C; L1219 Protein of unknown function
**165**	YLL023C		YLL023C; L1201 Protein of unknown function
**166**	YLL026W	HSP104	HSP104; L0948; YLL026W Heat shock protein required for induced thermotolerance and for resolubilizing aggregates of denatured proteins,important for the [psi-] to [PSI+] prion conversion
**167**	YLL039C	UBI4	UBI4; SCD2; L0904; YLL039C Ubiquitin polyprotein, mature ubiquitin is cleaved from polyubiquitin (Ubi4p) or from fusions with ribosomalproteins Rps31p, Rpl40Ap, or Rpl40Bp
**168**	YLL041C	SDH2	SDH2; SDHB SDH L0745; YLL041C Succinate dehydrogenase (ubiquinone) iron-sulfur protein (Ip) subunit, converts succinate + ubiquinone tofumarate + ubiquinol in the TCA cycle
**169**	YLR080W		YLR080W; L9449.7; L2365 Protein with similarity to Emp47p
**170**	YLR149C		YLR149C; L9634.7; L3317 Protein of unknown function

**171**	YLR177W		YLR177W; L9470.10 Protein with similarity to Psp1p
**172**	YLR178C	TFS1	TFS1; DKA1; (NSP1); L9470.19; YLR178C CDC25-dependent nutrient- and ammonia-response cell cycle regulator, binds to and inhibits Prc1p(carboxypeptidase Y)
**173**	YLR251W		YLR251W; L9672.3 Protein with similarity to mouse MPV17 protein
**174**	YLR252W		YLR252W Protein of unknown function
**175**	YLR258W	GSY2	GSY2; L8479.8; YLR258W UDP-glucose-starch glucosyltransferase (glycogen synthetase) isoform 2
**176**	YLR270W		YLR270W; L8479.2 Protein of unknown function
**177**	YLR299W	ECM38	ECM38; CIS2; L8003.4; YLR299W Putative gamma-glutamyltransferase involved in glutathione synthesis
**178**	YLR327C		YLR327C; L8543.1 Protein with strong similarity to Stf2p
**179**	YLR345W		YLR345W; L8300.3 Protein with similarity to rat fructose-2,6-bisphosphatase
**180**	YLR356W		YLR356W; L9638.2 Protein with similarity to Scm4p, possible Cdc4p-interacting protein
**181**	YLR370C	ARC18	ARC18; L8039.15; YLR370C Component of the ARP2/3 actin-organizing complex involved in actin assembly and function
**182**	YML004C	GLO1	GLO1; YM9571.15; YML004C Glyoxalase I, catalyzes condensation of hemimercaptal adduct of methylglyoxal with glutathione to formS-D-lactoylglutathione
**183**	YML054C	CYB2	CYB2; YM9958.08; YML054C Cytochrome b2 [L-(+)-lactate cytochrome c oxidoreductase] converts L-lactate to pyruvate
**184**	YML100W	TSL1	TSL1; YML100W Component of the trehalose-6-phosphate synthase/phosphatase complex alternate third subunit with Tps3p
**185**	YML110C	DBI56	COQ5; TCM7; YM8339.09; YML110C; DBI56 Mitochondrial C-methyltransferase of the ubiquinone biosynthetic pathway
**186**	YML120C	NDI1	NDI1; YM7056.06; YML120C NADH-ubiquinone oxidoreductase
**187**	YML128C		MSC1; YM4987.07; YML128C Protein of unknown function that affects meiotic homologous chromatid recombination
**188**	YMR090W		YMR090W; YM9582.15 Protein with similarity to malate dehydrogenases
**189**	YMR105C	PGM2	PGM2; GAL5; YM9718.04; YMR105C Phosphoglucomutase major isozyme, interconverts Glc-1-P and Glc-6-P
**190**	YMR110C		YMR110C; YM9718.09 Protein with similarity to aldehyde dehydrogenases
**191**	YMR139W	RIM11	RIM11; MDS1; GSK3; YM9375.08; YMR139W Member of the GSK-3 sub-family of protein kinases (which includes Mck1p, Rim11p, Mrk1p, andYOL128Cp), required for induction of IME2 by Ime1p
**192**	YMR152W		YIM1; YM9375.22; YM8520.01; YMR152W Mitochondrial inner membrane protease, has sequence similarity to E. coli leader peptidase
**193**	YMR169c	ALD3	ALD3; YM8520.18; YMR169C Cytoplasmic, stress inducible aldehyde dehydrogenase, probable isoform of Ald2p
**194**	YMR170C	ALD2	ALD2; (ALD5); YM8520.19; YMR170C Aldehyde dehydrogenase (NAD(P)+), likely cytosolic
**195**	YMR173W	DDR48	DDR48; FSP YM8010.03; YMR173W Stress protein induced by heat shock, DNA damage, or osmotic stress
**196**	YMR174c	PAI3	PAI3; YM8010.04; YMR174C Proteinase A (yscA) inhibitor IA3
**197**	YMR181C		YMR181C; YM8010.11 Protein of unknown function
**198**	YMR196W		YMR196W; YM9646.09 Protein of unknown function

**199**	YMR197C	VTI1	VTI1; YM9646.10; YMR197C Synaptobrevin (v-SNARE) homolog, has an essential role at the cis-Golgi stage and a non-essential role at the Golgito prevacuole stage
**200**	YMR250W		GAD1; YM9920.04; YMR250W Glutamate decarboxylase
**201**	YMR291W		YMR291W Serine/threonine protein kinase of unknown function
**202**	YMR297W	PRC1	PRC1; LBC1; SSU7; CPY YMR297W Carboxypeptidase Y (CPY/yscY), a serine protease
**203**	YMR302C	PRP12	RNA12; PRP12; YME2; YM9952.04; YMR302C Protein with a role in early maturation of pre-rRNA and mitochondrial maintenance
**204**	YMR315W		YMR315W; YM9924.07 Protein of unknown function
**205**	YNL015W	PBI2	PBI2; N2844; YNL015W Protease B (yscB or Prb1p) inhibitor 2 (I2B), has activity related to vacuolar fusion that is not related to protease activity
**206**	YNL055C	POR1	POR1; OMP2; VDAC N2441; YNL1624; YNL2441; YNL055C Outer mitochondrial membrane porin (voltage-dependent anion-selective channel)
**207**	YNL115C		YNL115C; N1929 Protein of unknown function
**208**	YNL134C		YNL134C; N1214; N1847 Protein with similarity to C. carbonum toxD gene
**209**	YNL156C		YNL156C; N1747 Protein of unknown function
**210**	YNL160W	YGP1	YGP1; N1731; YNL160W Secreted glycoprotein produced in response to nutrient limitation
**211**	YNL173C	MDG1	MDG1; N1673; YNR3; YNL173C Protein involved in signal transduction in the mating response
**212**	YNL200C		YNL200C; N1370 Protein of unknown function
**213**	YNL274C		YNL274C; N0631 Potential alpha-ketoisocaproate reductase
**214**	YNL305C		YNL305C; YNL0405; N0405 Protein with similarity to Aspergillus nidulans negative-acting regulatory protein qutR and Drosophila melanogasterN-methyl-D-aspartate receptor-associated protein
**215**	YNR001C	CIT1	CIT1; GLU3; N2019; YNR001C Citrate synthase, mitochondrial, converts acetyl-CoA and oxaloacetate into citrate plus CoA
**216**	YOL032W		YOL032W; O2133 Protein of unknown function
**217**	YOL048C		YOL048C; O1292 Protein of unknown function
**218**	YOL071W		YOL071W; O1145 Protein of unknown function
**219**	YOL082W		YOL082W; YOL0950; O0980 Protein of unknown function
**220**	YOL083W		YOL083W; O0957 Protein of unknown function
**221**	YOL084W		PHM7; O0953; YOL084W Protein of unknown function, transcriptionally regulated by phosphate
**222**	YOL129W		YOL129W; O0526 Protein of unknown function
**223**	YOL151W	GRE2	GRE2; AOA342; O0446; YOL151W Protein involved in diamide tolerance and induced by osmotic stress, member of a family (GRE2, YGL039W,YGL157W, YDR541C) with similarity to plant dihydroflavonol-4-reductases
**224**	YOL153C		YOL153C Protein of unknown function, has strong similarity to the carboxypeptidase Cps1p
**225**	YOR036W	PEP12	PEP12; VPL6; VPT13; VPS6; OR26.29; O2750; YOR036W Syntaxin homolog (t-SNARE) involved in Golgi to vacuole transport
**226**	YOR052C		YOR052C; YOR29-03; O2796 Protein of unknown function

**227**	YOR120W	GCY1	GCY1; GCY O31567; YOR3269; O3269; YOR120W Galactose-induced protein with strong similarity to crystallin protein of vertebrate eye lens
**228**	YOR121C		YOR121C; O3272 Protein of unknown function
**229**	YOR161C		YOR161C; O3568 Protein of unknown function
**230**	YOR173W		YOR173W; O3625 Protein of unknown function
**231**	YOR185C	GSP2	GSP2; (SSP101); CNR2; O4734; YOR185C Ran, a GTP-binding protein member of the ras superfamily involved in trafficking through nuclearpores
**232**	YOR215C		YOR215C; YOR50-5; O5005 Protein of unknown function
**233**	YOR220W		YOR220W; YOR50-10; O5010; O5050 Protein of unknown function
**234**	YOR273C		YOR273C; O5440 Member of the multidrug-resistance 12-spanner (DHA12) family of the major facilitator superfamily (MFS-MDR)
**235**	YOR285W		YOR285W; O5486W Protein with similarity to Drosophila melanogaster heat shock protein 67B2
**236**	YOR289W		YOR289W; O5498W Protein of unknown function
**237**	YOR317W	FAA1	FAA1; O6136; YOR317W Long-chain fatty acid CoA ligase (fatty acid activator 1) can incorporate exogenous myristate into myristoyl-CoA andother fatty acids to the CoA derivatives
**238**	YOR347C	PYK2	PYK2; O6342; YOR347C Pyruvate kinase, glucose-repressed isoform
**239**	YOR374W	ALD7	ALD4; ALD7; O6730; YOR374W Mitochondrial aldehyde dehydrogenase
**240**	YOR386W	PHR1	PHR1; O6771; YOR386W Deoxyribodipyrimidine photolyase involved in light-dependent repair of pyrimidine dimers
**241**	YPL004C		YPL004C; YP8132.09; LPA13 Protein with weak similarity to tropomyosin
**242**	YPL087W		YDC1; LPG21; YPL087W Alkaline ceramidase with specificity for dihydroceramide
**243**	YPL123C		RNY1; LPH4; YPL123C Ribonuclease of the ribonuclease T2 family
**244**	YPL154C	PEP4	PEP4; PRA1; PHO9; P2585; YPL154C Proteinase A (PrA/yscA/saccharopepsin) aspartyl protease required for activation of various degradativeenzymes
**245**	YPL165C		YPL165C; P2545 Protein of unknown function
**246**	YPL186C		YPL186C; P2213 Protein of unknown function
**247**	YPL196W		YPL196W; P1905 Protein of unknown function
**248**	YPL203W	TPK2	TPK2; PKA2; PKA3; YKR1; P1855; YPL203W Catalytic subunit of cAMP-dependent protein kinase 2, protein kinase A or PKA
**249**	YPL230W		USV1; P1421; YPL230W Putative finger transcripton factor, has a two tandem C2H2-type zinc fingers
**250**	YPL247C		YPL247C; P1024 Protein of unknown function, has WD (WD-40) repeats
**251**	YPR026W	ATH1	ATH1; YP9367.06; YPR026W Vacuolar acid trehalase, converts alpha, alpha-trehalase to glucose
**252**	YPR160W	GPH1	GPH1; P9584.1; YPR160W Glycogen phosphorylase, releases alpha-D-glucose-1-phosphate from glycogen
**253**	YPR172W		YPR172W; P9705.8 Protein of unknown function
**254**	YPR184W		GDB1; P9705.2; YPR184W Glycogen debranching enzyme (4-alpha-glucanotransferase or oligo-1, 4 - 1, 4-glucantransferase)
**255**	YMR251WA		HOR7; M.A59; YMR251W-A Protein involved in responsiveness to hyperosmolarity

Ribosomal biogenesis genes			
**Serial #**	**ORF**	**Name**	**description**
**1**	YAL025C	MAK16	MAK16; YAL025C Nuclear protein with HMG-like acidic region, required for propagation of M1 double-stranded RNA
**2**	YBL024W		NCL1; TRM4; YBL0437; YBL024W Methyltransferase that methylates cytidine to 5-methyl-cytidine (m5C) at several positions in differenttRNAs, has similarity to human proliferating cell nucleolar antigen (p120), a proliferation antigen of human tumors
**3**	YBL028C		YBL028C; YBL0423 Protein of unknown function
**4**	YBL039C	URA7	URA7; YBL0410; YBL039C CTP synthase final step in pyrimidine biosynthesis pathway
**5**	YBL054W		YBL054W; (YBL0509) Protein of unknown function
**6**	YBL068W	PRS4	PRS4; YBL0619; YBL068W; PRPS4 Phosphoribosylpyrophosphate synthetase (ribose-phosphate pyrophosphokinase), enxyme thatsynthesizes phosphoribosylpyrophosphate (PRPP) from ribose-5' phosphate and ATP
**7**	YBR034C	HMT1	HMT1; ODP1; RMT1; YBR0320; YBR034C Protein arginine methyltransferase
**8**	YBR142W	MAK5	MAK5; YBR1119; YBR142W Probable pre-mRNA splicing RNA-helicase of the DEAD box family, involved in maintenance of M double-strandedRNA (dsRNA) killer plasmid
**9**	YBR155W		CNS1; YBR1205; YBR155W Component of the Hsp90 chaperone complex, has tetratricopeptide (TPR) repeats
**10**	YBR247C	ENP1	ENP1; MEG1; YBR1635; YBR247C Essential nuclear protein with effects on N-glycosylation of proteins in the secretory pathway
**11**	YBR266C		YBR266C; YBR1735 Protein of unknown function
**12**	YBR267W		YBR267W; YBR1736 Protein of unknown function, has a single C2H2-type zinc finger
**13**	YBR271W		YBR271W; YBR1739 Putative SAM-dependent methyltransferase
**14**	YCL054W		SPB1; YCL431; YCL054W Putative S-adenosyl-methionine-dependent methyltransferase required for synthesis of 60S ribosomal subunit
**15**	YCL059C	KRR1	KRR1; YCL059C Protein essential for cell division and spore germination
**16**	YCR016W		YCR016W Protein of unknown function
**17**	YCR057C	PWP2	PWP2; YCR055C; YCR058C; YCR057C Essential protein required for cell separation, has eight WD (WD-40) repeats
**18**	YCR072C		YCR072C Protein of unknown function, member of WD (WD-40) repeat family
**19**	YDL031W		DBP10; D2770; YDL031W Putative RNA helicase involved in ribosome biogenesis, has similarity to RNA helicases of DEAD/DEAH box family
**20**	YDL050C		YDL050C; D2590 Protein of Unknown Function questionable ORF
**21**	YDL051W	YLA1	LHP1; YLA1; lhp1; LAH1; D2585; YDL051W Protein homolog to human La autoantigen which binds to and stabilizes pre-tRNA for 3'endonucleolytic cleavage, has an RNA recognition (RRM) domain
**22**	YDL060W		YDL060W; D2544 Protein of unknown function
**23**	YDL062W		YDL062W; D2540 Protein of unknown function, questionable ORF

**24**	YDL063C		YDL063C; D2536 Protein of unknown function
**25**	YDL150W	RPC53	RPC53; RPC4; D1557; YDL150W RNA polymerase III, fourth-largest essential subunit (C53)
**26**	YDL152W		YDL152W; D1551 Protein of unknown function
**27**	YDL153C	SAS10	SAS10; D1545; YDL153C Protein that derepresses HMR, HML and telomeres when overproduced
**28**	YDL167C	NRP1	NRP1; (ARP1); ARP D1478; YDL167C Asparagine-rich protein with one RNA recognition (RRM) domain
**29**	YDL201W		YDL201W; D1075 Putative methyltransferase, highly conserved among eukaryotes
**30**	YDR083W		RRP8; YDR083W; YD8554.16; D4461 Protein involved in cleavage at site A2 in pre-rRNA in the pathway of ribosomal RNA processing
**31**	YDR087C		RRP1; YD8554.20; D4478; YDR087C Protein involved in maturation of 25S rRNA
**32**	YDR091C		RLI1; YD6652.03; YDR091C Putative ortholog of human Rnase L inhibitor (RLI) of the interferon-regulated 2-5A pathway, putative ortholog of C.elegans Y39E4B.1, member of the non-transporter group in the ATP-binding cassette (ABC) superfamily
**33**	YDR101C		YDR101C; YD8557.10 Protein of unknown function
**34**	YDR120C	TRM1	TRM1; YD9727.15; YDR120C N2,N2-dimethylguanine tRNA methyltransferase, required for methylation of G26 of both mitochondrial andcytoplasmic tRNAs
**35**	YDR161W	TCI1	TCI1; YD8358.15; YDR161W Protein that interacts with protein phosphatase 2C
**36**	YDR165W		YDR165W; YD8358.19 Protein of unknown function
**37**	YDR184C		ATC1; (BAT1); LIC4; YD9395.18; YDR184C Protein involved in cation homeostasis interacts with Bud6p/Aip3p
**38**	YDR299W		BFR2; D9740.7; YDR299W Protein involved in a protein transport step that is blocked by brefeldin A
**39**	YDR324C		YDR324C; D9798.12 Protein with multiple WD40 repeats
**40**	YDR361C		BCP1; D9476.12; YDR361C Protein of unknown function
**41**	YDR365C		YDR365C; D9481.12 Protein of unknown function
**42**	YDR395W	SXM1	SXM1; KAP108; D9509.15; YDR395W Beta-karyopherin involved in nuclear protein import of mRNA-binding proteins
**43**	YDR398W		YDR398W; D9509.17 Protein of unknown function
**44**	YDR412W		YDR412W; D9461.3 Cytoplasmic protein of unknown function
**45**	YDR429C	TIF35	TIF35; D9461.16; YDR429C Translation initiation factor eIF3, p33 subunit, contains an RRM (RNA recognition motif) domain
**46**	YDR449C		YDR449C; D9461.34 Protein of unknown function, localized to the nucleus
**47**	YDR465C		RMT2; D8035.9; YDR465C Putative protein arginine methyltransferase has similarity to human guanidinoacetate N-methyltransferase
**48**	YDR496C		YDR496C; D9719.2 Protein of unknown function
**49**	YEL026W		SNU13; YEL026W Component of the U4/U6.U5 snRNP, has similarity to nucleolar snoRNP protein Nhp2p
**50**	YER006W		YER006W Nuclear protein of unknown function, has similarity to mouse Mmr1 protein
**51**	YER025W	GCD11	GCD11; SUI4; YER025W Translation initiation factor eIF2 gamma subunit
**52**	YER036C		KRE30; YER036C Member of the non-transporter group of the ATP-binding cassette (ABC) superfamily
**53**	YER049W		YER049W Component of NuA3 histone acetyltransferase complex

**54**	YER082C		KRE31; YER082C Protein of unknown function, has two tandem C2H2-type zinc fingers, has one WD (WD-40) domain
**55**	YER110C	KAP123	KAP123; YRB4; YER110C Karyopherin-beta involved in nuclear import of ribosomal proteins
**56**	YER126C		KRE32; SYGP-ORF47; YER126C Nuclear protein of unknown function
**57**	YER127W	LCP5	LCP5; SYGP-ORF43; YER127W Protein required for ribosomal RNA processing
**58**	YFL002C	SPB4	SPB4; YFL002C ATP-dependent RNA helicase of DEAH box family required for processing of 25S ribosomal RNA precursor
**59**	YFR001W		LOC1; YFR001W Protein that binds double-stranded RNA, involved in localization of mRNA
**60**	YGL029W		CGR1; G3655; YGL029W Predicted coiled-coil protein that may play a role in ribosome biogenesis, down-regulated after diauxic shift
**61**	YGL078C	DBP3	DBP3; G3210; YGL078C ATP-dependent RNA helicase CA3; member of the DEAD/DEAH box family
**62**	YGL099W		KRE35; G3113; YGL099W Protein required for normal growth, morphology, mating, and sporulation, has similarity to human possible GTP-bindingprotein HSR1
**63**	YGL111W		YGL111W; G2990 Protein of unknown function
**64**	YGL120C	PRP43	PRP43; JA1; (PRP40); G2916; YGL120C Pre-mRNA splicing factor, member of the DEAH-box RNA helicase family
**65**	YGL169W	SUA5	SUA5; G1660; YGL169W Protein involved in translation initiation, mutation suppresses the effect of an aberrant upstream ATG in CYC1
**66**	YGL171W	ROK1	ROK1; G1651; YGL171W ATP-dependent RNA helicase required for rRNA processing, member of DEAD box family
**67**	YGR103W		YGR103W; G5933 Nuclear protein of unknown function with similarity to zebrafish pescadillo
**68**	YGR123C	PPT1	PPT1; G6347; YGR123C Protein serine/threonine phosphatase of unknown function, member of the PPP family of protein phosphatases andrelated to PP5 phosphatases, has tetratricopeptide (TPR) repeats
**69**	YGR128C		YGR128C; G6374 Protein with similarity to Haemophilus glutamate-ammonia-ligase adenylyltransferase (glnE) PIR:G64046
**70**	YGR145W		YGR145W; G6623 Component of NuA3 histone acetyltransferase complex
**71**	YGR159C	NSR1	NSR1; (SHE5); G7001; YGR159C Nucleolar protein involved in processing 20S to 18S rRNA, has 2 RNA recognition (RRM) domains and ismember of GAR (glycine/arginine-rich repeats) family of proteins
**72**	YGR160W		FYV13; G7004; YGR160W Protein of unknown function
**73**	YGR162W	TIF4631	TIF4631; eIF4G1; G7036; YGR162W mRNA cap-binding protein (eIF4F) 150K subunit
**74**	YGR173W		YGR173W; G7112 Protein with similarity to human GTP-binding protein PIR:A55014
**75**	YGR187C	HGH1	HGH1; G7538; YGR187C Protein with similarity to human HMG1 and HMG2 proteins
**76**	YGR200C		ELP2; G7725; YGR200C 90 kDa subunit of elongator and elongating RNA polymerase II holoenzyme, has WD (WD-40) repeats
**77**	YGR245C		SDA1; G9101; YGR245C Essential protein required for normal organization of the actin cytoskeleton
**78**	YGR264C	MES1	MES1; G9340; YGR264C Methionyl-tRNA synthetase, cytoplasmic, member of class I aminoacyl tRNA synthetase family
**79**	YGR272C		YGR272C; G9368 Protein of unknown function

**80**	YGR280C		YGR280C; G9534 Protein of unknown function
**81**	YGR283C		YGR283C; G9544 Protein of unknown function
**82**	YHL011C	PRS3	PRS3; SIW17; YHL011C; PRPS3 Phosphoribosylpyrophosphate synthetase (ribose-phosphate pyrophosphokinase II) component of yeast 20Sproteasome, with a role in cell cycle regulation
**83**	YHL039W		YHL039W Protein with weak similarity to protein lysine methyltransferases
**84**	YHR020W		YHR020W; DED82 Protein with similarity to prolyl-tRNA synthetases, member of the class II aminoacyl-tRNA synthetases
**85**	YHR052W		YHR052W Protein of unknown function
**86**	YHR065C	RRP3	RRP3; H8025.18; YHR065C Helicase required for maturation of pre-rRNA
**87**	YHR066W	SSF1	SSF1; YHR066W Protein with a potential role in mating
**88**	YHR088W		RPF1; YHR088W Protein of unknown function, may be involved processing of precursor rRNAs
**89**	YHR089C	GAR1	GAR1; YHR089C Protein involved in 35S rRNA processing and pseudouridinylation associated with the H/ACA class small nucleolar RNAs
**90**	YHR148W		IMP3; YHR148W Component of U3 snoRNP, required for pre-18S rRNA processing
**91**	YHR169W	DBP8	DBP8; YHR169W Protein with similarity to DEAD box family of RNA helicases
**92**	YHR170W	NMD3	NMD3; SRC5; YHR170W Protein involved in the nuclear export of 60S ribosomal subunits
**93**	YHR196W		YHR196W Protein of unknown function
**94**	YHR197W		YHR197W Component of NuA3 histone acetyltransferase complex
**95**	YIL019W		YIL019W; YI3299.12 Protein of unknown function, has potential coiled-coil region
**96**	YIL064W		YIL064W Predicted methyltransferase protein
**97**	YIL096C		YIL096C Protein of unknown function
**98**	YIL127C		YIL127C; YI8277.02 Protein of unknown function
**99**	YIR012W	SQT1	SQT1; YIB12; YIB12W; YIR012W Protein that may be required for ribosomal assembly has WD (WD-40) repeat motifs
**100**	YIR026C	YVH1	YVH1; YIR026C Protein-tyrosine phosphatase (PTPase) with similarity to vaccinia VH1
**101**	YJL010C		YJL010C; J1357 Protein of unknown function
**102**	YJL033W	HCA4	HCA4; DBP4; UF1; J1250; YJL033W Probable RNA helicase CA4, involved in ribosomal RNA processing
**103**	YJL050W	MTR4	MTR4; DOB1; J1158; YJL050W Protein required for mRNA export from nucleus, member of the DEAD-box RNA helicase family
**104**	YJL069C		YJL069C; HRE594; J1098 Essential protein of unknown function
**105**	YJL109C		YJL109C; J0808 Protein with weak similarity to Drs2p
**106**	YJL122W		YJL122W; J0723 Protein of unknown function
**107**	YJL125C	GCD14	GCD14; J0710; YJL125C Protein required for translational repression of GCN4 mRNA
**108**	YJL148W	RPA34	RPA34; CST21; J0637; YJL148W RNA polymerase I subunit, not shared with other RNA polymerases
**109**	YJL208C	NUC1	NUC1; HRE329; J0310; YJL208C Nuclease with both DNase and RNase activity, major nuclease of mitochondria
**110**	YJR002W	MPP10	MPP10; YJR83.5; J1411; YJR002W Component of U3 snoRNP, required for pre-18S rRNA processing

**111**	YJR041C		YJR041C; GTF1174; J1622 Protein with similarity to Podospora anserina NADH dehydrogenase chain 4
**112**	YJR063W	RPA12	RPA12; RRN4; J1747; YJR063W RNA polymerase I subunit A12.2
**113**	YJR070C		YJR070C; J1814 Protein of unknown function
**114**	YJR071W		YJR071W; J1818 Protein of unknown function
**115**	YKL009W		MRT4; YKL160; YKL009W Protein involved in mRNA turnover
**116**	YKL021C	MAK11	MAK11; YKL021C Protein essential for replication of M double-stranded RNA (dsRNA) virus member of the WD (WD-40) repeat family
**117**	YKL078W		JA2; DHR2; YKL408; YKL078W RNA helicase of DEAH box family, possible pre-mRNA splicing factor
**118**	YKL082C		YKL082C Protein of unknown function
**119**	YKL099C		YKL099C; YKL449 Protein of unknown function
**120**	YKL143W	LTV1	LTV1; YKL2; YKL143W Protein required for viability at low temperature
**121**	YKL172W		EBP2; YKL636; YKL172W Protein involved in pre-rRNA processing and ribosomal subunit assembly
**122**	YKL181W	PRS1	PRS1; PRPS1; (PPS1); YKL181W Phosphoribosylpyrophosphate synthetase, synthesizes phosphoribosylpyrophosphate (PRPP) from ribose-5'phosphate and ATP, involved in pseudohyphal growth
**123**	YKL191W	DPH2	DPH2; YKL191W Diptheria toxin resistance protein, required for diphthamide biosynthesis
**124**	YKR024C	DBP7	DBP7; YKR024C Protein involved in 60S ribosomal large subunit biogenesis, member of DEAD-box family of putative ATP-dependent RNAhelicases
**125**	YKR025W		RPC37; YKR025W RNA polymerase III, C37 subunit
**126**	YKR056W	RNC1	TRM2; RNC1; NUC2; (NUD1); YKR056W Uridine methyltransferase that catalyzes the formation of ribothymidine at position 54 in cytoplasmicand mitochondrial tRNA
**127**	YKR060W		YKR060W Protein of unknown function
**128**	YKR079C		YKR079C Protein of unknown function
**129**	YKR081C		YKR081C; YKR401 Protein of unknown function
**130**	YLL008W	DRS1	DRS1; L1345; YLL008W RNA helicase of the DEAD box family involved in rRNA biogenesis
**131**	YLL011W	SOF1	SOF1; L1339; YLL011W Protein associated with U3 small nucleolar RNA (snoRNA) and involved in 18S pre-rRNA maturation has 7 WD (WD-40)repeats
**132**	YLL034C		YLL034C; YL34; L0919 Protein with similarity to mammalian valosin-containing protein (VCP), member of the AAA family of ATPases
**133**	YLR002C		YLR002C; L1392 Protein of unknown function
**134**	YLR003C		YLR003C; L1510 Protein of unknown function
**135**	YLR009W		YLR009W; L1551 Possible ribosomal protein with similarity to ribosomal protein L24
**136**	YLR051C		YLR051C; L2128 Protein of unknown function
**137**	YLR074C		BUD20; L2337; YLR074C Protein of unknown function, has a single C2H2-type zinc finger
**138**	YLR129W	DIP2	DIP2; L9233.1; L3116; YLR129W Dom34p-interacting protein, has WD (WD-40) repeats
**139**	YLR175W	CBF5	CBF5; LUC8; (ART1); L9470.11; YLR175W Putative ribosomal RNA pseudouridine synthase, associated with H/ACA class small nucleolarRNAs

**140**	YLR186W		YLR186W; L9470.5 Protein of unknown function
**141**	YLR196W	PWP1	PWP1; L8167.10; YLR196W Periodic tryptophan protein, has WD (WD-40) repeats
**142**	YLR197W	SIK1	SIK1; NOP56; L8167.9; YLR197W Nucleolar protein component of box C/D snoRNPs, which are necessary for 2'-O-methylation of ribosomalRNAs
**143**	YLR198C		YLR198C Protein of unknown function
**144**	YLR221C		YLR221C; L8083.7 Protein of unknown function
**145**	YLR222C		YLR222C; CST29; L8083.8 Protein of unknown function, overproduction causes chromosome instability and increased mitotic recombination,contains WD (WD-40) repeats
**146**	YLR276C	DBP9	DBP9; L9328.3; YLR276C Protein with similarity to DEAD box RNA helicases
**147**	YLR336C	SGD1	SGD1; L8300.12; YLR336C Essential nuclear protein involved in the HOG pathway
**148**	YLR401C		YLR401C; L8084.19 Protein with weak similarity to R. capsulatus protein nifR3
**149**	YLR409C		YLR409C; L8084.22 Protein of unknown function, has WD (WD-40) repeats
**150**	YLR435W		YLR435W; L9753.3 Protein of unknown function, may be involved with protein synthesis
**151**	YLR449W		FPR4; L9324.3; YLR449W Nucleolar peptidylprolyl cis-trans isomerase (PPIase)
**152**	YML022W	APT1	APT1; YML022W Adenine phosphoribosyltransferase (APRT), may be a heterodimer with Apt2p
**153**	YML056C		IMD4; YM9958.06; YML056C Protein with similarity to inosine-5'-monophosphate dehydrogenase
**154**	YML080W		DUS1; YML080W tRNA dihydrouridine synthase
**155**	YML093W		YML093W Protein of unknown function
**156**	YMR014W		BUD22; YM9711.01; YMR014W Protein with possible role in bud site polarity, has possible coiled-coil domain
**157**	YMR049C		ERB1; YM9796.02; YMR049C Protein with possible role in ribosome biogenesis, has similarity to mouse Bop1 growth supressor, has one WD(WD-40) domain
**158**	YMR093W		YMR093W; YM9582.18 Protein of unknown function, has WD (WD-40) repeats
**159**	YMR128W	ECM16	ECM16; DHR1; YM9553.04; YMR128W Putative DEAH-box RNA helicase, directly implicated in ribosome biogenesis
**160**	YMR131C		RSA2; YM9553.07; YMR131C Protein involved in ribosome assembly, member of WD (WD-40) repeat family
**161**	YMR217W	GUA1	GUA1; YM8261.11; YMR217W GMP synthetase, catalyzes the amination of xanthine monophosphate to guanine monophosphate in the guaninebranch of the purine biosynthesis pathway
**162**	YMR229C	FMI1	RRP5; FMI1; YM9959.11; YMR229C Protein required for processing of pre-rRNA to 18S and 5.8S rRNA
**163**	YMR239C	RNT1	RNT1; YM9959.21; YM9408.01; YMR239C Double-strand-specific ribonuclease required for rRNA processing, homolog of E. coli RNase III
**164**	YMR290C	HAS1	HAS1; YMR290C RNA helicase of the DEAD/DEAH box family
**165**	YMR310C		YMR310C; YM9924.02 Protein of unknown function
**166**	YNL002C	RLP7	RLP7; N2014; YNL002C Protein with similarity to ribosomal proteins including Rpl7p (Rpl7Ap and Rpl7Bp), involved in processing of precursorrRNAs gene has low codon bias
**167**	YNL022C		YNL022C; N2815 Protein of unknown function

**168**	YNL061W	NOP2	NOP2; YNA1; N2428; YNL2428; YNL061W Nucleolar protein, has strong similarity to human proliferation-associated p120 nucleolar antigen
**169**	YNL062C	GCD10	GCD10; YNL1616; YNL2422; N2422; YNL062C RNA-binding subunit of initiation factor eIF3
**170**	YNL075W		IMP4; N2353; YNL075W Component of U3 snoRNP, required for pre-18S rRNA processing
**171**	YNL110C		YNL110C; N1954 Protein of unknown function, contains one RNA recognition (RRM) domain
**172**	YNL112W	DBP2	DBP2; N1945; YNL112W ATP-dependent RNA helicase of DEAD box family
**173**	YNL113W	RPC19	RPC19; N1937; YNL113W Shared subunit of RNA polymerases I and III
**174**	YNL114C		YNL114C; N1934 Protein of unknown function, questionable ORF
**175**	YNL119W		YNL119W; N1913 Protein possibly involved in cytoplasmic ribosome function
**176**	YNL132W		KRE33; N1216; N1858; YNL132W Protein of unknown function
**177**	YNL141W	AAH1	AAH1; N1208; N1825; YNL141W Adenine deaminase (adenine aminohydrolase), enzyme of the purine salvage pathway has similarity toadenosine deaminases
**178**	YNL151C	RPC31	RPC31; RPC8; ACP2; N1769; YNL151C RNA polymerase III, small subunit, essential subunit, not shared
**179**	YNL174W		YNL174W; N1669 Protein of unknown function
**180**	YNL175C		NOP13; N1665; YNL175C Nucleolar protein with similarity to Nsr1p, has two RNA recognition (RRM) domains
**181**	YNL182C		YNL182C; N1636 Protein of unknown function
**182**	YNL248C	RPA49	RPA49; N0880; YNL248C RNA polymerase I third largest subunit
**183**	YNL299W	TRF5	TRF5; YNL0440; N0440; YNL299W DNA polymerase kappa, required for sister chromatid cohesion
**184**	YNL308C		KRI1; N0388; YNL308C Protein required for synthesis of 40S ribosomal subunits, binds Krr1p and has weak similarity to Mgm1p
**185**	YNL313C		YNL313C; N0364 Protein of unknown function, has tetratricopeptide (TPR) repeats
**186**	YNR012W	URK1	URK1; N2050; YNR012W Uridine kinase, converts ATP and uridine to ADP and UMP
**187**	YNR053C		YNR053C; N3484 Nuclear protein of unknown function, has similarity to human breast tumor-associated autoantigen
**188**	YNR054C		YNR054C; ABT1; N3491 Protein with similarity to mouse ABT1, an activator of RNA Polymerase II basal transcription
**189**	YOL010W		RCL1; O2357; YOL010W Protein required for pre-rRNA processing at cleavage sites A0, A1 and A2, has similarity to RNA 3'-terminal phosphatecyclase
**190**	YOL022C		YOL022C; O2189 Protein of unknown function
**191**	YOL041C		NOP12; O2049; YOL041C Protein of unknown function
**192**	YOL077C		BRX1; O1115; YOL077C Protein required for biogenesis of the 60S ribosomal subunit, localized to the nucleolus
**193**	YOL080C		REX4; O1101; YOL080C Member of the 3' -to 5' exonuclease family, has similarity to Rnh70p and Pan2p
**194**	YOL124C		YOL124C; O0546 Protein of unknown function
**195**	YOL144W		YOL144W; NOP8; AOB484; O0463 Nucleolar protein required for 60S ribosome biogenesis
**196**	YOR001W	RRP6	RRP6; UNC733; O2509; YOR001W Protein involved in 5.8S ribosomal RNA 3' end processing, involved in degradation of aberrant poly(A)-mRNAs in the nucleus, component of the nuclear form of the exosome
**197**	YOR004W		YOR004W; UNB254; O2521 Protein of unknown function
**198**	YOR056C		YOR056C; NOB1; YOR29-07; O280 Protein that associates with the 26S proteasome

**199**	YOR091W		YOR091W; YOR3160; O3160 Protein of unknown function
**200**	YOR095C	RKI1	RKI1; YOR3174; O3174; YOR095C Ribose 5-phosphate ketol-isomerase
**201**	YOR145C		YOR145C; O3513 Protein of unknown function
**202**	YOR206W		YOR206W; O4843; YOX001 Protein of unknown function
**203**	YOR224C	RPB8	RPB8; YOR50-14; O5014; O5070; YOR224C Shared subunit of RNA polymerases I, II, and III (ABC14.5)
**204**	YOR243C		YOR243C; O5254 Protein of unknown function
**205**	YOR252W		YOR252W; O5310 Protein of unknown function
**206**	YOR272W	YTM1	YTM1; CST14; O5437W; YOR272W Microtubule-associated protein essential for the G1/S transition, member of WD (WD-40) repeat family
**207**	YOR287C		YOR287C; O5492C Protein of unknown function, possible coiled-coil protein
**208**	YOR294W		RRS1; O5617; YOR294W Regulator of ribosome synthesis
**209**	YOR309C		YOR309C; O6105 Protein of unknown function
**210**	YOR310C	NOP5	NOP5; NOP58; LUC9; O6108; YOR310C Nucleolar protein component of box C/D snoRNPs involved in 2'-O-methylation of ribosomal RNAs
**211**	YOR340C	RPA43	RPA43; O6271; YOR340C RNA polymerase I subunit not shared with other polymerases
**212**	YPL012W		YPL012W; YP8132.01; LPA5 Protein of unknown function
**213**	YPL043W	NOP4	NOP4; NOP77; P7102.07; YPL043W Nucleolar protein required for ribosome biogenesis, has 3 canonical RNA recognition (RRM) domains andone degenerate RNA recognition (RRM) domain
**214**	YPL044C		YPL044C Protein of unknown function
**215**	YPL086C		ELP3; HPA1; LPG22; YPL086C Subunit of the elongator/RNAPII holoenzyme, has histone acetyltransferase activity
**216**	YPL093W		NOG1; LPG15; YPL093W Putative essential nucleolar GTP-binding protein, has similarity to Halobacterium cutirubrum GTP-binding protein(SP:P17103)
**217**	YPL126W		NAN1; LPH1; YPL126W Nucleolar protein associated with Net1p
**218**	YPL146C		YPL146C; P2610 Protein of unknown function
**219**	YPL183C		YPL183C; P2231 Protein of unknown function, has WD (WD-40) repeats
**220**	YPL207W		YPL207W; P1835 Protein of unknown function
**221**	YPL211W	NIP7	NIP7; P1810; YPL211W Nucleolar protein required for efficient 60S ribosome subunit biogenesis
**222**	YPL217C		BMS1; P1760; YPL217C Protein involved in bud site selection in diploids interacts genetically with BMH1
**223**	YPL226W		NEW1; P1445; YPL226W Member of the non-transporter group of the ATP-binding cassette (ABC) superfamily
**224**	YPL266W	DIM1	DIM1; P0367; YPL266W Dimethyladenosine transferase, (rRNA (adenine-N6,N6-)-dimethyltransferase), responsible for m6[2]Am6[2]Adimethylation in 3'-terminal loop of 18S rRNA
**225**	YPR110C	RPC40	RPC40; RPC5; P8283.18; YPR110C Shared subunit of RNA polymerases I and III
**226**	YPR112C		MRD1; P8283.19; YPR112C Protein with similarity to Pab1p, Pub1p, Nsr1p, Nop4p and other RNA-binding proteins, contains four RNArecognition (RRM) domains
**227**	YPR137W		RRP9; P9659.6; YPR137W Protein associated with the U3 small nucleolar RNA, required for pre-ribosomal RNA processing

**228**	YPR143W		YPR143W; P9659.4 Protein of unknown function
**229**	YPR144C		YPR144C; P9659.17 Protein of unknown function, has strong similarity to an S. pombe protein of unknown function
**230**	YPR163C	TIF3	TIF3; (STM1); RBL3; IF4B; P9325.6; YPR163C Translation initiation factor eIF4B, contains one RRM (RNA recognition motif) RNA-bindingdomain
**231**	YPR190C	RPC82	RPC82; RPC3; P9677.11; YPR190C RNA polymerase III, third-largest subunit

**Ribosomal proteins genes**			
**Serial #**	**ORF**	**Name**	**description**
**1**	YBL027W	RPL19A	RPL19B; YBL0424; YBL027W Ribosomal protein L19 (yeast L23; YL14; rp15L; rp33; rat L19), nearly identical to Rpl19Ap
**2**	YBL072C	RPS8A	RPS8A; (RPS14A); (RP19); YBL06.05; YBL0613; YBL072C Ribosomal protein S8 (yeast S14; YS9; rp19; mammalian S8), identical to Rps8Bp
**3**	YBL087C	RPL17A	RPL23A; YBL0713; YBL087C Ribosomal protein L23 (yeast L17a; YL32; E. coli L14; rat L23), nearly identical to Rpl23Bp
**4**	YBR048W	RPS18B	RPS11B; YBR0501; YBR048W Ribosomal protein S11 (yeast S18, YS12, rp41, E. coli S17, human and rat S11), identical to Rps11Bp
**5**	YBR181C	RPS10A	RPS6B; (RPS10A); RPS101; YBR1244; YBR181C Ribosomal protein S6 (yeast S10; YS4; rp9; human, mouse, and rat S6), identical to Rps6Ap
**6**	YBR191W	URP1	RPL21A; URP1A; URP1; YBR1401; YBR191W Ribosomal protein L21 (rat L21), nearly identical to Rpl21Bp
**7**	YCR031C	CRY1	RPS14A; CRY1; (RP59); YCR59; YCR031C Ribosomal protein S14A (rp59, E. coli S11, rat and human S14) involved in cryptopleurine resistance,nearly identical to Rps14Bp
**8**	YDL061C	YS29B	RPS29B; D2542; YDL061C Ribosomal protein S29 (yeast S36; YS29; rat S29), similar to Rps29Ap
**9**	YDL075W	RPL43A	RPL31A; D2478; YDL075W Ribosomal protein L31 (yeast L34; YL36; YL28; rat L31), nearly identical to Rpl31Bp
**10**	YDL081C	RPLA1	RPP1A; RPLA1; (RPA1); YP1ALPHA; L12eIIA; D2450; YDL081C Acidic ribosomal protein P1A (A1 YP1alpha; E. coli L12eIIA; human and ratP1)
**11**	YDL082W		RPL13A; D2445; YDL082W Ribosomal protein L13 (rat L13), nearly identical to Rpl13Bp
**12**	YDL083C		RPS16B; D2442; YDL083C Ribosomal protein S16 (rp61R rat S16), identical to Rps16Ap
**13**	YDL136W	SOS2	RPL35B; SOS2; D2170; YDL136W Ribosomal protein L35 (rat L35), identical to Rpl35Ap
**14**	YDL191W	SOS1	RPL35A; SOS1; D1249; YDL191W Ribosomal protein L35 (rat L35), identical to Rpl35Bp
**15**	YDR025W	RPS18A	RPS11A; PZA156; D9813.03; D3278; YDR025W Ribosomal protein S11 (yeast S18; YS12; rp41; E. coli S17; human and rat S11), identical toRps11Bp
**16**	YDR064W	RPS13C	RPS13; YS15; YD9609.18; D4252; YDR064W Ribosomal protein S13 (yeast S27; YS15; rat S13)
**17**	YDR382W	RPL45	RPP2B; RPLA4; RPL45; L12eIA; L36; YPA1; D9481.1; D9509.2; YDR382W Acidic ribosomal protein P2B (L45 YPA1; YL44C; YP2 beta E. coliL12eIA; human and rat P2)
**18**	YDR418W	RPL15A	RPL12B; D9461.7; YDR418W Ribosomal protein L12 (yeast L15; YL23; E. coli L11; rat L12a), identical to Rpl12Ap

**19**	YDR447C	RP51B	RPS17B; RP51B; D9461.32; YDR447C Ribosomal protein S17 (rp51 rat S17), nearly identical to Rpl17Ap
**20**	YDR450W		RPS18A; YDR450W; RPS13C; D9461.35 Ribosomal protein S18 (E. coli S13; rat S18), identical to Rps18Bp
**21**	YDR471W	RPL27B	RPL27B; D8035.14; YDR471W Ribosomal protein L27 (yeast L27; mammalian L27), identical to Rpl27Ap
**22**	YDR500C	RPL35B	RPL37B; D9719.6; YDR500C Ribosomal protein L37 (yeast L46) (rat L37), nearly identical to Rpl37Bp
**23**	YEL054C	RPL15B	RPL12A; YEL054C Ribosomal protein L12 (yeast L15; YL23; E. coli L11; rat L12b), identical to Rpl12Bp
**24**	YER074W	RPS24EA	RPS24A; YER074W Ribosomal protein S24 (rat S24), identical to Rps24Bp
**25**	YER102W	RPS8B	RPS8B; (RPS14B); (RP19); YER102W Ribosomal protein S8 (yeast S14; YS9; rp19; mammalian S8), identical to Rps8Ap
**26**	YER117W	RPL17B	RPL23B; YER117W Ribosomal protein L23 (yeast L17a; YL32; E. coli L14; rat L23), nearly identical to Rpl23Ap
**27**	YER131W	RPS26B	RPS26B; YER131W Ribosomal protein S26 (rat S26), nearly identical to Rps26Ap
**28**	YGL030W	RPL32	RPL30; (RPL32); G3652; YGL030W Large-subunit ribosomal protein L30 (yeast L32; YL38; rp73; mouse and rat L30)
**29**	YGL031C	RPL30A	RPL24A; G3648; YGL031C Ribosomal protein L24A (yeast L30; rp29; YL21; rat L24), nearly identical to Rpl24Bp
**30**	YGL076C	RPL6A	RPL7A; YL8A; G3216; YGL076C Ribosomal protein L7 (yeast L6; YL8A; rp11; E. coli L30; rat L7), nearly identical to Rpl7Bp
**31**	YGL103W	CYH2	RPL28; CYH2; G3101; YGL103W Ribosomal protein L28 (yeast L29; YL24; rp44; mouse and rat L27a)
**32**	YGL123W	SUP44	RPS2; (RPS4); SUP44; G2893; YGL123W Ribosomal protein S2 (yeast S4; YS5; YP9; rp12; E. coli S5; rat S2)
**33**	YGL135W	SSM2	RPL1B; SSM1B; SSM2; G2834; YGL135W Large subunit ribosomal protein L1 (rat L10A; eubacterial L1; archeal L1), identical to Rpl1Ap
**34**	YGL147C	RPL9A	RPL9A; RP25; YL11; G2505; YGL147C Ribosomal protein L9 (YL11 rp25; rp24; E. coli L6; rat L9), nearly identical to Rpl9Bp
**35**	YGR027C	RPS31A	RPS25A; RPS31A; G4046; YGR027C Ribosomal protein S25A (yeast S31; YS23; rp45; rat S25), identical to Rps25Bp
**36**	YGR034W		RPL26B; G4079; YGR034W Ribosomal protein L26 (yeast L33; YL33; rat L26), nearly identical to Rpl26Ap
**37**	YGR085C	RPL16A	RPL11B; G4620; YGR085C Ribosomal protein L11 (yeast L16; YL22; rp39B; E. coli L5; rat L11), nearly identical to Rpl11Ap
**38**	YGR118W	RPS28A	RPS23A; (RPS28A); G6178; YGR118W Ribosomal protein S23 (yeast S28; rp37; YS14; E. coli S12; rat S23), identical to Rps23Bp
**39**	YGR148C	RPL30B	RPL24B; G6635; YGR148C Ribosomal protein L24B (yeast L30; rp29; YL21; rat L24), nearly identical to Rpl24Ap
**40**	YGR214W	YST1	RPS0A; YST1; (NAB1); NAB1A; G7816; YGR214W Ribosomal protein S0 (E. coli S2; rat Sa), nearly identical to Rps0Bp
**41**	YHL001W	RPL14B	RPL14B; YHL001W Ribosomal protein L14 (mammalian L14), nearly identical to Rpl14Ap
**42**	YHL015W	URP2	RPS20; URP2; YHL015W Ribosomal protein S20 (E. coli S10; Xenopus S22; rat and human S20)
**43**	YHL033C	RPL4A	RPL8A; MAK7; YHL033C Ribosomal protein L8 (yeast L4; rp6; YL5; human, mouse, and rat L7a), involved in maintenance of M1 dsRNA virus;nearly identical to Rpl8Bp
**44**	YHR010W	RPL27	RPL27A; YHR010W Ribosomal protein L27 (yeast L27; mammalian L27), identical to Rpl27Bp

**45**	YHR021C	RPS27B	RPS27B; SLO81; YHR021C Ribosomal protein S27 (yeast S27; rp61; YS20; mammalian S27), nearly identical to Rps27Ap
**46**	YHR141C	MAK18	RPL42B; MAK18; SCL41B; YHR141C Ribosomal protein L42 (yeast L41; YL27; YP44; human and rat L36A), involved in replication of M1 and M2double-stranded RNA (dsRNA) viruses, identical to Rpl42Ap
**47**	YHR203C	RPS7A	RPS4B; (RPS4); (RPS7A); YHR203C Ribosomal protein S4 (yeast S7; YS6; rp5; rat and human S4), identical to Rps4Ap
**48**	YIL018W	RPL5A	RPL2B; RPL5A; YL6b; YI3299.13; YIL018W Ribosomal protein L2 (yeast L5; YL6; rp8; E. coli L2; tobacco L2; rat L8)
**49**	YIL052C		RPL34B; YIL052C Ribosomal protein L34 (rat L34), nearly identical to Rpl34Ap
**50**	YIL133C	RPL13	RPL16A; YIL133C Ribosomal protein L16 (rp22), nearly identical to Rpl16Bp
**51**	YIL148W	UBI1	RPL40A; UBI1; CEP52A; CST2; YIL148W Fusion protein whose N-terminal half is ubiquitin and whose C-terminal half is ribosomal protein L40(rat L40), identical to Rpl40Bp
**52**	YJL136C	RPS25B	RPS21B; (RPS26B); J0664; YJL136C Ribosomal protein S21 (yeast S26; YS25; rat S21), identical to Rps21Ap
**53**	YJL138C	TIF2	TIF2; J0660; YJL138C Translation initiation factor 4A (eIF4A) of the DEAD box family, identical to Tif1p
**54**	YJL177W		RPL17B; J0493; YJL177W Ribosomal protein L17 (yeast L20; YL17; E. coli L22; rat and human L17), nearly identical to Rpl17Ap
**55**	YJL190C	RPS24A	RPS22A; (RPS24); J0355; YJL190C Ribosomal protein S22 (yeast S24; rp50; YS22; rat S15A), nearly identical to Rps22Bp
**56**	YJR123W	RPS5	RPS5; J2045; YJR123W Ribosomal protein Rps5p (rp14 YS8; S2; mammalian S5) of the small subunit
**57**	YJR145C	RPS7B	RPS4A; (RPS4); (RPS7B); J2186; YJR145C Ribosomal protein S4 (yeast S7; YS6; rp5; rat and human S4), identical to Rps4Bp
**58**	YKL006W	RPL14A	RPL14A; YKL153; YKL006W Ribosomal protein L14 (mammalian L14), nearly identical to Rpl14Bp
**59**	YKL056C		YKL056C; YKL312 Protein possibly involved in cytoplasmic ribosome function, has similarity to translationally controlled tumor protein (TCTP) ofanimal cells and higher plants
**60**	YKL156W	RPS27A	RPS27A; YKL156W Ribosomal protein S27 (yeast S27; rp61; YS20; mammalian S27), nearly identical to Rps27Bp
**61**	YKL180W	RPL17	RPL17A; YKL180W Ribosomal protein L17 (yeast L20; YL17; E. coli L22; rat and human L17), nearly identical to Rpl17Bp
**62**	YKR057W	RPS25	RPS21A; (RPS26A); YKR057W Ribosomal protein S21 (yeast S26; YS25; rat S21), identical to Rps21Bp
**63**	YKR059W	TIF1	TIF1; YKR059W Translation initiation factor 4A (eIF4A) of the DEAD box family, identical to Tif2p
**64**	YKR094C	UBI2	RPL40B; UBI2; CEP52B; CST1; YKR414; YKR094C Fusion protein comprised of ribosomal protein L40 (C-terminal half) and ubiquitin(N-terminal half), (rat L40), identical to Rpl40Ap
**65**	YLL045C	RPL4B	RPL8B; KRB1; L0717; YLL045C Ribosomal protein L8 (yeast L4; rp6; YL5; human, rat, and mouse L7a), involved in maintenance of M1 dsRNAvirus nearly identical to Rpl8Ap
**66**	YLR029C	RPL13A	RPL15A; L1755; YLR029C Ribosomal protein L15 (yeast L13; YL10; rp15R; rat L15), nearly identical to Rpl15Bp
**67**	YLR048W	YST2	RPS0B; YST2; NAB1B; L2118; YLR048W Ribosomal protein S0 (E. coli S2; rat Sb), nearly identical to Rps0Ap
**68**	YLR061W		RPL22A; YL31; L2168; YLR061W Ribosomal protein L22, similar to Rpl22Bp
**69**	YLR075W	GRC5	RPL10; GRC5; QSR1; L2341; YLR075W Ribosomal protein L10 (yeast L9)

**70**	YLR167W	UBI3	RPS31; UBI3; L9470.14; YLR167W Fusion protein comprised of ribosomal protein S31 at the C-terminal half fused to ubiquitin at the N-terminalhalf (yeast S37; YS24; rat S27a)
**71**	YLR185W	RPL35A	RPL37A; L9470.6; YLR185W Ribosomal protein L37 (yeast L46; rat L37), nearly identical to Rpl37Bp
**72**	YLR264W	RPS33B	RPS28B; L8479.5; YLR264W Ribosomal protein S28 (yeast S33; YS27; mammalian S28), nearly identical to Rps28Ap
**73**	YLR325C		RPL38; L8543.2; YLR325C Ribosomal protein L38
**74**	YLR333C	RPS31	RPS25B; RPS31B; L8300.10; YLR333C Ribosomal protein S25B (yeast S31; YS23; rp45; rat S25), identical to Rps25Ap
**75**	YLR372W	SUR4	SUR4; (APA1); VBM1; ELO3; SRE1; L8039.2; YLR372W Protein required for the conversion of 24-carbon fatty acids to 26-carbon fatty acids
**76**	YLR388W		RPS29A; L8084.11; YLR388W Ribosomal protein S29 (yeast S36; YS29; rat S29), similar to Rps29Bp
**77**	YLR441C	RP10A	RPS1A; RP10A; (PLC1); L9753.9; YLR441C Ribosomal protein S1 (rp10 rat S3A), nearly identical to Rps1Bp
**78**	YLR448W	RPL16B	RPL6B; L9324.4; YLR448W Ribosomal protein L6 (yeast L17B; YL16B; human L6), nearly identical to Rpl6Ap
**79**	YML024W		RPS17A; RP51A; YML024W Ribosomal protein S17 (rp51 rat S17), nearly identical to Rps17Bp
**80**	YML026C		RPS18B; YML026C Ribosomal protein S18 (E. coli S13; rat S18), identical to Rps18Ap
**81**	YML063W	RP10B	RPS1B; RP10B; RP3B; PLC2; KRP-Y1; YML063W Ribosomal protein S1 (rp10 rat S3A), nearly identical to Rps1Ap
**82**	YML073C	YL16A	RPL6A; YML073C Ribosomal protein L6 (yeast L17A; YL16A; human L6), nearly identical to Rpl6Bp
**83**	YMR116C	BEL1	ASC1; BEL1; CPC2; YM9718.15; YMR116C Ribosomal protein of the 40S ribosomal subunit that influences translational efficiency and cell size,contains two WD (WD-40) repeats
**84**	YMR142C		RPL13B; YM9375.11; YMR142C Ribosomal protein L13 (rat L13), nearly identical to Rpl13Ap
**85**	YMR194W		RPL36A; YM9646.06; YMR194W Ribosomal protein L36 (yeast L39; YL39; rat L36), nearly identical to Rpl36Bp
**86**	YMR217W	GUA1	GUA1; YM8261.11; YMR217W GMP synthetase, catalyzes the amination of xanthine monophosphate to guanine monophosphate in the guaninebranch of the purine biosynthesis pathway
**87**	YMR242C		RPL20A; YM9408.04; YMR242C Ribosomal protein L20, nearly identical to Rpl20Bp
**88**	YNL067W	RPL9B	RPL9B; N2406; YNL1611; YNL2406; YNL067W Ribosomal protein L9 (YL11 rp25; rp24; E. coli L6; rat L9), nearly identical to Rpl9Ap
**89**	YNL069C	RP23	RPL16B; N2377; YNL069C Ribosomal protein L16 (rp23), nearly identical to Rpl16Ap
**90**	YNL096C		RPS7B; N2212; YNL096C Ribosomal protein S7 (rp30 human S7; Xenopus S8), nearly identical to Rps7Ap
**91**	YNL162W	RPL41A	RPL42A; SCL41A; N1722; YNL162W Ribosomal protein L42 (yeast L41; YL27; YP44; human and rat L36A), identical to Rpl42Bp
**92**	YNL178W	RPS3	RPS3; RP13; SUF14; N1653; YNL178W Ribosomal protein S3 (rp13 YS3; mammalian S3), has a possible KH domain
**93**	YNL209W	SSB2	SSB2; N1333; YNL209W Heat shock protein of HSP70 family, cytoplasmic
**94**	YNL301C	RP28B	RPL18B; RP28B; N0425; YNL0425; YNL301C Ribosomal protein L18 (rp28 rat L18), identical to Rpl18Ap
**95**	YNL302C	RPS16A	RPS19B; RP55B; (RPS16B); N0422; YNL0422; YNL302C Ribosomal protein S19 (rp55 YS16B; rat S19), nearly identical to Rps19Ap

**96**	YOL039W	RPLA2	RPP2A; RPLA2; RPL44; (RPA2); L12eIB; L35; O2060; YOL039W Acidic ribosomal protein P2A (L44 A2; YP2alpha; E. coli L12eIB; humanP2alpha), plays a role in the elongation step
**97**	YOL040C	RPS21	RPS15; RPS21; O2057; YOL040C Ribosomal protein S15 (yeast S21; rp52; E. coli S19; rat S15; RIG protein)
**98**	YOL120C	RP28A	RPL18A; RP28A; O0565; YOL120C Ribosomal protein L18 (rp28 rat L18), identical to Rpl18Bp
**99**	YOL121C	RPS16B	RPS19A; RP55A; (RPS16A); O0559; YOL121C Ribosomal protein S19 (rp55 YS16A; rat S19), nearly identical to Rps19Bp
**100**	YOL127W	RPL25	RPL25; O0534; YOL127W Ribosomal protein L25 (YL25 rp61L; E. coli L23; rat L23a)
**101**	YOR063W	TCM1	RPL3; TCM1; MAK8; O2812; YOR29-14; YOR063W Ribosomal protein L3 (YL1 rp1; rat L3) , responsible for trichodermin resistance andinvolved in maintenance of dsRNA viruses
**102**	YOR096W	RPS30	RPS7A; RP30; YOR3177; O3177; YOR096W Ribosomal protein S7 (rp30 human S7; Xenopus S8), nearly identical to Rps7Bp
**103**	YOR167C	RPS33A	RPS28A; O3598; YOR167C Ribosomal protein S28 (yeast S33; YS27; mammalian S28), nearly identical to Rps28Bp
**104**	YOR234C	RPL37B	RPL33B; O5224; YOR234C Ribosomal protein L33 (yeast L37; YL37; rp47; rat L35A), nearly identical to Rpl33Ap
**105**	YOR293W		RPS10A; O5611; YOR293W Ribosomal protein S10 (rat S10), nearly identical to Rps10Bp
**106**	YOR312C	RPL18A1	RPL20B; O6116; YOR312C Ribosomal protein L20, nearly identical to Rpl20Ap
**107**	YOR369C	RPS12	RPS12; RS12; O6673; YOR369C Ribosomal protein S12 (rat S12)
**108**	YPL037C	EGD1	EGD1; CST25; P7102.13; YPL037C Beta subunit of the nascent polypeptide-associated complex
**109**	YPL079W		RPL21B; URP1B; LPF6; YPL079W Ribosomal protein L21 (rat L21), nearly identical to Rpl21Ap
**110**	YPL090C		RPS6A; (RPS10B); RPS102; LPG18; YPL090C Ribosomal protein S6 (yeast S10; YS4; rp9; human, mouse, and rat S6), identical to Rps6Bp
**111**	YPL143W	RPL37A	RPL33A; LPI4; P2625; YPL143W Ribosomal protein L33 (yeast L37; YL37; rp47; rat L35A), nearly identical to Rpl33Bp
**112**	YPL220W	SSM1	RPL1A; SSM1A; SSM1; P1740; YPL220W Large subunit ribosomal protein L1 (rat L10A; eubacterial L1; archeal L1), identical to Rpl1Bp
**113**	YPR102C		RPL11A; P8283.14; YPR102C Ribosomal protein L11 (yeast L16; YL22; rp39A; E. coli L5; rat L11), nearly identical to Rpl11Bp
**114**	YPR132W	RPS28B	RPS23B; (RPS28B); P9659.9; YPR132W Ribosomal protein S23 (yeast S28; rp37; YS14; E. coli S12; rat S23), identical to Rps23Ap
**115**	YBR084CA		RPL19A; YBR084C-A Ribosomal protein L19 (yeast L23; YL14; rp15L; rp33; rat L19), nearly identical to Rpl19Bp
**116**	YER056CA		RPL34A; YER056C-A Ribosomal protein L34 (rat L34), nearly identical to Rpl34Bp
**117**	YFR031CA		RPL2A; YL6; YFR031C-A Ribosomal protein L2 (Yeast L5; YL6; rp8; E. coli L2; tobacco L2; rat L8)

**Cell-cycle G1 genes**			
**Serial #**	**ORF**	**Name**	**description**

**1**	YAL007C		ERP2; (FUN54); YAL007C Component of COPII-coated vesicles, forms an Erp1p-Erp2p-Emp24p-Erv25p heteromeric complex, has similarity toEmp24p and Erv25p
**2**	YAR003W		YAR003W; FUN16 Protein of unknown function, contains a WD (WD-40) repeat
**3**	YAR007C	RFA1	RFA1; BUF2; (RPA1); FUN3; SRR1; YAR007C DNA replication factor A, 69K subunit, binds single-stranded DNA
**4**	YAR008W	SEN34	SEN34; FUN4; YAR008W tRNA splicing endonuclease, gamma subunit, has active site for 3' splice site cleavage
**5**	YBL002W	HTB2	HTB2; H2B2; YBL0104; YBL002W Histone H2B, nearly identical to Htb1p
**6**	YBL003C	HTA2	HTA2; H2A2; YBL0103; YBL003C Histone H2A, nearly identical to Hta1p
**7**	YBL009W		YBL009W; YBL0317 Protein of unknown function
**8**	YBL010C		YBL010C; YBL0316 Protein of unknown function
**9**	YBL020W	RFT1	RFT1; PER5; YBL0442; YBL020W Protein involved required for N-linked glycosylation
**10**	YBL031W	SHE1	SHE1; YBL0420; YBL031W Protein that causes lethality when overexpressed
**11**	YBL032W		YBL032W; YBL0418 Protein with four KH RNA-binding domains, has similarity to mouse hnRNP X protein
**12**	YBL035C	POL12	POL12; YBL0414; YBL035C DNA polymerase alpha 86 kDa subunit, B subunit of polymerase alpha-primase complex
**13**	YBR009C	HHF1	HHF1; H4I; YBR0122; YBR009C Histone H4, identical to Hhf2p
**14**	YBR010W	HHT1	HHT1; H3I; (SIN2); BUR5; YBR0201; YBR010W Histone H3, identical to Hht2p
**15**	YBR015C	TTP1	MNN2; LDB8; TTP1; CRV4; ORE16; ORE19; YBR0220; YBR015C Mannosyltransferase required for forming and extending the mannosebranches of the outer chain mannans
**16**	YBR070C		YBR070C; (SAT2); YBR0711 Protein possibly involved in osmotolerance
**17**	YBR071W		YBR071W; YBR0712 Protein with weak similarity to Herpesvirus saimiri EERF2
**18**	YBR073W	RDH54	RDH54; TID1; YBR0715; YBR073W Protein required for mitotic diploid-specific recombination and repair and for meiosis
**19**	YBR078W	ECM33	ECM33; YBR0727; YBR078W Protein with predicted GPI-anchor, involved in cell wall structure or biosynthesis
**20**	YBR087W	RFC5	RFC5; YBR0810; YBR087W Replication factor C, 40 kDa subunit
**21**	YBR088C	POL30	POL30; PCNA YBR0811; YBR088C Proliferating cell nuclear antigen (PCNA), required for DNA synthesis and DNA repair
**22**	YBR089W		YBR089W; YBR0811a Protein of unknown function, questionable ORF
**23**	YBR106W	PHO88	PHO88; YBR0835; YBR106W Membrane protein involved in inorganic phosphate transport
**24**	YBR161W		YBR161W; YBR1212 Protein with similarity to Sur1p, Hoc1p, and Och1p
**25**	YBR184W	MEL1	YBR184W; YBR1306 Protein of unknown function
**26**	YBR187W		YBR187W; YBR1310 Protein of uknnon function
**27**	YBR233W		PBP2; YBR1531; YBR233W Protein that confers resistance to the antimalarial drug mefloquine when overproduced, contains KH RNA-bindingdomains
**28**	YBR243C	ALG7	ALG7; TUR1; YBR1628; YBR243C UDP-N-acetyl-glucosamine-1-P transferase (GPT) ER protein that transfers GlcNac-P from UDP-GlcNac toDol-P

**29**	YBR245C		ISW1; SGN2; YBR1633; YBR245C Putative ATP-dependent chromatin remodeling factor, has strong similarity to Drosophila nucleosomeremodeling factor ISWI
**30**	YBR252W	DUT1	DUT1; YBR1705; YBR252W dUTP pyrophosphatase, converts dUTP to dUMP thereby preventing uracil incorporation into DNA
**31**	YCL022C		YCL022C Protein of unknown function
**32**	YCL024W		KCC4; YCL024W Serine/threonine protein kinase involved in septin organization and cell cycle control
**33**	YCL061C		YCL061C; YCL060C Protein of unknown function
**34**	YCR034W	FEN1	FEN1; GNS1; VBM2; ELO2; SYR3; YCR521; YCR34; YCR034W Protein involved in the elongation of fatty acids up to 24 carbons
**35**	YCR065W	HCM1	HCM1; YCR902; YCR065W Dosage-dependent suppressor of cmd1 and member of the forkhead family of DNA-binding proteins
**36**	YDL003W	MCD1	MCD1; RHC21; (SCC1); PDS3; YD8119.04; D2940; YDL003W Cohesin, protein required for mitotic chromatid cohesion and chromosomecondensation
**37**	YDL011C		YDL011C; D2885 Protein of unknown function
**38**	YDL018C		ERP3; D2850; YDL018C Protein with similarity to COPII-coated vesicle proteins Emp24p and Erv25p
**39**	YDL055C	PSA1	PSA1; MSN17; SRB1; VIG9; MPG1; D2566; YDL055C Mannose-1-phosphate guanyltransferase GDP-mannose pyrophosphorylase
**40**	YDL093W	PMT5	PMT5; D2399; YDL093W Protein with similarity to O-mannosyltransferases Pmt1p, Pmt2p, Pmt3p, Pmt4p, and Pmt6p
**41**	YDL094C		YDL094C; D2395 Protein of unknown function
**42**	YDL101C	DUN1	DUN1; D2370; YDL101C Protein kinase required for induction of Rnr3p and DNA repair genes after DNA damage
**43**	YDL103C	QRI1	QRI1; UAP1; D2362; YDL103C UDP-N-acetylglucosamine pyrophosphorylase
**44**	YDL105W	QRI2	QRI2; D2354; YDL105W Protein of unknown function
**45**	YDL155W	CLB3	CLB3; D1539; YDL155W G2/M-phase-specific cyclin
**46**	YDL156W		YDL156W; D1536 Protein of unknown function has WD (WD-40) repeats
**47**	YDL157C		YDL157C; D1533 Protein of unknown function
**48**	YDL163W		YDL163W; D1505 Protein of unknown function
**49**	YDL164C	CDC9	CDC9; MMS8; D1497; YDL164C DNA ligase, functions in DNA replication and repair in both the nucleus and mitochondrion
**50**	YDL197C	ASF2	ASF2; D1219; YDL197C Anti-silencing protein that causes depression of silent loci when overexpressed
**51**	YDL211C		YDL211C; D1026 Protein of unknown function
**52**	YDL219W		YDL219W; DTD1; D0867 D-Tyr-tRNA[Tyr] deacylase
**53**	YDR013W		YDR013W; PZA208; D3225; YD8119.18 Protein of unknown function
**54**	YDR097C	MSH6	MSH6; PMS3; YD8557.04; YDR097C Component with Msh2p of DNA mismatch binding factor involved in repair of single base mismatches
**55**	YDR113C	PDS1	PDS1; YD9727.08; YDR113C Protein that regulates sister chromatid separation in mitosis

**56**	YDR144C	MKC7	MKC7; YPS2; YD8358.01; YD2943.03; YDR144C Aspartyl protease found in the periplasmic space, has similarity to Yps1p and Bar1p
**57**	YDR224C	HTB1	HTB1; H2B1; SPT12; YD9934.09; YDR224C Histone H2B
**58**	YDR225W	HTA1	HTA1; SPT11; H2A1; YD9934.10; YDR225W Histone H2A, nearly identical to Hta2p
**59**	YDR279W		YDR279W; D9954.2 Protein of unknown function
**60**	YDR297W	SUR2	SUR2; TSC7; SYR2; D9740.8; YDR297W Hydroxylase involved in sphingolipid metabolism
**61**	YDR302W		GPI11; D9740.5; YDR302W Protein involved in glycosylphosphatidylinositol (GPI) biosynthesis, with similarity to GPI-anchor biosynthesisprotein PIG-F
**62**	YDR307W		YDR307W; D9740.4 Protein with similarity to Pmt1p
**63**	YDR356W	NUF1	NUF1; SPC110; D9476.3; YDR356W Spindle pole body component with coiled-coil structure, determines the spacing between the ends ofmicrotubules and the central plaque
**64**	YDR400W		URH1; D9509.19; YDR400W Uridine ribohydrolase
**65**	YDR440W	PCH1	DOT1; PCH1; D9461.26; YDR440W Protein involved in silencing at telomeres and other repressed loci and meiotic arrest checkpoint
**66**	YDR451C		YHP1; D9461.36; YDR451C Protein with similarity to Yox1p, contains a homeodomain
**67**	YDR499W		LCD1; DDC2; CIP1; PIE1; D9719.5; YDR499W Essential protein required for DNA damage checkpoint pathway in G1 and G2/M phases of cellcycle
**68**	YDR501W		PLM2; D9719.7; YDR501W Protein required for plasmid maintenance
**69**	YDR507C	GIN4	GIN4; ERC47; CLA6; D9719.13; YDR507C Serine/threonine-protein kinase required for septin organization at the bud neck, has similarity toYcl024p
**70**	YDR528W		HLR1; D9719.31; YDR528W Protein of unknown function
**71**	YEL017W		YEL017W Protein of unknown function
**72**	YEL042W	GDA1	GDA1; SYGP-ORF16; YEL042W Guanosine diphosphatase of Golgi membrane
**73**	YEL061C	CIN8	CIN8; KSL2; SLN2; SDS15; CST16; YEL061C Kinesin-related protein involved in establishment and maintenance of mitotic spindle
**74**	YEL064C		YEL064C Putative membrane transporter of the amino acid/auxin permease (AAAP) family
**75**	YER001W	MNN1	MNN1; YER001W Alpha-1,3-mannosyltransferase, required for complex glycosylation of both N- and O-oligosaccharides
**76**	YER003C	PMI40	PMI40; YER003C Mannose-6-phosphate isomerase, generates mannose-6-phosphate for synthesis of GDP-mannose anddolichol-phosphate-mannose
**77**	YER016W	BIM1	BIM1; YER016W Protein associated with microtubules, required for a cell cycle check point
**78**	YER032W	FIR1	FIR1; PIP1; YER032W Protein probably involved in 3'-mRNA processing interacts with Pap1p and Ref2p,
**79**	YER070W	RNR1	RNR1; SDS12; CRT7; YER070W Ribonucleotide reductase (ribonucleoside-diphosphate reductase) large subunit, converts ribonucleosidediphosphate to deoxyribonucleoside diphosphate
**80**	YER095W	RAD51	RAD51; MUT5; YER095W Protein that stimulates pairing and strand-exchange between homologous single-stranded and double-stranded DNA,functionally similar to E. coli RecA protein

**81**	YER111C	SWI4	SWI4; (ART1); SLM4; YER111C Transcription factor that is a component of the SBF (Swi4p-Swi6p) complex that regulates expression atpromoters containing the cell cycle box (CCB) element, contains two ankyrin repeats
**82**	YER118C	SSU81	SHO1; SSU81; YER118C Osmosensor in the HOG1 MAP kinase, high-osmolarity signal transduction pathway, has an SH3 domain
**83**	YER149C	PEA2	PEA2; PPF2; DFG9; YER149C Protein involved in oriented growth toward mating partner
**84**	YER170W	ADK2	ADK2; ADK3; AKY3; PAK3; YER170W Adenylate kinase (GTP:AMP phosphotransferase), mitochondrial
**85**	YFL008W	SMC1	SMC1; CHL10; YFL008W Coiled-coil protein of the SMC family involved in chromosome condensation and segregation
**86**	YFL037W	TUB2	TUB2; SHE8; ARM10; YFL037W Tubulin beta chain, required for mitosis and karyogamy
**87**	YFL045C	SEC53	SEC53; ALG4; MCD5; YFL045C Phosphomannomutase, involved in the synthesis of GDP-mannose and dolichol-phosphate-mannose
**88**	YGL022W	STT3	STT3; G3683; RHK2; YGL022W Oligosaccharyltransferase subunit, member of a complex of eight ER proteins that transfers core oligosaccharidefrom dolichol carrier to Asn-X-Ser/Thr motif
**89**	YGL027C	CWH41	CWH41; G3664; YGL027C Glucosidase I, type II integral N-glycoprotein of the endoplasmic reticulum involved in beta-1,6-glucan assembly
**90**	YGL038C	OCH1	OCH1; NGD29; G3626; YGL038C Alpha-1,6-mannosyltransferase, involved in initiation of mannose outer chain elongation of N-linkedoligosaccharides of type Man[9]GlcNac[2]
**91**	YGL061C		DUO1; G3432; YGL061C Protein that forms a complex with Dam1p, required for maintenance of metaphase and anaphase spindle integrity
**92**	YGL065C	ALG2	ALG2; G3409; YGL065C Mannosyltransferase involved in N-glycosylation, converts Man[2]GlcNAc-PP-Dolichol to Man[3]GlcNAc-PP-Dolichol
**93**	YGL075C		MPS2; MMC1; G3219; YGL075C Protein of the nuclear envelope/endoplasmic reticulum required for spindle pole body assembly and normalchromosome segregation
**94**	YGL093W		SPC105; G3168; YGL093W Protein of the spindle pole body
**95**	YGL200C	EMP24	EMP24; BST2; G1271; YGL200C Component of COPII-coated vesicles possibly involved in cargo sorting
**96**	YGL207W	SPT16	SPT16; CDC68; (SSF1); G1160; YGL207W Essential nuclear protein required for gene activation and also chromatin-mediated repression,interacts with Pob3p to form the CP complex
**97**	YGL225W	GOG5	GOG5; VRG4; VAN2; MCD3; G1001; VIG4; YGL225W Golgi GDP-mannose transporter, member of the nucleotide-sugar transporter (NST) familyof membrane transporters
**98**	YGR014W	MSB2	MSB2; G4017; YGR014W Protein for which overproduction suppresses bud emergence defect of cdc24 mutant
**99**	YGR041W	BUD9	BUD9; G4152; YGR041W Protein required for bipolar budding mutant diploid strains bud only at distal pole
**100**	YGR099W	TEL2	TEL2; G5714; YGR099W Protein involved in controlling telomere length and telomere position effect
**101**	YGR109C	CLB6	CLB6; G5970; YGR109C B-type cyclin appearing late in G1, involved in initiation of DNA synthesis
**102**	YGR113W		DAM1; G6153; YGR113W; DIF1 Duo1p-interacting protein, required for maintenance of spindle integrity during mitosis

**103**	YGR140W	CBF2	CBF2; NDC10; CBF3A; CTF14; CEP2; (CSL5); G6425; YGR140W Component (subunit a) of Cbf3 kinetochore complex
**104**	YGR151C		YGR151C; G6655 Protein of unknown function
**105**	YGR152C	RSR1	RSR1; BUD1; G6658; YGR152C GTP-binding protein involved in bud site selection, member of the ras family in the ras superfamily
**106**	YGR172C	YIP1	YIP1; G7107; YGR172C Protein involved in vesicular transport interacts with transport GTPases Ypt1p and Ypt31p at the Golgi membrane
**107**	YGR188C	BUB1	BUB1; G7542; YGR188C Serine/threonine protein kinase and checkpoint protein required for cell cycle arrest in response to loss of microtubulefunction
**108**	YGR189C		CRH1; G7553; YGR189C Cell wall protein
**109**	YGR221C		TOS2; ORF622; G8523; YGR221C Protein of unknown function
**110**	YHL003C	LAG1	LAG1; YHL003C Protein required with Lac1p for ER-to-Golgi transport of GPI-anchored proteins, has indirect effect on cell longevity
**111**	YHR050W	SMF2	SMF2; YHR050W Probable manganese transporter
**112**	YHR061C	GIC1	GIC1; YHR061C Putative effector of Cdc42p, important for bud emergence
**113**	YHR110W		ERP5; YHR110W Protein with similarity to COPII-coated vesicle proteins Emp24p and Erv25p
**114**	YHR123W	EPT1	EPT1; YHR123W sn-1,2-Diacylglycerol ethanolaminephosphotransferase, catalyzes the synthesis of phosphatidylethanolamine fromCDP-ethanolamine and diacylglycerol
**115**	YHR127W		HSN1; YHR127W High-copy allele-specific suppressor SEC4
**116**	YHR149C		YHR149C Protein of unknown function
**117**	YHR153C	SPO16	SPO16; YHR153C Early meiotic protein required for efficient spore formation
**118**	YHR154W		ESC4; YHR154W Protein involved in chromatin silencing
**119**	YHR172W	SPC97	SPC97; YHR172W Spindle pole body component that plays a role in organization of nuclear and cytoplasmic microtubules
**120**	YHR173C		YHR173C Protein of unknown function
**121**	YIL016W		SNL1; YI3299.15; YIL016W Protein that interacts genetically with nuclear pore complex proteins
**122**	YIL026C	IRR1	IRR1; (SCC3); YI3299.05; YIL026C Component of cohesin complex required for sister chromatid cohesion during DNA replication
**123**	YIL131C	FKH1	FKH1; YIL131C Homolog of Drosophila forkhead protein, involved in transcriptional silencing, cell morphology and cell cycle
**124**	YIL132C		YIL132C Protein of unknown function
**125**	YIL140W	SRO4	AXL2; SRO4; BUD10; YIL140W Membrane glycoprotein localized at site of bud emergence, required for axial budding pattern
**126**	YIL141W		YIL141W Protein of unknown function questionable ORF
**127**	YJL002C	OST1	OST1; NLT1; (RIB1); J1404; YJL002C Oligosaccharyltransferase alpha subunit, member of a complex of 8 ER proteins that transfers coreoligosaccharide from dolichol carrier to Asn-X-Ser/Thr motif

**128**	YJL018W		YJL018W; J1315 Protein of unknown function, overlaps with YJL018W
**129**	YJL019W		YJL019W; J1310 Protein of unknown function, may be involved in chromatin structure, overlaps with YJL019W
**130**	YJL073W	JEM1	JEM1; KAR8; J1083; YJL073W DnaJ-like protein required for the nuclear fusion step of karyogamy
**131**	YJL074C	SMC3	SMC3; J1049; YJL074C Cohesin, coiled-coil protein of the SMC family involved in chromosome condensation and segregation
**132**	YJL091C		YJL091C; J0916 Protein of unknown function
**133**	YJL092W	HPR5	HPR5; RADH1; RADH SRS2; J0913; YJL092W DNA helicase involved in DNA repair suppressor of rad6 and rad18 mutations
**134**	YJL097W		YJL097W; J0902 Essential protein of unknown function
**135**	YJL115W	ASF1	ASF1; J0755; YJL115W Component of replication-coupling chromatin assembly factor (RCAF), target of the Rad53p-dependent DNA damageresponse
**136**	YJL158C	CIS3	CIS3; CCW5; CCW11; SCW8; J0561; YJL158C; PIR4 Cell wall protein with similarity to members of the Pir1p/Hsp150p/Pir3p family
**137**	YJL173C	RFA3	RFA3; J0506; YJL173C DNA replication factor A, 13K subunit
**138**	YJL181W		YJL181W; J0435 Protein of unknown function
**139**	YJL183W	MNN11	MNN11; J0425; YJL183W Subunit of the Anp1p-Hoc1p-Mnn10-Mnn11p-Mnn9p mannosyltransferase complex
**140**	YJL187C	SWE1	SWE1; J0406; YJL187C Serine/tyrosine dual-specificity protein kinase, phosphorylates Cdc28p on tyrosine and inhibits its activity
**141**	YJL196C	ELO1	ELO1; J0343; YJL196C Fatty acid elongation protein involved in elongation of tetradecanoic acid (14:0) to hexadecanoic acid (16:0)
**142**	YJL201W	ECM25	ECM25; J0325; YJL201W Protein possibly involved in cell wall structure or biosynthesis
**143**	YJR006W		HYS2; POL31; HUS2; SDP5; YJR83.7; J1427; YJR006W Small (58 kDa) subunit of DNA polymerase delta
**144**	YJR030C		YJR030C; J1575; J1575L Protein of unknown function
**145**	YJR031C	GEA1	GEA1; J1580; YJR031C Component of complex with guanine-nucleotide-exchange activity for ARF
**146**	YJR043C	POL32	POL32; J1626; GTF350; YJR043C Small (55 kDa) subunit of DNA polymerase delta
**147**	YJR075W	HOC1	HOC1; J1830; YJR075W Subunit of the Anp1p-Hoc1p-Mnn11p-Mnn9p mannosyltransferase complex of the Golgi involved in cell wall integrity
**148**	YJR076C	CDC11	CDC11; PSL9; J1833; YJR076C Septin, component of 10 nm filaments of mother-bud neck, involved in cytokinesis
**149**	YJR118C		ILM1; J2033; YJR118C Protein of unknown function, possibly involved in mitochondrial DNA maintenance
**150**	YJR143C	PMT4	PMT4; BAD15; J2176; YJR143C Mannosyltransferase (dolichyl phosphate-D-mannose:protein O-D-mannosyltransferase), involved in initiationof O-glycosylation
**151**	YJR144W	MGM101	MGM101; MGM9; J2181; YJR144W Mitochondrial genome maintenance protein
**152**	YKL008C		LAC1; (DGT1); YKL156; YKL008C Protein required with Lag1p for ER-to-Golgi transport of GPI-anchored proteins
**153**	YKL042W	SPC42	SPC42; YKL255; YKL042W Component of the spindle pole body
**154**	YKL045W	PRI2	PRI2; YKL258; YKL045W DNA primase large subunit (DNA polymerase alpha 58 kDa subunit), involved in synthesis of RNA primers for Okazakifragments

**155**	YKL049C	CSE4	CSE4; CSL2; YKL262; YKL049C Protein with similarity to histone H3, required for chromosome segregation
**156**	YKL089W	MIF2	MIF2; YKL089W Centromere protein required for normal chromosome segregation and spindle integrity
**157**	YKL101W	HSL1	HSL1; (SEL2); NIK1; YKL453; YKL101W Serine/threonine protein kinase that genetically interacts with histone mutants and negatively regulatesSwe1p protein kinase
**158**	YKL108W		SLD2; DRC1; YKL463; YKL108W Protein involved in DNA replication
**159**	YKL113C	RAD27	RAD27; RTH1; ERC11; (FEN1); YKL510; YKL113C Single-stranded DNA endonuclease and 5'-3' exonuclease that functions in theMSH2-MLH1-PMS1-dependent mismatch repair system
**160**	YKL165C	MCD4	MCD4; PER2; SSU21; ZRG16; YKL619; YKL165C Protein required for glycosylphosphatidylinositol (GPI) anchor synthesis
**161**	YKR010C	TOF2	TOF2; YK109; YKR010C Protein that interacts with DNA topoisomerase I
**162**	YKR012C		YKR012C Protein of unknown function questionable ORF
**163**	YKR013W	PRY2	PRY2; YFW12; YKR013W Protein expressed under starvation conditions
**164**	YKR037C		SPC34; YKR037C Protein of the spindle pole body
**165**	YKR077W		YKR077W Protein of unknown function
**166**	YKR083C		YKR083C; YKR403 Protein with similarity to paramyosin
**167**	YKR090W		YKR090W; YKR410 Protein of unknown function, contains two tandem LIM domains at the C-terminus
**168**	YLL002W	KIM2	REM50; KIM2; L1377; YLL002W Protein involved in resistance to mutagens such as diepoxybutane and mitomycin C
**169**	YLL021W	SPA2	SPA2; PEA1; PPF1; L1209; YLL021W Protein involved in cell polarity and cell fusion during mating
**170**	YLL022C		HIF1; L1205; YLL022C Protein that interacts with Hat1p histone acetyltransferase
**171**	YLR020C		YLR020C; L1709 Protein with similarity to triacylglycerol lipase
**172**	YLR032W	RAD5	RAD5; REV2; SNM2; L1767; YLR032W DNA helicase of the Snf2p family and member of the RAD6 epistasis group, involved in error-free DNArepair
**173**	YLR045C	STU2	STU2; L2108; YLR045C Component of the spindle pole body
**174**	YLR049C		YLR049C; L2121 Protein of unknown function
**175**	YLR050C		YLR050C; L2125 Protein with similarity to C-terminal region of human MAC30
**176**	YLR083C	EMP70	EMP70; L9449.11; L2385; YLR083C Endosomal membrane protein with similarity to human putative ion transporter EMP70
**177**	YLR103C	CDC45	CDC45; SLD4; L2720; L8004.11; YLR103C Protein required for initiation of chromosomal DNA replication
**178**	YLR154C		YLR154C; L3341 Protein of unknown function
**179**	YLR183C		TOS4; L9470.22; YLR183C Protein of unknown function
**180**	YLR212C	TUB4	TUB4; L8167.21; YLR212C Gamma tubulin, required for microtubule organization and nuclear division
**181**	YLR235C		YLR235C Protein of unknown function
**182**	YLR272C		YCS4; LOC7; L8479.14; YLR272C Subunit of condensin protein complex required for proper chromosome condensation and segregation
**183**	YLR300W	EXG1	EXG1; SCW6; BGL1; L8003.3; YLR300W Exo-beta-1,3-glucanase (I/II) major isoform involved in cell wall beta-glucan assembly

**184**	YLR313C	SPH1	SPH1; L8543.8; YLR313C Protein involved in polarized growth, with roles in shmoo formation and bud site selection
**185**	YLR314C	CDC3	CDC3; L8543.7; YLR314C Septin, component of 10 nm filaments of mother-bud neck involved in cytokinesis
**186**	YLR342W	FKS1	FKS1; GLS1; CWH53; ETG1; CND1; GSC1; PBR1; PBS1; L8300.6; YLR342W Component of beta-1,3-glucan synthase, probably functions asan alternate subunit with Gsc2p with which it has strong similarity
**187**	YLR372W	SUR4	SUR4; (APA1); VBM1; ELO3; SRE1; L8039.2; YLR372W Protein required for the conversion of 24-carbon fatty acids to 26-carbon fatty acids
**188**	YLR373C		VID22; L8039.16; YLR373C Plasma membrane associated protein required for targeting of fructose-1,6-bisphosphatase to Vid vesicles, has weaksimilarity to Von Willebrand factor
**189**	YLR381W		YLR381W; L3502.3 Protein of unknown function
**190**	YLR383W	RHC18	RHC18; L3502.2; YLR383W Protein involved in recombination repair, homologous to S. pombe rad18
**191**	YLR386W		YLR386W; L3502.1 Protein of unknown function
**192**	YLR459W	CDC91	CDC91; L9122.2; YLR459W Protein of unknown function
**193**	YML012W	ERV25	ERV25; YM9571.06; YML012W Component of COPII-coated vesicles
**194**	YML021C	UNG1	UNG1; YML021C Uracil-DNA glycolyase, removes uracil from DNA
**195**	YML027W	YOX1	YOX1; YML027W Protein with a homeodomain that binds tRNA-Leu gene
**196**	YML060W	OGG1	OGG1; YM9958.02; YML060W DNA glycosylase, excises 7,8-dihydro-8-oxoguanine (8-OxoG) and formamidopyrimidine (Fapy) residues fromDNA
**197**	YML061C	PIF1	PIF1; TST1; YM9958.01; YML061C Single-stranded DNA-dependent ATPase and 5'-3' DNA helicase required for maintenance and repair ofmitochondrial DNA, also functions in nucleus to regulate telomere length
**198**	YML085C	TUB1	TUB1; YML085C Tubulin alpha-1 chain, required for mitosis and karyogamy
**199**	YML102W	CAC2	CAC2; YML102W Chromatin assembly complex subunit 1, involved in nucleosome assembly linked with DNA replication, has WD (WD-40)repeats
**200**	YML109W	ZDS2	ZDS2; CES4; (MCS1); YM8339.10; YML109W Protein involved in regulation of transcriptional silencing and life span, multicopy suppressor ofsin4
**201**	YMR003W		YMR003W; YM8270.05 Protein of unknown function
**202**	YMR006C		PLB2; YM8270.08; YMR006C Phospholipase B2 (lysophospholipase), releases fatty acids from lysophospholipids
**203**	YMR048W		YMR048W Protein of unknown function
**204**	YMR076C	PDS5	PDS5; SPO27; YM9582.01; YM9916.15; YMR076C Protein required for sister chromatid cohesion and chromosome condensation functions
**205**	YMR078C	CTF18	CTF18; CHL12; YM9582.03; YMR078C Protein required for accurate chromosome transmission in mitosis and maintenance of normal telomerelength homolog of Rfc1p, Rfc2p, Rfc3p, Rfc4p, and Rfc5p
**206**	YMR144W		YMR144W; YM9375.13 Protein of unknown function, probable coiled-coil protein
**207**	YMR179W	SPT21	SPT21; BUR4; YM8010.09; YMR179W Protein that amplifies the magnitude of transcriptional regulation at various loci
**208**	YMR199W	CLN1	CLN1; PSC1; YM9646.13; YMR199W G1/S-specific cyclin that interacts with Cdc28p protein kinase to control events at START

**209**	YMR215W		YMR215W; YM8261.09; GAS3 Protein with similarity to Gas1p
**210**	YMR221C		YMR221C; YM9959.03 Protein of unknown function
**211**	YMR292W		GOT1; YMR292W Membrane protein required for ER to Golgi transport
**212**	YMR307W	GAS1	GAS1; GGP1; GP115; CWH52; YM9952.09; YMR307W 1,3-beta-glucanosyltransferase, glycophospholipid-anchored surface glycoprotein thatregulates the crosslinking of beta-1,6-glucans in the cell wall
**213**	YNL030W	HHF2	HHF2; CST22; H4II; N2752; YNL030W Histone H4, identical to Hhf1p
**214**	YNL031C	HHT2	HHT2; CST19; (SIN2); H3II; N2749; YNL031C Histone H3, core component of the nucleosome, identical to Hht1p
**215**	YNL039W	TFC5	TFC5; (TFC7); N2682; YNL039W Component of RNA polymerase III transcription factor TFIIIB, called B" or TFIIIB90
**216**	YNL072W	RNH35	RNH35; RNH2; N2369; YNL072W Ribonuclease H, endonuclease that degrades RNA in RNA-DNA hybrids
**217**	YNL082W	PMS1	PMS1; N2317; YNL082W Protein required for mismatch repair, homologous to E. coli MutL
**218**	YNL087W		YNL087W; N2250 Protein of unknown function
**219**	YNL102W	POL1	POL1; CDC17; CRT5; LRS9; HPR3; RRM1; N2181; YNL102W DNA polymerase I alpha 180 kDa subunit
**220**	YNL126W	SPC98	SPC98; N122; N1879; YNL126W Spindle pole body component that interacts with gamma-tubulin
**221**	YNL166C		BNI5; N1706; YNL166C Protein of unknown function, localizes to the mother-bud neck
**222**	YNL169C	PSD1	PSD1; N1692; YNL169C Phosphatidylserine decarboxylase, mitochondrial isozyme, converts phosphatidyl-L-serine to phosphatidylethanolamine
**223**	YNL181W		YNL181W; N1640 Protein of unknown function
**224**	YNL188W	KAR1	KAR1; N1611; YNL188W Component of spindle pole body half-bridge, required for spindle pole body duplication and karyogamy
**225**	YNL206C		RTT106; N1346; YNL206C Protein with similarity to SSRP proteins
**226**	YNL225C	CNM1	CNM67; CNM1; N1264; YNL225C Protein involved in nuclear migration and component of the spindle pole body
**227**	YNL233W	BNI4	BNI4; N1146; YNL233W Protein that may be involved in linking chitin synthase III to septins of the neck filaments
**228**	YNL262W	POL2	POL2; DUN2; N0825; YNL262W DNA polymerase epsilon large subunit, catalytic subunit essential for DNA replication and involved in DNArepair
**229**	YNL263C	YIF1	YIF1; SIF1; N0820; YNL263C Component of COPII vesicles, has similarity to NADH dehydrogenases
**230**	YNL273W	TOF1	TOF1; N0636; YNL273W Topoisomerase I interacting factor
**231**	YNL290W	RFC3	RFC3; N0533; YNL0533; YNL290W Replication factor C, 40 kDa subunit, member of the DEAD box family of putative helicase proteins
**232**	YNL300W		TOS6; YNL0430; N0430; YNL300W Probable cell wall protein, has weak similarity to Mid2p
**233**	YNL304W		YPT11; YNL0410; N0410; YNL304W Protein of unknown function, member of the rab family in the ras superfamily of small GTP-binding proteins
**234**	YNL309W	STB1	STB1; N0384; YNL309W Sin3p-binding protein, involved in transcription regulation at Start in the absence of Cln3p
**235**	YNL312W	RFA2	RFA2; BUF1; N0368; YNL312W DNA replication factor A, 36K subunit phosphorylated at the G1/S transition and dephosphorylated at mitosis
**236**	YNL313C		YNL313C; N0364 Protein of unknown function, has tetratricopeptide (TPR) repeats
**237**	YNR009W		YNR009W; N2044 Protein of unknown function

**238**	YOL007C		CSI2; O2373; YOL007C Protein involved in chitin synthesis
**239**	YOL012C	HTA3	HTZ1; HTA3; O2345; YOL012C Histone-related protein that can suppress histone H4 point mutation
**240**	YOL017W		YOL017W; O2321 Protein of unknown function
**241**	YOL019W		TOS7; O2313; YOL019W Protein of unknown function
**242**	YOL090W	MSH2	MSH2; O0935; YOL090W Component with Msh3p and Msh6p of DNA mismatch binding factor, involved in repair of single base mismatches andshort insertions/deletions
**243**	YOL149W	DCP1	DCP1; MRT2; AOC231; O0450; YOL149W mRNA decapping enzyme involved in mRNA turnover
**244**	YOR026W	BUB3	BUB3; PAC9; OR26.16; O2654; YOR026W Checkpoint protein required for cell cycle arrest in response to loss of microtubule function
**245**	YOR033C	DHS1	EXO1; DHS1; O2727; OR26.23; YOR033C Double-stranded DNA 5'->3' exonuclease, involved in mismatch repair and recombination
**246**	YOR073W		YOR073W; YOR29-24; O2945 Protein of unknown function
**247**	YOR074C	CDC21	CDC21; TMP1; O2950; YOR29-25; YOR074C Thymidylate synthase, catalyzes the reductive methylation of dUMP by5,10-methylene-5,6,7,8-tetrahydrofolate to produce dTMP and 7,8-dihydrofolate
**248**	YOR099W	KTR1	KTR1; YOR3189; O3189; YOR099W Mannosyltransferase of KRE2 family involved in N-linked and O-linked glycosylation
**249**	YOR114W		YOR114W; YOR3248; O3248 Protein of unknown function
**250**	YOR144C		EFD1; O3510; YOR3510; YOR144C Protein required for stability of direct DNA repeats
**251**	YOR175C		YOR175C; O3635 Protein with weak similarity to Drosophila nessy, a putative transmemberne protein expressed in embryogenesis
**252**	YOR247W		SRL1; O5273; YOR247W Protein with similarity to Svs1p
**253**	YOR248W		TOS11; O5276; YOR248W Protein of unknown function
**254**	YOR284W		YOR284W; O5483W Protein of unknown function
**255**	YOR320C		YOR320C; O6145 Protein with similarity to K. lactis N-acetylglucosaminyltransferase
**256**	YOR321W	PMT3	PMT3; O6148; YOR321W Mannosyltransferase (dolichyl phosphate-D-mannose:protein O-D-mannosyltransferase), involved in initiation ofO-glycosylation
**257**	YOR326W	MYO2	MYO2; CDC66; O6167; YOR326W Myosin heavy chain, class V, involved in polarized growth and secretion
**258**	YOR372C		NDD1; O6682; YOR372C Protein required for nuclear division positively but indirectly affects transcription of a subset of genes required for thecell cycle
**259**	YOR373W	NUD1	NUD1; O6685; YOR373W; SPC94 Spindle pole body protein required for nuclear division
**260**	YPL032C	SVL3	SVL3; P7102.17; YPL032C Protein involved in vacuolar uptake of endocytosed vital dyes
**261**	YPL050C	MNN9	MNN9; P7102.01; YPL050C Protein required for complex N-glycosylation, subunit of the Anp1p-Hoc1p-Mnn11p-Mnn9p and Van1p-Mnn9pmannosyltransferase complexes
**262**	YPL124W	NIP29	NIP29; LPH3; SPC29; YPL124W Protein of the spindle pole body
**263**	YPL127C	HHO1	HHO1; LPI17; YPL127C Histone H1
**264**	YPL128C	TBF1	TBF1; TBFALPHA LPI16; YPL128C Teleomere binding protein binds to TTAGGG repeats

**265**	YPL153C	RAD53	RAD53; MEC2; SPK1; (SAD1); P2588; YPL153C Serine/threonine/tyrosine protein kinase with a checkpoint function in S and G2
**266**	YPL163C	SVS1	SVS1; P2554; YPL163C Serine- and threonine-rich protein required for vanadate resistance
**267**	YPL208W		YPL208W; P1825 Protein of unknown function
**268**	YPL209C	IPL1	IPL1; PAC15; P1820; YPL209C Serine/threonine protein kinase of the mitotic spindle, involved in chromosome segregation
**269**	YPL227C	ALG5	ALG5; P1437; YPL227C Dolichol-P-glucose synthetase
**270**	YPL241C	CIN2	CIN2; P1043; YPL241C Protein involved in chromosome segregation
**271**	YPL255W	BBP1	BBP1; P0745; YPL255W Protein of the spindle pole body that binds to Bfr1p
**272**	YPL256C	CLN2	CLN2; P0741; PSC2; YPL256C G1/S-specific cyclin, interacts with Cdc28p protein kinase to control events at START
**273**	YPL267W		YPL267W; P0360 Protein of unknown function
**274**	YPR018W	RLF2	RLF2; CAC1; LRS8; PAX14; YP9531.12; YPR018W Chromatin assembly complex subunit 1; involved in nucleosome assembly linked with DNAreplication
**275**	YPR120C	CLB5	CLB5; P9642.8; YPR120C B-type cyclin appearing late in G1, involved in initiation of DNA synthesis
**276**	YPR135W	CTF4	CTF4; POB1; CHL15; P9659.7; YPR135W Protein required for DNA synthesis, binds DNA polymerase alpha
**277**	YPR174C		YPR174C; P9705.11 Protein with weak similarity to C. elegans nuclear lamin
**278**	YPR175W	DPB2	DPB2; P9705.7; YPR175W DNA polymerase epsilon 80 kDa subunit
**279**	YAL034WA		MTW1; YAL034W-A Essential kinetochore protein involved in mitosis
**280**	YER087CA		SBH1; SEB1; YER087C-A Component of the Sec61p-Sss1p-Sbh1p complex, involved in protein translocation into the endoplasmic reticulum

Cell-cycle M-G1 genes

Serial #	ORF	Name	description
**1**	YBR071W		YBR071W; YBR0712 Protein with weak similarity to Herpesvirus saimiri EERF2
**2**	YBR158W		CST13; ICS4; YBR1208; YBR158W Protein required for optimal growth and germination rate
**3**	YBR162C		TOS1; YBR1213; YBR162C Protein with similarity to Aga1p
**4**	YBR202W	CDC47	CDC47; MCM7; (MIS1); YBR1441; YBR202W Member of MCM/P1 family of proteins involved in DNA synthesis initiation
**5**	YDL047W	SIT4	SIT4; PPH1; D2693; YDL047W Protein serine/threonine phosphatase involved in cell cycle regulation, member of the PPP family of proteinphosphatases and related to PP2A phosphatase
**6**	YDL055C	PSA1	PSA1; MSN17; SRB1; VIG9; MPG1; D2566; YDL055C Mannose-1-phosphate guanyltransferase GDP-mannose pyrophosphorylase
**7**	YDL117W		CYK3; D2275; YDL117W Protein possibly involved in cytokinesis
**8**	YDL179W	PCL9	PCL9; D1408; YDL179W Cyclin that associates with Pho85p

**9**	YDR033W		MRH1; D3428; YDR033W; YD9673.03 Protein with similarity to Yro2p
**10**	YDR055W		PST1; YD9609.09; D4214; YDR055W Protein with similarity to members of the Sps2p-Ecm33p-Ycl048p family
**11**	YDR432W	NPL3	NPL3; NOP3; MTR13; MTS1; (NAB1); D9461.19; YDR432W Protein involved in 18S and 25S rRNA processing, export of RNA from the nucleus,import of proteins into the nucleus, associated with U1 snRNP, has 2 RNA recognition (RRM) domains
**12**	YDR433W		KRE22; YDR433W Protein of unknown function questionable ORF
**13**	YEL040W	UTR2	UTR2; CRH2; SYGP-ORF18; YEL040W Cell wall protein
**14**	YER036C		KRE30; YER036C Member of the non-transporter group of the ATP-binding cassette (ABC) superfamily
**15**	YER124C		YER124C; SYGP-ORF48 Protein of unknown function
**16**	YER154W	OXA1	OXA1; PET1402; PET-TS1402; HCY69; YER154W Protein required for assembly of F1F0-ATP synthase and cytochrome oxidase
**17**	YFL021W	GAT1	GAT1; NIL1; YFL021W GATA zinc finger transcription factor that plays a supplemental role to Gln3p, activating genes needed to usenon-preferred nitrogen sources
**18**	YGL028C		SCW11; G3661; YGL028C Putative cell wall protein with similarity to Scw10p
**19**	YGL055W	OLE1	OLE1; MDM2; G3472; YGL055W Stearoyl-CoA desaturase (delta-9 fatty acid desaturase), required for synthesis of unsaturated fatty acids
**20**	YGR041W	BUD9	BUD9; G4152; YGR041W Protein required for bipolar budding mutant diploid strains bud only at distal pole
**21**	YGR044C	RME1	RME1; CST5; RME (CSP1); G4306; YGR044C Zinc-finger transcription factor that represses meiosis in non-a/alpha cells
**22**	YGR086C		YGR086C; G4622 Protein of unknown function, expression is induced by high salt and low pH
**23**	YGR189C		CRH1; G7553; YGR189C Cell wall protein
**24**	YHR103W	SBE22	SBE22; YHR103W Protein required for bud growth
**25**	YHR128W	FUR1	FUR1; YHR128W Uracil phosphoribosyltransferase, part of the pyrimidine salvage pathway
**26**	YHR143W		YHR143W Protein of unknown function
**27**	YIL009W	FAA3	FAA3; YIL009W Acyl-CoA synthase (long-chain fatty acid CoA ligase) activates endogenous but not imported fatty acids
**28**	YJL078C	PRY3	PRY3; J1027; YJL078C Protein with similarity to plant pathenogenesis-related proteins, may have a role in mating efficiency
**29**	YJL157C	FAR1	FAR1; J0565; YJL157C Inhibitor of Cdc28p-Cln1p and Cdc28p-Cln2p kinase complexes involved in cell cycle arrest for mating
**30**	YJL159W		HSP150; CCW7; PIR2; ORE1; J0558; YJL159W Secreted O-glycosylated protein required for tolerance to heat shock, member ofPir1/Hsp150p/Pir3 family of proteins with variable number of tandem internal repeats
**31**	YJR147W		HMS2; J2204; YJR147W Transcription factor with a probable role in pseudohyphal growth
**32**	YKL116C		PRR1; YKL116C; YKL516 Serine/threonine protein kinase with similarity to S pombe nim1
**33**	YKL163W	PIR3	PIR3; CCW8; YKL617; YKL163W Protein with similarity to members of the Pir1p/Hsp150p/Pir3p family

**34**	YKL164C	PIR1	PIR1; CCW6; YKL618; YKL164C Protein required for tolerance to heat shock, member of the Pir1p/Hsp150p/Pir3p family
**35**	YKL182W	FAS1	FAS1; YKL182W Fatty-acyl-CoA synthase, beta chain, contains acetyl transferase, enoyl reductase, enoyl dehydratase, and malonyl/palmitoyltransferase activities in a single polypeptide
**36**	YKL185W	ASH1	ASH1; YKL185W GATA-type transcription factor that localizes preferentially to daughter cells to negatively regulate HO expression and isrequired for pseudohyphal growth
**37**	YKR077W		YKR077W Protein of unknown function
**38**	YLR049C		YLR049C; L2121 Protein of unknown function
**39**	YLR079W	SIC1	SIC1; SDB25; L9449.8; L2361; YLR079W Inhibitor of Cdc28p-Clb protein kinase complex
**40**	YLR153C	ACS2	ACS2; L9634.10; L3333; YLR153C Acetyl-CoA synthetase
**41**	YLR194C		YLR194C; L8167.13 Protein of unknown function
**42**	YLR286C	CTS1	CTS1; SCW2; CTS1-2; L8003.13; YLR286C Endochitinase
**43**	YLR300W	EXG1	EXG1; SCW6; BGL1; L8003.3; YLR300W Exo-beta-1,3-glucanase (I/II) major isoform involved in cell wall beta-glucan assembly
**44**	YLR399C	BDF1	BDF1; L8084.18; YLR399C Protein required for sporulation, has two bromodomain motifs and one ET domain
**45**	YLR413W		YLR413W; L9931.4 Protein of unknown function, induced during anaerobic growth
**46**	YNL046W		YNL046W; N2530 Protein of unknown function
**47**	YNL058C		YNL058C; YNL1621; YNL2433; N2433 Protein of unknown function
**48**	YNL066W	SUN4	SUN4; SCW3; YNL1612; YNL2411; N2411; YNL066W Protein involved in cell separation
**49**	YNL078W		YNL078W; N2337 Protein of unknown function
**50**	YNL192W	CHS1	CHS1; N1404; YNL192W Chitin synthase I, has a repair function during cell separation
**51**	YNL217W		YNL217W; N1306 Protein possibly involved in cytoplasmic ribosome function
**52**	YNL327W	EGT2	EGT2; N0320; YNL327W Cell-cycle regulation protein, may be involved in the correct timing of cell separation after cytokinesis
**53**	YNR017W	MAS6	TIM23; MAS6; MPI3; MIM23; N3180; YNR017W Mitochondrial inner membrane protein involved in import of proteins destined for themitochondrial matrix, part of the translocation channel with Tim17p/Mpi2p
**54**	YNR067C		YNR067C; N3547 Protein with similarity to C. albicans Eng1p, endo-1,3-beta-glucanase
**55**	YOL019W		TOS7; O2313; YOL019W Protein of unknown function
**56**	YOR066W		YOR066W; YOR29-17; O2920 Protein of unknown function
**57**	YOR263C		YOR263C; O5370 Protein with weak similarity to adenosine A1 receptors
**58**	YOR264W		YOR264W; O5375 Protein of unknown function
**59**	YOR315W		YOR315W; O6128 Protein of unknown function
**60**	YOR342C		YOR342C; O6283 Protein of unknown function
**61**	YPL158C		YPL158C; P2570 Protein of unknown function
**62**	YPL256C	CLN2	CLN2; P0741; PSC2; YPL256C G1/S-specific cyclin, interacts with Cdc28p protein kinase to control events at START

**63**	YPR106W	ISR1	ISR1; P8283.9; YPR106W Serine-threonine protein kinase involved in staurosporine resistance

Cell-cycle G2-M genes

Serial #	ORF	Name	description
**1**	YBL032W		YBL032W; YBL0418 Protein with four KH RNA-binding domains, has similarity to mouse hnRNP X protein
**2**	YBR038W	CHS2	CHS2; YBR0407; YBR038W Chitin synthase II, responsible for primary septum disk
**3**	YBR078W	ECM33	ECM33; YBR0727; YBR078W Protein with predicted GPI-anchor, involved in cell wall structure or biosynthesis
**4**	YBR086C		IST2; YBR0809; YBR086C Putative ion channel protein, has a role in sensitivity to NaCl
**5**	YBR092C	PHO3	PHO3; YBR0813; YBR092C Acid phosphatase, constitutive, thiamine-binding protein of the periplasmic space
**6**	YBR093C	PHO5	PHO5; YBR0814; YBR093C Acid phosphatase, repressible, requires glycosylation for activity
**7**	YBR187W		YBR187W; YBR1310 Protein of unknon function
**8**	YBR202W	CDC47	CDC47; MCM7; (MIS1); YBR1441; YBR202W Member of MCM/P1 family of proteins involved in DNA synthesis initiation
**9**	YCL014W	BUD3	BUD3; YCL013W; YCL014W; YCL012W Protein localized at the neck filament ring required for axial budding, may provide a memory of theprevious bud site
**10**	YCL063W		YCL063W; YCL062W Protein involved in sensitivity to certain drugs has similarity to plant aminocyclopropane-1-carboxylate synthase
**11**	YCR034W	FEN1	FEN1; GNS1; VBM2; ELO2; SYR3; YCR521; YCR34; YCR034W Protein involved in the elongation of fatty acids up to 24 carbons
**12**	YDR033W		MRH1; D3428; YDR033W; YD9673.03 Protein with similarity to Yro2p
**13**	YDR146C	SWI5	SWI5; YD8358.03; YDR146C Transcription factor that controls cell cycle-specific transcription of HO, has three tandem C2H2-type zinc fingers
**14**	YEL042W	GDA1	GDA1; SYGP-ORF16; YEL042W Guanosine diphosphatase of Golgi membrane
**15**	YER032W	FIR1	FIR1; PIP1; YER032W Protein probably involved in 3'-mRNA processing interacts with Pap1p and Ref2p,
**16**	YER110C	KAP123	KAP123; YRB4; YER110C Karyopherin-beta involved in nuclear import of ribosomal proteins
**17**	YFL037W	TUB2	TUB2; SHE8; ARM10; YFL037W Tubulin beta chain, required for mitosis and karyogamy
**18**	YGL008C	PMA1	PMA1; G3737; YGL008C H+-transporting P-type ATPase of the plasma membrane required for nutrient uptake and pH homeostasis, activity is ratelimiting for growth at low pH
**19**	YGL021W	ALK1	ALK1; G3686; YGL021W Protein of unknown function, predicted membrane protein, contains a putative leucine zipper domain
**20**	YGL101W		YGL101W; G3107 Protein of unknown function
**21**	YGL116W	CDC20	CDC20; PAC5; G2939; YGL116W Activator of anaphase promoting complex (APC), required for microtubule function at mitosis and for exit fromanaphase, contains WD (WD-40) repeats
**22**	YGR092W	DBF2	DBF2; G4643; YGR092W Serine/threonine protein kinase related to Dbf20p, required for events in anaphase/telophase

**23**	YGR108W	CLB1	CLB1; SCB1; G5967; YGR108W G2/M-phase-specific cyclin
**24**	YHL028W	WSC4	WSC4; YHC8; YFW1; YHL028W Protein required for secretory protein translocation, for maintenance of cell wall integrity, and for the stressresponse
**25**	YHR128W	FUR1	FUR1; YHR128W Uracil phosphoribosyltransferase, part of the pyrimidine salvage pathway
**26**	YIL106W	MOB1	MOB1; YIL106W Protein required for completion of mitosis and maintenance of ploidy
**27**	YIL131C	FKH1	FKH1; YIL131C Homolog of Drosophila forkhead protein, involved in transcriptional silencing, cell morphology and cell cycle
**28**	YIL158W		YIL158W Protein with similarity to Ykr100p
**29**	YJL051W		YJL051W; J1156 Protein of unknown function
**30**	YJL183W	MNN11	MNN11; J0425; YJL183W Subunit of the Anp1p-Hoc1p-Mnn10-Mnn11p-Mnn9p mannosyltransferase complex
**31**	YJR092W	BUD4	BUD4; J1905; YJR092W Protein required for axial budding but not for bipolar budding
**32**	YKL004W	AUR1	AUR1; ABR1; YKL004W; IPC1 Phosphatidylinositol:ceramide phosphoinositol transferase (IPC synthase), essential for sphingolipid synthesis
**33**	YKL130C		SHE2; YKL130C RNA-binding protein involved in localization of ASH1 mRNA, required for mother cell-specific expression of HO
**34**	YLR083C	EMP70	EMP70; L9449.11; L2385; YLR083C Endosomal membrane protein with similarity to human putative ion transporter EMP70
**35**	YLR084C		RAX2; L9449.12; YLR084C; L2389 Protein involved in bipolar budding
**36**	YLR131C	ACE2	ACE2; CIP3; L3123; L9606.10; YLR131C Metallothionein expression activator with similarity to Swi5p, has three tandem C2H2-type zinc fingers
**37**	YLR190W		YLR190W; L9470.2 Protein of unknown function
**38**	YML034W		SRC1; YML033W; YML034W Protein of unknown function, expression is cell cycle-regulated
**39**	YML052W	SUR7	SUR7; YM9958.11; YML052W Protein of unknown function overproduction suppresses the rvs167 mutation
**40**	YML058W		SML1; YM9958.04; YML058W Protein that negatively affects dNTP pools, binds ribonucleotide reductase and inhibits its activity
**41**	YML119W		YML119W; YM7056.07 Protein of unknown function
**42**	YMR001C	CDC5	CDC5; PKX2; MSD2; YM8270.03; YMR001C Serine/threonine protein kinase required for exit from mitosis may be involved in operation of themitotic spindle, member of the polo family of protein kinases
**43**	YMR032W		HOF1; CYK2; YM9973.05; YMR032W Protein involved in cytokinesis, has an SH3 domain
**44**	YMR215W		YMR215W; YM8261.09; GAS3 Protein with similarity to Gas1p
**45**	YNL057W		YNL057W; YNL1622; YNL2436; N2436 Protein of unknown function questionable ORF
**46**	YNL058C		YNL058C; YNL1621; YNL2433; N2433 Protein of unknown function
**47**	YNL066W	SUN4	SUN4; SCW3; YNL1612; YNL2411; N2411; YNL066W Protein involved in cell separation
**48**	YNL068C	FKH2	FKH2; N2403; YNL2403; YNL068C Homolog of Drosophila forkhead protein, involved in transcriptional silencing, cell morphology and cell cycle
**49**	YOL070C		YOL070C; O1150 Protein of unknown function

**50**	YOR025W	HST3	HST3; OR26.15; O2651; YOR025W Member of the Sir2p family of putative NAD-dependent histone acetylases involved in transcriptionalsilencing of chromatin
**51**	YOR315W		YOR315W; O6128 Protein of unknown function
**52**	YPL141C		YPL141C; LPI5 Serine/threonine protein kinase with similarity to Kin4p
**53**	YPL155C	KIP2	KIP2; P2581; YPL155C Kinesin-related protein
**54**	YPL242C	IQG1	IQG1; CYK1; P1041; YPL242C Protein involved in cytokinesis, has similarity to mammalian IQGAP proteins
**55**	YPR119W	CLB2	CLB2; P9642.6; YPR119W G2/M-phase-specific cyclin
**56**	YCR024CA		PMP1; YCR024C-A Plasma membrane proteolipid associated with Pma1p

**Phospholipids metabolism genes**			
**Serial #**	**ORF**	**Name**	**description**
**1**	YDR284C	DPP1	DPP1; (PAP1); D9819.10; YDR284C; ZRG1 Diacylglycerol pyrophosphate phosphatase
**2**	YDR497C	ITR1	ITR1; D9719.3; YDR497C Myo-inositol permease (major), closely related to Itr2p, member of the hexose transporter family of the major facilitatorsuperfamily (MFS)
**3**	YEL073C		YEL073C Protein of unknown function
**4**	YER026C	CHO1	CHO1; PSS PSS1; YER026C Phosphatidylserine synthase, CDP-diacylglycerol serine O-phosphatidyltransferase
**5**	YGL077C	HNM1	HNM1; (CTR1); CTR G3213; YGL077C Choline permease, member of the amino acid permease family of membrane transporters
**6**	YGL255W	ZRT1	ZRT1; NRC376; G0550; YGL255W High-affinity zinc transport protein, member of ZIP family of metal ion transporters
**7**	YGR026W		YGR026W; G4044 Protein with similarity to NADH-ubiquinone oxidoreductase chain 5
**8**	YGR149W		YGR149W; G6639 Protein with similarity to hypothetical protein A-288
**9**	YGR157W	CHO2	CHO2; PEM1; G6673; YGR157W Phosphatidylethanolamine N-methyltransferase, carries out first methylation step in the phosphatidylcholinebiosynthesis pathway
**10**	YHR123W	EPT1	EPT1; YHR123W sn-1,2-Diacylglycerol ethanolaminephosphotransferase, catalyzes the synthesis of phosphatidylethanolamine fromCDP-ethanolamine and diacylglycerol
**11**	YIL124W		AYR1; YI8277.05; YIL124W 1-Acyl dihydroxyacetone phosphate reductase
**12**	YJL153C	INO1	INO1; J0610; YJL153C Inositol-1-phosphate synthase (L-myo-inositol-1-phosphate synthase), functions in the inositol biosynthesis pathway
**13**	YJR073C	OPI3	OPI3; PEM2; YJR073C Phospholipid-N-methyltransferase, carries out the second and third methylation steps of the phosphatidylcholinebiosynthesis pathway
**14**	YKL035W		UGP1; YKL035W; YKL248 UDP-glucose pyrophosphorylase (UTP-glucose-1-P uridylyltransferase)
**15**	YKL100C		YKL100C; YKL450 Protein of unknown function
**16**	YKL175W		ZRT3; YKL640; YKL175W Vacuolar membrane protein involved in the regulation of zinc storage
**17**	YLR132C		YLR132C; L9606.11; L3127 Protein of unknown function

**18**	YNL130C	CPT1	CPT1; N1218; N1867; YNL130C sn-1,2-Diacylglycerol cholinephosphotransferase, catalyzes the synthesis of phosphatidylcholine fromCDP-choline and diacylglycerol
**19**	YNL169C	PSD1	PSD1; N1692; YNL169C Phosphatidylserine decarboxylase, mitochondrial isozyme, converts phosphatidyl-L-serine to phosphatidylethanolamine
**20**	YNL170W		YNL170W; N1688 Protein of unknown function, questionable ORF
**21**	YOL107W		YOL107W; HRA342; O0748 Protein with similarity to Rnh70p and Pan2p
**22**	YOR317W	FAA1	FAA1; O6136; YOR317W Long-chain fatty acid CoA ligase (fatty acid activator 1) can incorporate exogenous myristate into myristoyl-CoA andother fatty acids to the CoA derivatives

**Histones g**			
**Serial #**	**ORF**	**Name**	**description**
**1**	YBL002W	HTB2	HTB2; H2B2; YBL0104; YBL002W Histone H2B, nearly identical to Htb1p
**2**	YBL003C	HTA2	HTA2; H2A2; YBL0103; YBL003C Histone H2A, nearly identical to Hta1p
**3**	YBR009C	HHF1	HHF1; H4I; YBR0122; YBR009C Histone H4, identical to Hhf2p
**4**	YBR010W	HHT1	HHT1; H3I; (SIN2); BUR5; YBR0201; YBR010W Histone H3, identical to Hht2p
**5**	YBR078W	ECM33	ECM33; YBR0727; YBR078W Protein with predicted GPI-anchor, involved in cell wall structure or biosynthesis
**6**	YBR088C	POL30	POL30; PCNA YBR0811; YBR088C Proliferating cell nuclear antigen (PCNA), required for DNA synthesis and DNA repair
**7**	YBR089W		YBR089W; YBR0811a Protein of unknown function, questionable ORF
**8**	YCR034W	FEN1	FEN1; GNS1; VBM2; ELO2; SYR3; YCR521; YCR34; YCR034W Protein involved in the elongation of fatty acids up to 24 carbons
**9**	YDL003W	MCD1	MCD1; RHC21; (SCC1); PDS3; YD8119.04; D2940; YDL003W Cohesin, protein required for mitotic chromatid cohesion and chromosomecondensation
**10**	YDL055C	PSA1	PSA1; MSN17; SRB1; VIG9; MPG1; D2566; YDL055C Mannose-1-phosphate guanyltransferase GDP-mannose pyrophosphorylase
**11**	YDR224C	HTB1	HTB1; H2B1; SPT12; YD9934.09; YDR224C Histone H2B
**12**	YDR225W	HTA1	HTA1; SPT11; H2A1; YD9934.10; YDR225W Histone H2A, nearly identical to Hta2p
**13**	YDR297W	SUR2	SUR2; TSC7; SYR2; D9740.8; YDR297W Hydroxylase involved in sphingolipid metabolism
**14**	YDR451C		YHP1; D9461.36; YDR451C Protein with similarity to Yox1p, contains a homeodomain
**15**	YDR507C	GIN4	GIN4; ERC47; CLA6; D9719.13; YDR507C Serine/threonine-protein kinase required for septin organization at the bud neck, has similarity toYcl024p
**16**	YER003C	PMI40	PMI40; YER003C Mannose-6-phosphate isomerase, generates mannose-6-phosphate for synthesis of GDP-mannose anddolichol-phosphate-mannose
**17**	YER070W	RNR1	RNR1; SDS12; CRT7; YER070W Ribonucleotide reductase (ribonucleoside-diphosphate reductase) large subunit, converts ribonucleosidediphosphate to deoxyribonucleoside diphosphate

**18**	YER118C	SSU81	SHO1; SSU81; YER118C Osmosensor in the HOG1 MAP kinase, high-osmolarity signal transduction pathway, has an SH3 domain
**19**	YFL037W	TUB2	TUB2; SHE8; ARM10; YFL037W Tubulin beta chain, required for mitosis and karyogamy
**20**	YFL045C	SEC53	SEC53; ALG4; MCD5; YFL045C Phosphomannomutase, involved in the synthesis of GDP-mannose and dolichol-phosphate-mannose
**21**	YGL225W	GOG5	GOG5; VRG4; VAN2; MCD3; G1001; VIG4; YGL225W Golgi GDP-mannose transporter, member of the nucleotide-sugar transporter (NST) familyof membrane transporters
**22**	YGR151C		YGR151C; G6655 Protein of unknown function
**23**	YGR152C	RSR1	RSR1; BUD1; G6658; YGR152C GTP-binding protein involved in bud site selection, member of the ras family in the ras superfamily
**24**	YIL140W	SRO4	AXL2; SRO4; BUD10; YIL140W Membrane glycoprotein localized at site of bud emergence, required for axial budding pattern
**25**	YJL158C	CIS3	CIS3; CCW5; CCW11; SCW8; J0561; YJL158C; PIR4 Cell wall protein with similarity to members of the Pir1p/Hsp150p/Pir3p family
**26**	YJL187C	SWE1	SWE1; J0406; YJL187C Serine/tyrosine dual-specificity protein kinase, phosphorylates Cdc28p on tyrosine and inhibits its activity
**27**	YKL113C	RAD27	RAD27; RTH1; ERC11; (FEN1); YKL510; YKL113C Single-stranded DNA endonuclease and 5'-3' exonuclease that functions in theMSH2-MLH1-PMS1-dependent mismatch repair system
**28**	YLR183C		TOS4; L9470.22; YLR183C Protein of unknown function
**29**	YLR372W	SUR4	SUR4; (APA1); VBM1; ELO3; SRE1; L8039.2; YLR372W Protein required for the conversion of 24-carbon fatty acids to 26-carbon fatty acids
**30**	YML027W	YOX1	YOX1; YML027W Protein with a homeodomain that binds tRNA-Leu gene
**31**	YMR199W	CLN1	CLN1; PSC1; YM9646.13; YMR199W G1/S-specific cyclin that interacts with Cdc28p protein kinase to control events at START
**32**	YMR215W		YMR215W; YM8261.09; GAS3 Protein with similarity to Gas1p
**33**	YMR307W	GAS1	GAS1; GGP1; GP115; CWH52; YM9952.09; YMR307W 1,3-beta-glucanosyltransferase, glycophospholipid-anchored surface glycoprotein thatregulates the crosslinking of beta-1,6-glucans in the cell wall
**34**	YNL030W	HHF2	HHF2; CST22; H4II; N2752; YNL030W Histone H4, identical to Hhf1p
**35**	YNL031C	HHT2	HHT2; CST19; (SIN2); H3II; N2749; YNL031C Histone H3, core component of the nucleosome, identical to Hht1p
**36**	YNL300W		TOS6; YNL0430; N0430; YNL300W Probable cell wall protein, has weak similarity to Mid2p
**37**	YNR009W		YNR009W; N2044 Protein of unknown function
**38**	YOL007C		CSI2; O2373; YOL007C Protein involved in chitin synthesis
**39**	YOR247W		SRL1; O5273; YOR247W Protein with similarity to Svs1p
**40**	YOR248W		TOS11; O5276; YOR248W Protein of unknown function
**41**	YPL127C	HHO1	HHO1; LPI17; YPL127C Histone H1
**42**	YPL163C	SVS1	SVS1; P2554; YPL163C Serine- and threonine-rich protein required for vanadate resistance

**43**	YPL256C	CLN2	CLN2; P0741; PSC2; YPL256C G1/S-specific cyclin, interacts with Cdc28p protein kinase to control events at START

**Cell wall genes**			
**Serial #**	**ORF**	**Name**	**description**
**1**	YBR078W	ECM33	ECM33; YBR0727; YBR078W Protein with predicted GPI-anchor, involved in cell wall structure or biosynthesis
**2**	YCR034W	FEN1	FEN1; GNS1; VBM2; ELO2; SYR3; YCR521; YCR34; YCR034W Protein involved in the elongation of fatty acids up to 24 carbons
**3**	YDL055C	PSA1	PSA1; MSN17; SRB1; VIG9; MPG1; D2566; YDL055C Mannose-1-phosphate guanyltransferase GDP-mannose pyrophosphorylase
**4**	YEL042W	GDA1	GDA1; SYGP-ORF16; YEL042W Guanosine diphosphatase of Golgi membrane
**5**	YER003C	PMI40	PMI40; YER003C Mannose-6-phosphate isomerase, generates mannose-6-phosphate for synthesis of GDP-mannose anddolichol-phosphate-mannose
**6**	YER043C	SAH1	SAH1; YER043C Adenosylhomocysteinase
**7**	YFL037W	TUB2	TUB2; SHE8; ARM10; YFL037W Tubulin beta chain, required for mitosis and karyogamy
**8**	YFL045C	SEC53	SEC53; ALG4; MCD5; YFL045C Phosphomannomutase, involved in the synthesis of GDP-mannose and dolichol-phosphate-mannose
**9**	YGL225W	GOG5	GOG5; VRG4; VAN2; MCD3; G1001; VIG4; YGL225W Golgi GDP-mannose transporter, member of the nucleotide-sugar transporter (NST) familyof membrane transporters
**10**	YGR189C		CRH1; G7553; YGR189C Cell wall protein
**11**	YIL123W	SIM1	SIM1; (SAG1); YI8277.06; YIL123W Protein involved in the aging process and in regulation of the cell cycle
**12**	YJL158C	CIS3	CIS3; CCW5; CCW11; SCW8; J0561; YJL158C; PIR4 Cell wall protein with similarity to members of the Pir1p/Hsp150p/Pir3p family
**13**	YKL081W	TEF4	TEF4; (EFC1); YKL081W Translation elongation factor EF-1gamma
**14**	YKR013W	PRY2	PRY2; YFW12; YKR013W Protein expressed under starvation conditions
**15**	YLR300W	EXG1	EXG1; SCW6; BGL1; L8003.3; YLR300W Exo-beta-1,3-glucanase (I/II) major isoform involved in cell wall beta-glucan assembly
**16**	YLR372W	SUR4	SUR4; (APA1); VBM1; ELO3; SRE1; L8039.2; YLR372W Protein required for the conversion of 24-carbon fatty acids to 26-carbon fatty acids
**17**	YMR305C		SCW10; SCMP65; YM9952.07; YMR305C Protein with similarity to Scw4p, Bgl2p and other putative glucanases
**18**	YMR307W	GAS1	GAS1; GGP1; GP115; CWH52; YM9952.09; YMR307W 1,3-beta-glucanosyltransferase, glycophospholipid-anchored surface glycoprotein thatregulates the crosslinking of beta-1,6-glucans in the cell wall
**19**	YOL007C		CSI2; O2373; YOL007C Protein involved in chitin synthesis
**20**	YOR247W		SRL1; O5273; YOR247W Protein with similarity to Svs1p
**21**	YOR248W		TOS11; O5276; YOR248W Protein of unknown function

**22**	YPL163C	SVS1	SVS1; P2554; YPL163C Serine- and threonine-rich protein required for vanadate resistance
**23**	YPL256C	CLN2	CLN2; P0741; PSC2; YPL256C G1/S-specific cyclin, interacts with Cdc28p protein kinase to control events at START
**24**	YPR074C	TKL1	TKL1; YP9499.29; YPR074C Transketolase 1

**Mitochondrial ribosomal proteins genes**			
**Serial #**	**ORF**	**Name**	**description**
**1**	YBL038W	MRPL16	MRPL16; RML16; YBL0411; YBL038W Mitochondrial ribosomal protein of the large subunit (YmL47 E. coli L16), belongs to the L16 family ofprokaryotic ribosomal proteins
**2**	YBL090W	MRP21	MRP21; YBL0702; YBL090W Mitochondrial ribosomal protein of the small subunit
**3**	YBR120C	CBP6	CBP6; YBR0916; YBR120C Translational activator of COB mRNA
**4**	YBR122C	MRPL36	MRPL36; YML36; YBR0918; YBR122C Mitochondrial ribosomal protein of the large subunit
**5**	YBR146W	MRPS9	MRPS9; YBR1123; YBR146W Mitochondrial ribosomal protein of the small subunit S9, member of prokaryotic ribosomal protein S9 family
**6**	YBR185C	MBA1	MBA1; YBR1307; YBR185C Protein required for the assembly of mitochondrial respiratory complexes
**7**	YBR251W	MRPS5	MRPS5; YBR1704; YBR251W Mitochondrial ribosomal protein of the small subunit,homolog of E. coli ribosomal protein S5
**8**	YBR268W	MRPL37	MRPL37; YML37; YBR1736A; YBR268W Mitochondrial ribosomal protein of the large subunit
**9**	YBR282W	MRPL27	MRPL27; YML27; YBR2019; YBR282W Mitochondrial ribosomal protein of the large subunit
**10**	YCR003W	MRPL32	MRPL32; YML32; YCR041; YCR003W Mitochondrial ribosomal protein of the large subunit
**11**	YDR041W		RSM10; YD6888.03; D4150; YDR041W Component of the mitochondrial ribosomal small subunit
**12**	YDR079W	PET100	PET100; YD8554.12; D4441; YDR079W Protein required for assembly of cytochrome c oxidase
**13**	YDR115W		YDR115W; YD9727.10 Probable component of the mitochondrial ribosome, has similarity to prokaryotic L34 ribosomal protein
**14**	YDR116C		YDR116C; YD9727.11 Probable component of mitochondrial ribosomes, has similarity to prokaryotic ribosomal protein L1
**15**	YDR175C		RSM24; RSM52; YD9395.08; YDR175C Protein of the mitochondrial ribosomal small subunit
**16**	YDR237W	MRPL7	MRPL7; YD8419.04; YDR237W Mitochondrial ribosomal protein of the large subunit (YmL5/YmL7), belongs to the L5 family of prokaryoticribosomal proteins
**17**	YDR316W		YDR316W; D9740.1 Protein of unknown function, has predicted S-adenosylmethionine-dependent methyltransferase motif
**18**	YDR322W	MRPL35	MRPL35; YML35; D9798.5; YDR322W Mitochondrial ribosomal protein of the large subunit
**19**	YDR337W	MRPS28	MRPS28; D9651.3; YDR337W Mitochondrial ribosomal protein of the small subunit (E. coli S15)
**20**	YDR493W		YDR493W; D8035.36 Protein of unknown function
**21**	YDR494W		YDR494W; D8035.37 Protein of unknown function

**22**	YDR511W		ACN9; D9719.16; YDR511W Protein required for acetate utilization
**23**	YDR513W	TTR1	TTR1; GRX2; TTR D9719.17; YDR513W Glutaredoxin (thioltransferase, glutathione reductase)
**24**	YER050C		RSM18; YER050C Component of the mitochondrial ribosomal small subunit
**25**	YER058W	PET117	PET117; YER058W Protein involved in assembly of cytochrome oxidase
**26**	YER182W		YER182W Protein of unknown function
**27**	YFR011C		YFR011C Protein of unknown function
**28**	YGL068W		YGL068W; G3277 Putative mitochondrial ribosomal protein, possible homolog of E. coli L7/L12 ribosomal protein
**29**	YGL069C		YGL069C; G3274 Protein of unknown function questionable ORF
**30**	YGL129C		RSM23; RSM51; G2856; YGL129C Component of the mitochondrial ribosomal small subunit, required for respiration and mitochondrialmaintenance, putative ATPase
**31**	YGR021W		YGR021W; G4031 Protein of unknown function, likely involved in mitochondrial protein synthesis
**32**	YGR076C	MRPL25	MRPL25; YMR26; YML25; G4575; YGR076C Mitochondrial ribosomal protein of the large subunit YMR26 (YmL25)
**33**	YGR084C	MRP13	MRP13; G4618; YGR084C Mitochondrial ribosomal protein of the small subunit
**34**	YGR132C	PHB1	PHB1; PHB G6390; YGR132C Prohibitin, involved in determination of replicative lifespan member of the prohibitin complex with Phb2p
**35**	YGR165W		YGR165W; G7050 Protein of unknown function, may be involved in mitochondrial translation
**36**	YGR174C	CBP4	CBP4; G7122; YGR174C Ubiquinol-cytochrome c reductase assembly factor essential for assembly and stability of ubiquinol-cytochrome creductase
**37**	YGR215W		RSM27; RSM55; G7821; YGR215W Protein of the mitochondrial ribosome small subunit
**38**	YGR219W		YGR219W; ORF113; G8517 Protein of unknown function
**39**	YGR220C	MRPL9	MRPL9; YML9; ORF269; G8520; YGR220C Mitochondrial ribosomal protein of the large subunit (YmL9 E. coli L3; human MRL3)
**40**	YGR231C	PHB2	PHB2; ORF315; G8561; YGR231C Prohibitin, involved in determination of replicative lifespan member of the prohibitin complex with Phb1p
**41**	YHR116W		YHR116W Protein of unknown function, may be involved in mitochondrial translation
**42**	YHR147C	MRPL6	MRPL6; YHR147C Mitochondrial ribosomal protein of the large subunit (YmL16), belongs to L6 family of prokaryotic ribosomal proteins
**43**	YIL070C		MAM33; YIL070C Mitochondrial protein required for normal respiratory growth
**44**	YIL098C		FMC1; YIL098C Protein involved in production or assembly of mitochondrial cytochromes
**45**	YIL124W		AYR1; YI8277.05; YIL124W 1-Acyl dihydroxyacetone phosphate reductase
**46**	YIL157C		YIL157C Protein of unknown function
**47**	YJL096W		MRPL49; YML49; J0904; YJL096W Mitochondrial ribosomal protein of the large subunit (YmL49)
**48**	YJL104W		MIA1; J0822; YJL104W Mitochondrial protein with a role in respiration
**49**	YJL180C	ATP12	ATP12; J0486; YJL180C F1-ATP synthase assembly protein
**50**	YJR080C		YJR080C; J1847 Protein of unknown function

**51**	YJR101W		RSM26; RSM54; J1952; YJR101W Protein of the mitochondrial ribosome small subunit, required for respiratory growth, may have an indirect rolein stress resistance and pseudohyphal growth
**52**	YKL003C	MRP17	MRP17; YKL003C Mitochondrial ribosomal protein of the small subunit
**53**	YKL087C	CYT2	CYT2; YKL087C Holocytochrome c1 synthase (cytochrome c1 heme lyase)
**54**	YKL137W		YKL137W Protein required for respiratory growth, for normal distribution of actin patches, and for normal resistance to NaCl and H2O2
**55**	YKL138C	MRPL31	MRPL31; (YML31); YKL138C Mitochondrial ribosomal protein of the large subunit
**56**	YKL167C	MRP49	MRP49; YKL631; YKL167C Mitochondrial ribosomal protein of the large subunit,
**57**	YKL169C		YKL169C; YKL633 Protein of unknown function questionable ORF
**58**	YKL170W	MRPL38	MRPL38; YML38; YKL634; YKL170W Mitochondrial ribosomal protein of the large subunit (YmL38)
**59**	YKL195W		YKL195W Protein of unknown function
**60**	YKR006C	MRPL13	MRPL13; YK105; YKR006C Mitochondrial ribosomal protein of the large subunit
**61**	YKR016W		YKR016W Protein possibly involved in respiration
**62**	YKR065C		YKR065C Protein of unknown function
**63**	YKR085C	MRPL20	MRPL20; YML20; YKR405; YKR085C Mitochondrial ribosomal protein of the large subunit (YmL20)
**64**	YLL009C	COX17	COX17; L1343; YLL009C Cytoplasmic protein involved in delivery of copper ions to mitochondrial cytochrome oxidase
**65**	YLR069C	MEF1	MEF1; L2195; YLR069C Mitochondrial translation elongation factor G, promotes GTP-dependent translocation of nascent chain from A-site toP-site of ribosome
**66**	YLR204W	QRI5	QRI5; L8167.17A; YLR204W Protein of unknown function
**67**	YLR218C		YLR218C; L_F150 Protein of unknown function
**68**	YML009c	MRPL39	MRPL39; YML39; YM9571.09; YML009C Mitochondrial ribosomal protein of the large subunit
**69**	YML030W		YML030W Protein of unknown function, may be involved in mitochondrial translation
**70**	YML110C	DBI56	COQ5; TCM7; YM8339.09; YML110C; DBI56 Mitochondrial C-methyltransferase of the ubiquinone biosynthetic pathway
**71**	YML129C	COX14	COX14; M_F70 YM4987.06; YML129C Protein required for assembly of cytochrome oxidase
**72**	YMR157C		YMR157C; YM8520.06 Protein of unknown function, may be involved in mitochondrial translation
**73**	YMR158W		YMR158W; YM8520.07 Protein with weak similarity to E. coli ribosomal protein S8
**74**	YMR188C		YMR188C; YM8010.18 Probable mitochondrial ribosomal protein, has weak similarity to prokaryotic 30S ribosomal protein S17
**75**	YMR193W		MRPL24; MRP-L14; YML24; YML14; YM9646.05; YMR193W Mitochondrial ribosomal protein of the large subunit (YmL24)
**76**	YMR225C	MRPL44	MRPL44; YMR44; YM9959.07; YMR225C Mitochondrial ribosomal protein of the large subunit (YmR44)
**77**	YMR267W	PPA2	PPA2; IPP2; YM8156.09; YMR267W Inorganic pyrophosphatase, mitochondrial
**78**	YNL005C	MRP7	MRP7; MRPL2; N2007; YNL005C Mitochondrial ribosomal protein of the large subunit (YmL2)
**79**	YNL081C		YNL081C; N2322; YNI1 Putative mitochondrial ribosomal protein of the small subunit, has similarity to ribosomal protein S13 of prokaryotes andplant mitochondria

**80**	YNL100W		YNL100W; N2190 Protein of unknown function
**81**	YNL122C		YNL122C; N1901 Protein of unknown function
**82**	YNL137C	NAM9	NAM9; MNA6; N1211; N1840; YNL137C Mitochondrial ribosomal protein of the small subunit member of E. coli S4 superfamily
**83**	YNL177C		YNL177C; N1657 Protein of unknown function, may be involved in mitochondrial translation
**84**	YNL252C		MRPL17; YML30; YML17; N0864; YNL252C Mitochondrial ribosomal protein of the large subunit
**85**	YNL284C	MRPL10	MRPL10; N0580; YNL284C Mitochondrial ribosomal protein of the large subunit (YmL10), member of the L15 family of prokaryotic ribosomalproteins
**86**	YNL306W		YNL306W; YMS18; MRPS-18; YNL0397; N0397 Mitochondrial ribosomal protein of the small subunit (YmS18), has similarity to bacterial andchloroplast ribosomal protein S11
**87**	YNL315C	ATP11	ATP11; N0357; YNL315C F1-ATP synthase assembly protein
**88**	YNR022C		YNR022C; N3220 Protein of unknown function
**89**	YOL071W		YOL071W; O1145 Protein of unknown function
**90**	YOR020C	HSP10	HSP10; CPN10; OR26.10; O2634; YOR020C Mitochondrial chaperonin that cooperates with Hsp60p, counterpart of E. coli GroES
**91**	YOR150W		MRPL23; YML23; O3530; YOR150W Mitochondrial ribosomal protein of the large subunit
**92**	YOR158W	PET123	PET123; O3557; YOR158W Mitochondrial ribosomal protein of the small subunit
**93**	YOR187W		TUF1; TUFM O4741; YOR187W Translation elongation factor Tu, mitochondrial
**94**	YOR215C		YOR215C; YOR50-5; O5005 Protein of unknown function
**95**	YOR286W		YOR286W; O5489W Protein with similarity to Drosophila melanogaster heat shock protein 67B2
**96**	YPL087W		YDC1; LPG21; YPL087W Alkaline ceramidase with specificity for dihydroceramide
**97**	YPL118W		MRP51; LPH9; YPL118W Mitochondrial ribosomal protein of the small subunit
**98**	YPR099C		YPR099C; P8283.13A Protein of unknown function
**99**	YPR100W		YPR100W; P8283.12 Protein of unknown function
**100**	YPR166C	MRP2	MRP2; P9325.7; YPR166C Mitochondrial ribosomal protein of the small subunit

**Oxidative phosphorylation genes**			
**Serial #**	**ORF**	**Name**	**description**
**1**	YBL030C	PET9	PET9; AAC2; (ANC2); OP1; YBL0421; YBL030C ADP/ATP carrier protein of the mitochondrial carrier family (MCF) of membrane transporters
**2**	YBL045C	COR1	COR1; QCR1; YBL0403; YBL045C Ubiquinol cytochrome c reductase core protein 1, component of ubiquinol cytochrome c reductase complex
**3**	YBL099W	ATP1	ATP1; YBL0827; YBL099W Alpha subunit of F1-ATP synthase
**4**	YBR039W	ATP3	ATP3; YBR0408; YBR039W Gamma subunit of F1-ATP synthase
**5**	YBR183W		YPC1; YBR1305; YBR183W Alkaline ceramidase

**6**	YBR230C		YBR230C; YBR1527 Protein of unknown function
**7**	YDL004W	ATP16	ATP16; ATPDELTA YD8119.03; D2935; YDL004W Delta subunit of F1-ATP synthase
**8**	YDL067C	COX9	COX9; D2520; YDL067C Cytochrome c oxidase subunit VIIA, essential component of the cytochrome c holoenzyme
**9**	YDL181W	INH1	INH1; D1305; YDL181W Inhibitor of mitochondrial ATPase that forms a complex with ATP synthase to inhibit enzyme activity
**10**	YDR178W	SDH4	SDH4; YD9395.11; YDR178W Membrane anchor subunit in the succinate dehydrogenase complex
**11**	YDR298C	ATP5	ATP5; OSCP D9740.11; YDR298C Subunit 5 of F0-ATP synthase, oligomycin sensitivity-conferring subunit
**12**	YDR377W	ATP17	ATP17; D9481.21; YDR377W ATP synthase subunit f
**13**	YDR529C	QCR7	QCR7; UCR7; COR4; CRO1; D9719.32; YDR529C Ubiquinol cytochrome c reductase subunit 7 (14 kDa protein), component of ubiquinolcytochrome c reductase complex
**14**	YEL024W	RIP1	RIP1; YEL024W Ubiquinol cytochrome c reductase iron-sulfur protein (Rieske iron-sulfur protein), component of ubiquinol cytochrome creductase complex (cytochrome bc1 complex)
**15**	YER053C		YER053C Member of the mitochondrial carrier (MCF) family of membrane transporters, has similarity to C. elegans mitochondrial phosphate carrierprotein
**16**	YER141W	COX15	COX15; YER141W Protein required for cytochrome oxidase assembly
**17**	YFR033C	QCR6	QCR6; UCR6; COR3; CR17; YFR033C Ubiquinol cytochrome c reductase subunit 6, component of the ubiquinol cytochrome c reductase complex(cytochrome bc1 complex)
**18**	YGL187C	COX4	COX4; G1362; YGL187C Cytochrome c oxidase subunit IV
**19**	YGL191W	COX13	COX13; G1341; YGL191W Cytochrome-c oxidase chain VIa
**20**	YGR174C	CBP4	CBP4; G7122; YGR174C Ubiquinol-cytochrome c reductase assembly factor essential for assembly and stability of ubiquinol-cytochrome creductase
**21**	YGR182C		YGR182C; G7160 Protein of unknown function
**22**	YGR183C	QCR9	QCR9; UCR9; G7164; YGR183C Ubiquinol cytochrome c reductase subunit 9, component of ubiquinol cytochrome c reductase complex(cytochrome bc1 complex)
**23**	YHR051W	COX6	COX6; YHR051W Cytochrome c oxidase subunit VI
**24**	YJL166W	QCR8	QCR8; COR5; J0526; YJL166W Ubiquinol cytochrome c reductase subunit 8 (11 kDa protein) component of ubiquinol cytochrome c reductasecomplex (cytochrome bc1 complex)
**25**	YJR048W	CYC1	CYC1; GTA109; J1653; YJR048W Cytochrome-c isoform 1, predominant isoform during aerobic growth
**26**	YJR077C	MIR1	MIR1; J1837; YJR077C Phosphate transporter of the mitochondrial carrier (MCF) family
**27**	YJR121W	ATP2	ATP2; J2041; YJR121W Beta subunit of F1-ATP synthase, three copies are present in each F1 complex
**28**	YKL016C	ATP7	ATP7; YKL016C Subunit 7 of F0-ATP synthase, homolog of bovine F0-ATP synthase subunit d
**29**	YKL085W	MDH1	MDH1; ACN50; YKL085W Malate dehydrogenase, mitochondrial
**30**	YKL109W	HAP4	HAP4; YKL465; YKL109W Transcription factor with acidic activation domain, component of Hap2p-Hap3p-Hap4p-Hap5p complex involved inactivation of CCAAT box-containing genes
**31**	YKL141W	SDH3	SDH3; CYB3; YKL4; YKL141W Membrane anchor subunit for Sdh1p in the succinate dehydrogenase complex

**32**	YKL148C	SDH1	SDH1; SDHA HAR2; YKL602; YKL148C Succinate dehydrogenase (ubiquinone) flavoprotein (Fp) subunit, converts succinate plus ubiquinoneto fumarate plus ubiquinol in the TCA cycle
**33**	YKL150W	MCR1	MCR1; YKL605; YKL150W NADH-cytochrome b5 reductase
**34**	YLL041C	SDH2	SDH2; SDHB SDH L0745; YLL041C Succinate dehydrogenase (ubiquinone) iron-sulfur protein (Ip) subunit, converts succinate + ubiquinone tofumarate + ubiquinol in the TCA cycle
**35**	YLR038C	COX12	COX12; L1913; YLR038C Cytochrome-c oxidase, subunit VIb
**36**	YLR294C		YLR294C; L8003.19A Protein of unknown function, possibly involved in respiration
**37**	YLR295C	ATP14	ATP14; L8003.20; YLR295C Subunit h of ATP synthase
**38**	YLR395C	COX8	COX8; L8084.14; YLR395C Cytochrome c oxidase subunit VIII
**39**	YML120C	NDI1	NDI1; YM7056.06; YML120C NADH-ubiquinone oxidoreductase
**40**	YMR256c	COX7	COX7; YM9920.10; YMR256C Cytochrome c oxidase, subunit VII
**41**	YNL015W	PBI2	PBI2; N2844; YNL015W Protease B (yscB or Prb1p) inhibitor 2 (I2B), has activity related to vacuolar fusion that is not related to protease activity
**42**	YNL052W	COX5A	COX5A; YNL1628; YNL2474; N2474; YNL052W Cytochrome c oxidase subunit Va
**43**	YNL055C	POR1	POR1; OMP2; VDAC N2441; YNL1624; YNL2441; YNL055C Outer mitochondrial membrane porin (voltage-dependent anion-selective channel)
**44**	YNL100W		YNL100W; N2190 Protein of unknown function
**45**	YNR001C	CIT1	CIT1; GLU3; N2019; YNR001C Citrate synthase, mitochondrial, converts acetyl-CoA and oxaloacetate into citrate plus CoA
**46**	YOR065W	CYT1	CYT1; CTC1; HAR5; O2816; YOR29-16; YOR065W Cytochrome c1, member of the cytochrome bc1 complex has an intermembrane space (IMS)sorting signal that does not require Imp1p for cleavage
**47**	YPL078C	ATP4	ATP4; LPF7; YPL078C Subunit 4 of F0-ATP synthase, has similarity to bovine subunit b
**48**	YPL271W	ATP15	ATP15; P0345; YPL271W Epsilon subunit of F1-ATP synthase one copy is found in each F1 oligomer
**49**	YPR020W		ATP20; SU_G YP9531.14; YPR020W F1F0-ATP synthase complex F0 membrane domain g subunit
**50**	YPR191W	QCR2	QCR2; UCR2; COR2; COXCH2; P9677.6; YPR191W Ubiquinol cytochrome c reductase core protein 2, component of ubiquinol cytochrome creductase complex (cytochrome bc1 complex)
**51**	YHR001WA		QCR10; YHR001W-A Ubiquinol cytochrome c reductase subunit (8.5 kDa protein) component of the ubiquinol cytochrome c reductase complex

Amino-acid biosynthesis genes

Serial #	ORF	Name	description
**1**	YAL015C	NTG1	NTG1; FUN33; OGG2; YAL015C 8-oxoguanine:adenine glycosylase/lyase, involved in repair of oxidative DNA damage
**2**	YAL044C	GCV3	GCV3; FUN40; YAL044C Glycine decarboxylase hydrogen carrier protein H subunit (glycine cleavage H protein), functions in the pathway forglycine degradation

**3**	YAR009C		
**4**	YAR010C		
**5**	YBL033C	RIB1	RIB1; YBL0417; YBL033C GTP cyclohydrolase II, initial and rate-limiting step in the riboflavin biosynthesis pathway
**6**	YBL098W		YBL098W; YBL0828 Kynurenine 3-hydroxylase, NADPH-dependent flavin monooxygenase that catalyzes the hydroxylation of kynurenine to3-hydroxykynurenine in tryptophan degradation and nicotinic acid synthesis
**7**	YBL102W	SFT2	SFT2; YBL0812; YBL102W Membrane protein required for ER to Golgi transport, can suppress temperature-sensitive mutants of SED5 whenoverexpressed
**8**	YBL103C	RTG3	RTG3; YBL0810; YBL103C Basic helix-loop-helix (bHLH)-leucine zipper transcription factor involved in controlling metabolic interactions betweenmitochondria and peroxisomes
**9**	YBR012C		YBR012C; YBR0205 Protein of unknown function, has a peroxisomal targeting signal
**10**	YBR035C	PDX3	PDX3; YBR0321; YBR035C Pyridoxine (pyridoxamine) phosphate oxidase [P (N/M)P oxidase]
**11**	YBR043C		YBR043C; YBR0413 Member of the multidrug-resistance 12-spanner (DHA12) family of the major facilitator superfamily (MFS-MDR)
**12**	YBR046C	ZTA1	ZTA1; YBR046C; YBR0421 Zeta-crystallin homolog, has similarity to E. coli quinone oxidoreductase and human zeta-crystallin which has quinoneoxidoreductase activity
**13**	YBR047W		YBR047W; YBR0422 Protein of unknown function transcription induced during aerobic growth, also induced by the drug FK506 in aGCN4-dependent manner
**14**	YBR104W	YMC2	YMC2; YBR0833; YBR104W Member of the mitochondrial carrier (MCF) family of membrane transporters
**15**	YBR105C		VID24; YBR0834; YBR105C Protein required for vacuolar import and degradation of Fbp1p (fructose-1,6-bisphosphatase)
**16**	YBR115C	LYS2	LYS2; ly2; YBR0910; YBR115C Alpha-aminoadipate-semialdehyde dehydrogenase large chain, sixth step in lysine biosynthesis pathway
**17**	YBR145W	ADH5	ADH5; YBR1122; YBR145W Alcohol dehydrogenase, zinc-dependent, also called formaldehyde dehydrogenase
**18**	YBR147W		YBR147W; YBR1124 Protein of unknown function, has 7 potential transmembrane segments transcription induced by the drug FK506 in aGCN4-dependent manner
**19**	YBR166C	TYR1	TYR1; YBR1218; YBR166C Prephenate dehydrogenase (NADP+), step of tyrosine biosynthesis pathway
**20**	YBR218C	PYC2	PYC2; YBR1507; YBR218C Pyruvate carboxylase 2
**21**	YBR228W		SLX1; YBR1525; YBR228W Subunit of Slx1p-Slx4p complex, required for cell growth in the absence of SGS1 or TOP3
**22**	YBR248C	HIS7	HIS7; YBR1640; YBR248C Glutamine amidotransferase:cyclase, catalyzes the fifth and sixth steps in the histidine biosynthesis pathway
**23**	YBR249C	ARO4	ARO4; YBR1701; YBR249C 2-Dehydro-3-deoxyphosphoheptonate aldolase (3-deoxy-D-arabino-heptulosonate-7-phosphate synthase DAHPsynthase), tyrosine-inhibited
**24**	YBR256C	RIB5	RIB5; YBR1724; YBR256C Riboflavin synthase, last step of riboflavin synthesis, converts 6,7-dimethyl-8-ribityllumazine to riboflavin

**25**	YBR294W	SUL1	SUL1; SFP YBR2110; YBR294W Sulfate permease (high-affinity sulfate transporter), member of the sulfate permease family of membranetransporters
**26**	YBR296C		PHO89; ITN1; YBR2113; YBR296C High-affinity Na+-dependent phosphate transporter
**27**	YBR297W	MAL33	MAL33; MAL3R; YBR2115; YBR297W Maltose fermentation regulatory protein, has a Zn[2]-Cys[6] fungal-type binuclear cluster domain
**28**	YCL009C	ILV6	ILV6; YCL009C Acetolactate synthase regulatory subunit
**29**	YCL019W		
**30**	YCL030C	HIS4	HIS4; YCL183; YCL030C Phosphoribosyl-AMP cyclohydrolase / phosphoribosyl-ATP pyrophosphohydrolase / histidinol dehydrogenase,second, third, and tenth steps of histidine biosynthesis pathway
**31**	YCR023C		YCR023C; YCR241 Member of the multidrug-resistance 12-spanner (DHA12) family of the major facilitator superfamily (MFS-MDR)
**32**	YCR053W	THR4	THR4; LST5; YCR053W Threonine synthase (O-P-homoserine P-lyase), second and final step of threonine biosynthesis pathway
**33**	YCR099C		YCR099C Protein with strong similarity to Pep1p; probably a fragmented coding region of a pseudogene
**34**	YCR100C		YCR100C Protein with similarity to Pep1p; probably represents a fragmented coding region of a pseudogene
**35**	YCR106W		YCR106W Protein with similarity to transcription factors, has Zn[2]-Cys[6] fungal-type binuclear cluster domain in the N-terminal region
**36**	YDL025C		YDL025C; D2810 Serine/threonine protein kinase with similarity to members of the NPR1 subfamily
**37**	YDL054C		YDL054C; D2570 Protein of unknown function
**38**	YDL066W	IDP1	IDP1; D2522; YDL066W Isocitrate dehydrogenase (NADP+), mitochondrial
**39**	YDL131W	LYS21	LYS21; D2195; YDL131W Homocitrate synthase isoenzyme, involved in lysine metabolism
**40**	YDL170W	UGA3	UGA3; (DUR3); D1463; YDL170W Transcriptional activator for 4-aminobutyric acid (GABA) catabolic genes, including UGA4, UGA1, and UGS2
**41**	YDL171C	GLT1	GLT1; D1448; YDL171C Glutamate synthase (NADPH, GOGAT) involved with glutamine synthetase (Gln1p) in glutamate biosynthesis
**42**	YDL182W	LYS20	LYS20; D1298; YDL182W Homocitrate synthase isoenzyme, involved in lysine biosynthesis
**43**	YDL183C		YDL183C; D1293 Protein of unknown function, has potential mitochondrial import sequence
**44**	YDL198C	YHM1	YHM1; (SHM1); D1214; YDL198C Protein of the mitochondrial carrier (MCF) family that when overexpressed suppresses loss of Abf2p
**45**	YDL199C		YDL199C; D1209 Member of the hexose transporter family of the major facilitator superfamily (MFS)
**46**	YDR011W	SNQ2	SNQ2; PZB1501; YD8119.16; D3213; YDR011W Drug-efflux pump involved in resistance to multiple drugs, member of the ATP-binding cassette(ABC) superfamily
**47**	YDR019C	GCV1	GCV1; GSD1; PZD400; D3250; YDR019C Glycine decarboxylase T subunit (glycine cleavage T protein), functions in the pathway for glycinedegradation
**48**	YDR035W	ARO3	ARO3; YD9673.07; D3453; YDR035W 2-Dehydro-3-deoxyphosphoheptonate aldolase (DAHPS) phenylalanine-inhibited

**49**	YDR037W	KRS1	KRS1; GCD5; YD9673.09; D3457; YDR037W Lysyl-tRNA synthetase, cytoplasmic
**50**	YDR126W		PSL10; YD9302.01; YD9727.21; YDR126W Protein of unknown function
**51**	YDR127W	ARO1	ARO1; AROM YD9302.02; YDR127W Arom pentafunctional enzyme
**52**	YDR158W	HOM2	HOM2; THR2; YD8358.12; YDR158W Aspartate-semialdehyde dehydrogenase (L-aspartate-beta-semialdehyde:NADP oxidoreductase[phosphorylating]), second step in common pathway for methionine and threonine biosynthesis
**53**	YDR186C		YDR186C; YD9395.20 Protein of unknown function
**54**	YDR234W	LYS4	LYS4; LYS3; ly3; ly4; YD8419.01; YD9934.18; YDR234W Homoaconitate hydratase (homoaconitase), third step in lysine biosynthesis pathway,converts homoaconitate to homoisocitrate
**55**	YDR242w	AMD2	AMD2; AMDY1; AMDY (AMD1); YD8419.09; YDR242W Protein with similarity to amidases
**56**	YDR341C		YDR341C; (RRS1); D9651.10 Arginine-tRNA synthetase, cytoplasmic, member of class I family of aminoacyl-tRNA synthetases
**57**	YDR354W	TRP4	TRP4; D9476.4; YDR354W Anthranilate phosphoribosyltransferase, second step in tryptophan biosynthesis pathway
**58**	YDR408C	ADE8	ADE8; D9509.26; YDR408C Phosphoribosylglycinamide formyltransferase (GART), third step in de novo purine biosynthesis pathway
**59**	YDR425W		YDR425W; D9461.13 Protein of unknown function
**60**	YDR426C		YDR426C; D9461.13A Protein of unknown function
**61**	YDR481C	PHO8	PHO8; D8035.24; YDR481C Vacuolar alkaline phosphatase (ALP), repressible, carries out dephosphorylation of phosphopeptides
**62**	YDR487C	RIB3	RIB3; D8035.30; YDR487C DBP synthase (3,4-dihydroxy-2-butanone 4-phosphate synthase), part of the riboflavin biosynthesis pathway
**63**	YDR530C	APA2	APA2; D9719.33; YDR530C ATP adenylyltransferase II
**64**	YDR531W		YDR531W; D9719.34 Putative pantothenate kinase involved in coenzyme A biosynthesis
**65**	YEL009C	GCN4	GCN4; ARG9; AAS3; YEL009C Transcription factor of the basic leucine zipper (bZIP) family, regulates general control in response to amino acidor purine starvation
**66**	YEL029C		BUD16; YEL029C Protein with similarity to putative Salmonella phosphotransferase system transcriptional regulator ptsJ
**67**	YEL045C		YEL045C; SYGP-ORF33 Protein of unknown function, has motifs typical of ATP/GTP binding sites
**68**	YEL046C	GLY1	GLY1; SYGP-ORF34; YEL046C Threonine aldolase, required for glycine biosynthesis
**69**	YEL063C	CAN1	CAN1; arg-p1; YEL063C Permease for basic amino acids, arginine, lysine, and histidine
**70**	YEL071W		DLD3; YEL071W D-lactate dehydrogenase
**71**	YER024W		YER024W Protein with similarity to Yat1p
**72**	YER040W	GLN3	GLN3; YER040W GATA-type zinc finger transcription factor for positive nitrogen regulation
**73**	YER052C	HOM3	HOM3; THR3; BOR1; YER052C Aspartate kinase (L-aspartate 4-P-transferase), catalyzes the first step in the common pathway for methionine andthreonine biosynthesis
**74**	YER055C	HIS1	HIS1; YER055C ATP phosphoribosyltransferase, first step in histidine biosynthesis pathway

**75**	YER056C	FCY2	FCY2; BRA7; YER056C Cytosine/purine permease, member of the purine/cytosine permease family of membrane transporters
**76**	YER061C	CEM1	CEM1; YER061C Beta-ketoacyl-ACP synthase, mitochondrial (3-oxoacyl-[Acyl-carrier-protein] synthase)
**77**	YER069W	ARG5,6	ARG5,6; ARG5; ARG6; argC argB YER069W Acetylglutamate kinase and N-acetyl-gamma-glutamyl-phosphate reductase, which catalyze thesecond and third steps of ornithine and arginine biosynthesis
**78**	YER073W		ALD5; YER073W Mitochondrial aldehyde dehydrogenase
**79**	YER081W		SER3; YER081W 3-phosphoglycerate dehydrogenase involved in synthesis of serine from 3-phosphoglycerate
**80**	YER086W	ILV1	ILV1; (ISO1); YER086W Serine and threonine dehydratase (anabolic), first step in isoleucine biosynthesis pathway
**81**	YER090W	TRP2	TRP2; YER090W Component I of anthranilate synthase:indole-3-glycerol phosphate synthase (anthranilate synthase) involved in the first step ofthe tryptophan biosynthesis pathway
**82**	YER091C	MET6	MET6; YER091C Homocysteine methyltransferase (5-methyltetrahydropteroyl triglutamate-homocysteine methyltransferase), methionine synthase,cobalamin-independent
**83**	YER128W		YER128W; SYGP-ORF44 Protein of unknown function
**84**	YER138C		
**85**	YER160C		
**86**	YER175C		YER175C; SYGP-ORF63 Protein with predicted S-adenosylmethionine-dependent methyltransferase motif, has similarity to Yhr209p; transcriptioninduced by the drug FK506 in a GCN4-dependent manner
**87**	YFL010C		YFL010C Protein of unknown function
**88**	YFL028C	CAF16	CAF16; YFL028C Protein that associates with Ccr4p, member of the non-transporter group of the ATP-binding cassette (ABC) superfamily
**89**	YFR025C	HIS2	HIS2; YFR025C Histidinol phosphatase
**90**	YFR030W	MET10	MET10; YFR030W Assimilatory sulfite reductase subunit, flavin-binding (alpha) subunit, part of the sulfate assimilation pathway
**91**	YFR055W		YFR055W Protein with similarity to E. coli cystathionine beta-lyase
**92**	YGL009C	LEU1	LEU1; G3734; YGL009C 3-Isopropylmalate dehydratase, second step in leucine biosynthesis pathway
**93**	YGL012W	ERG4	ERG4; NYS4; YGL022; G3725; YGL012W Sterol C-24 (28) reductase
**94**	YGL026C	TRP5	TRP5; G3669; YGL026C Tryptophan synthase, last (fifth) step in tryptophan biosynthesis pathway
**95**	YGL062W	PYC1	PYC1; PYV G3428; YGL062W Pyruvate carboxylase 1, catalyzes conversion of pyruvate to oxaloacetate in the gluconeogenesis pathway
**96**	YGL114W		YGL114W; G2950 Protein member of the oligopeptide transporter (OPT) family of membrane transporters
**97**	YGL117W		YGL117W; G2935 Protein of unknown function transcription induced by the drug FK506 in a GCN4-dependent manner
**98**	YGL125W	MET11	MET13; MET11; MRPL45; G2882; YGL125W Methylenetetrahydrofolate reductase (MTHFR), essential for methionine biosynthesis
**99**	YGL148W	ARO2	ARO2; G2501; YGL148W Chorismate synthase
**100**	YGL154C	LYS5	LYS5; ly5; G1867; YGL154C Alpha-aminoadipate reductase small chain, sixth step in lysine biosynthesis pathway

**101**	YGL180W	APG1	APG1; AUT3; G1615; YGL180W Serine/threonine protein kinase involved in induction of autophagy after nutrient limitation
**102**	YGL184C		STR3; G1601; YGL184C Protein with strong similarity to cystathionine beta-lyase transcription induced by the drug FK506 in a GCN4-dependentmanner
**103**	YGL186C		YGL186C; G1370 Member of the purine/cytosine permease family of membrane transporters
**104**	YGL202W	ARO8	ARO8; G1253; YGL202W Aromatic amino acid aminotransferase I
**105**	YGL215W	CLG1	CLG1; G1105; YGL215W Cyclin-like protein, associates with Pho85p
**106**	YGL223C		YGL223C; G1010 Protein of unknown function
**107**	YGL224C		YGL224C; G1005 Protein of unknown function
**108**	YGL234W	ADE5,7	ADE5,7; ADE5; ADE7; G0930; YGL234W Phosphoribosylamine-glycine ligase (GARSase) plus Phosphoribosylformylglycinamidine cyclo-ligase(AIRSase) bifunctional protein
**109**	YGL260W		YGL260W; NRB76; G0332 Protein with strong similarity to Yir040p and members of the Ybl108p/Ycr103p/Ykl223p family
**110**	YGR029W	ERV1	ERV1; G4062; YGR029W Flavin-linked sulfhydryl oxidase essential for mitochondrial biogenesis and cell viability
**111**	YGR061C	ADE6	ADE6; G4530; YGR061C 5'-phosphoribosylformyl glycinamidine synthetase, has glutamine amidotransferase domain and aminator domain
**112**	YGR065C		VHT1; G4539; YGR065C H+/biotin symporter and member of the allantoate permease family of the major facilitator superfamily (MFS)
**113**	YGR110W		YGR110W; G6140 Protein of unknown function, induced during aerobic growth
**114**	YGR124W	ASN2	ASN2; G6358; YGR124W Asparagine synthetase (L-aspartate: L-glutamine amidoligase [AMP-forming]), Asn1p and Asn2p are isozymes
**115**	YGR153W		TOS10; G6661; YGR153W Protein of unknown function
**116**	YGR154C		YGR154C; G6664 Putative paralog of Ecm4p
**117**	YGR185C	TYS1	TYS1; TYRRS G7522; YGR185C Tyrosyl-tRNA synthetase, cytoplasmic, charges its cognate tRNAs with tyrosine during protein synthesis
**118**	YGR204W	ADE3	ADE3; G7733; YGR204W C1-tetrahydrofolate synthase (trifunctional enzyme), cytoplasmic
**119**	YGR239C		PEX21; ORF288; G8593; YGR239C Peroxisomal biogenesis protein (peroxin), involved with Pex18p in Pex7p-mediated peroxisomal proteintargeting
**120**	YGR267C	FOL2	FOL2; G9349; YGR267C GTP cyclohydrolase I, catalyzes synthesis of D-erythro-7,8-dihydroneopterintriphosphate from GTP
**121**	YGR288W		MAL13; MALR G9574; YGR288W Maltose pathway regulatory protein, contains a Zn[2]-Cys[6] fungal-type binuclear cluster domain
**122**	YHL027W	RIM101	RIM101; (RIM1); YHL027W Transcription factor involved in induction of IME1, IME2, DIT1, and DIT2 transcription, has three C2H2-type zincfingers
**123**	YHR006W	STP2	STP2; YHR006W Protein with strong similarity to Stp1p, which is involved in tRNA splicing and branched-chain amino acid uptake

**124**	YHR018C	ARG4	ARG4; argH YHR018C Argininosuccinate lyase, catalyzes the final step in arginine biosynthesis
**125**	YHR019C	DED81	DED81; asnRS YHR019C Asparaginyl-tRNA synthetase, cytoplasmic
**126**	YHR025W	THR1	THR1; YHR025W Homoserine kinase (ATP:L-homoserine-O-P-transferase), first step in the threonine biosynthesis pathway
**127**	YHR029C		YHR029C Protein with similarity to Pseudomonas aureofaciens phzC involved in phenazine antibiotic synthesis transcription induced by the drugFK506 in a GCN4-dependent manner
**128**	YHR047C	AAP1'	AAP1; H8179.24; YHR047C Alanine/arginine aminopeptidase, highly related to aminopeptidase Ape2p (yscII) and other zinc metalloproteases
**129**	YHR071W	PCL5	PCL5; H8025.1; YHR071W Cyclin that associates with Pho85p
**130**	YHR122W		YHR122W Protein of unknown function
**131**	YHR162W		YHR162W Protein of unknown function
**132**	YHR208W	BAT1	BAT1; BAA1; TWT1; ECA39; YHR208W Mitochondrial branched-chain amino acid transaminase
**133**	YIL003W		YIL003W; YIA3 Essential protein of unknown function, member of the nucleotide binding protein family
**134**	YIL056W		YIL056W Protein of unknown function
**135**	YIL060W		YIL060W Protein of unknown function
**136**	YIL074C		SER33; YIL074C 3-phosphoglycerate dehydrogenase involved in synthesis of serine from 3-phosphoglycerate
**137**	YIL094C		LYS12; LYS11; LYS10; YI9910.02; YIL094C Homoisocitrate dehydrogenase, converts homoisocitrate to alpha-ketoadipate, the fourth step in thelysine biosynthesis pathway
**138**	YIL116W	HIS5	HIS5; YIL116W Histidinol-phosphate aminotransferase (imidazole acetol phosphate aminotransferase), eighth step in histidine biosynthesispathway
**139**	YIL117C	PRM5	PRM5; YIL117C Protein of unknown function regulated by pheromone
**140**	YIL163C		YIL163C; YI9402.11 Protein of unknown function
**141**	YIL164C	NIT1	NIT1; YI9402.10B; YIL164C Protein with similarity to nitrilases, may be a pseudogene or separated from YIL165C by sequencing errors
**142**	YIL165C		NIT1; YI9402.10B; YIL164C Protein with similarity to nitrilases, may be a pseudogene or separated from YIL165C by sequencing errors
**143**	YIL173W		VTH1; YI9402.04; YIL173W Protein with strong similarity to Vth2p and Pep1p, potential membrane glycoprotein
**144**	YIR005W		IST3; YIB5; YIR005W Protein with a role in sodium tolerance, has one RNA recognition (RRM) domain
**145**	YIR017C	MET28	MET28; YIR017C Transcriptional activator regulating sulfur amino acid metabolism that functions with Met4p and Cbf1p, member of the basicleucine zipper (bZIP) family
**146**	YIR034C	LYS1	LYS1; ly1; YIR034C Saccharopine dehydrogenase (NAD+, L-lysine forming), catalyzes the eighth and final step in lysine biosynthesis pathway
**147**	YJL060W		YJL060W; J1138 Protein of unknown function has similarity to kynurenine aminotransferase
**148**	YJL071W	ARG2	ARG2; argA HRB574; J1091; YJL071W Acetylglutamate synthase, catalyzes the first step in ornithine and arginine biosynthesis
**149**	YJL072C		YJL072C; HRF213; J1086 Protein of unknown function

**150**	YJL088W	ARG3	ARG3; argF J0924; YJL088W Ornithine carbamyltransferase, catalyzes the sixth step in the arginine biosynthesis pathway
**151**	YJL089W	SIP4	SIP4; J0922; YJL089W Transcriptional activator of gluconeogenic genes through CSRE elements, activated by Snf1p kinase, contains aZn[2]-Cys[6] fungal-type binuclear cluster domain
**152**	YJL160C		YJL160C; J0555 Protein with similarity to members of the Pir1p/Hsp150p/Pir3p family
**153**	YJL172W	CPS1	CPS1; CPS J0510; YJL172W Gly-X carboxypeptidase yscS, involved in nitrogen metabolism
**154**	YJL200C		YJL200C; J0327 Protein with similarity to aconitase, has potential mitochondrial transit peptide
**155**	YJL213W		YJL213W; HRC331; J0234 Protein with weak similarity to Nocardia aryldialkylphosphatase
**156**	YJL217W		YJL217W; HRC198; J0226 Protein of unknown function
**157**	YJR010W	MET3	MET3; J1436; YJR010W ATP-sulfurylase
**158**	YJR016C	ILV3	ILV3; (ISO1); VAL J1450; YJR016C Dihydroxyacid dehydratase (DAD), third step in valine and isoleucine biosynthesis pathway
**159**	YJR025C	HAD1	BNA1; HAD1; J1550; YJR025C 3-hydroxyanthranilate 3,4-dioxygenase, involved in biosynthesis of nicotinic acid from tryptophan
**160**	YJR026W		
**161**	YJR027W		
**162**	YJR028W		
**163**	YJR029W		
**164**	YJR109C	CPA2	CPA2; J2002; YJR109C Carbamoylphosphate synthase of arginine biosynthetic pathway, synthetase (large) subunit
**165**	YJR111C		YJR111C; J2009 Protein of unknown function
**166**	YJR130C		STR2; J2063; YJR130C Cystathionine gamma-synthase, part of the C3 to C4 transsulfurylation pathway
**167**	YJR137C	ECM17	ECM17; FNR J2126; YJR137C Putative sulfite reductase
**168**	YJR148W	BAT2	BAT2; TWT2; ECA40; J2209; YJR148W Cytosolic branched-chain amino acid transaminase
**169**	YJR154W		YJR154W; J2240 Protein of unknown function
**170**	YJR155W		AAD10; J2245; YJR155W Putative aryl alcohol dehydrogenase, may participates in late steps of degradation of aromatic compounds that arisefrom the degradation of lignocellulose
**171**	YKL001C	MET14	MET14; YKL001C Adenosine-5'-phosphosulfate 3'-phosphotransferase (adenylylsulfate kinase), part of the sulfate assimilation pathway
**172**	YKL023W		YKL023W Protein of unknown function
**173**	YKL072W	STB6	STB6; YKL352; YKL072W Protein which binds to Sin3p
**174**	YKL106W	AAT1	AAT1; YKL461; YKL106W Putative aspartate aminotransferase, mitochondrial
**175**	YKL120W		OAC1; PMT (PMT1); YKL522; YKL120W Mitochondrial oxaloacetate transporter, member of the mitochondrial carrier (MCF) family
**176**	YKL155C		RSM22; RSM50; YKL610; YKL155C Putative component of the mitochondrial ribosomal small subunit
**177**	YKL211C	TRP3	TRP3; YKL211C Anthranilate synthase:indole-3-glycerol phosphate synthase (anthranilate synthase), component II, involved in the first andfourth steps of the tryptophan biosynthesis pathway

**178**	YKL218C		SRY1; YKL218C Pyridoxal 5'phosphate-dependent enzyme, has strong similarity to mouse glial serine racemase and E. coli threonine dehydratase
**179**	YKR015C		YKR015C Protein of unknown function
**180**	YKR036C	CAF4	CAF4; YKR036C Protein that associates with Ccr4p, contains WD (WD-40) repeats
**181**	YKR069W	MET1	MET1; MET20; YKR069W Siroheme synthase involved in methionine metabolism
**182**	YKR071C		YKR071C Protein of unknown function
**183**	YKR080W	MTD1	MTD1; YKR400; YKR080W NAD-dependent 5,10-methylenetetrahydrafolate dehydrogenase
**184**	YKR099W	BAS1	BAS1; YKR099W Transcription factor involved in regulation of basal and induced activity of histidine and adenine biosynthesis genes
**185**	YLL018C	DPS1	DPS1; APS (APS1); ATS APSG L1295; YLL018C Aspartyl-tRNA synthetase, cytoplasmic
**186**	YLL027W		ISA1; L0943; YLL027W Mitochondrial protein required for normal iron metabolism, involved in maturation of mitochondrial and cytosoliciron-sulfur proteins
**187**	YLR027C	AAT2	AAT2; (AAT1); ASP5; L1746; YLR027C Aspartate aminotransferase (2-oxoglutarate aminotransferase), cytosolic and peroxisomal
**188**	YLR058C	SHM2	SHM2; SHMT2; L2156; YLR058C Serine hydroxymethyltransferase (glycine hydroxymethyltransferase), cytosolic isoform, catalyzes the transferof the hydroxymethyl group of serine to tetrahydrofolate to form 5,10-methylenetetrahydrofolate and glycine
**189**	YLR089C		YLR089C; L9449.15; L2518 Putative mitochondrial alanine aminotransferase
**190**	YLR092W	SUL2	SUL2; (SEL2); L9449.1; L2528; YLR092W High-affinity sulfate transporter, member of the sulfate permease family of membrane transporters
**191**	YLR152C		YLR152C; L9634.9; L3329 Protein with similarity to Ecm3p
**192**	YLR193C		YLR193C; L8167.12 Protein of unknown function, has low similarity to Ydr185p and Ylr168p
**193**	YLR256W	HAP1	HAP1; (CYP1); L9672.1; YLR256W Transcription factor with heme-dependent DNA-binding activity, responsible for heme-dependent activationof many genes
**194**	YLR266C		YLR266C; L8479.13 Protein with similarity to transcription factors, has Zn[2]-Cys[6] fungal-type binuclear cluster domain in the N-terminal region
**195**	YLR267W		BOP2; L8479.4; YLR267W Protein of unknown function
**196**	YLR290C		YLR290C; L8003.16 Protein of unknown function
**197**	YLR302C		YLR302C Protein of unknown function
**198**	YLR303W	MET17	MET17; MET25; MET15; L8003.1; YLR303W O-acetylhomoserine sulfhydrylase (OAH SHLase) converts O-acetylhomoserine intohomocysteine
**199**	YLR334C		YLR334C; L8300.11 Protein of unknown function
**200**	YLR343W		YLR343W; L8300.5; GAS2 Protein with strong similarity to Gas1p, member of the GPI-anchored beta(1-3)glucanosyltransferases family
**201**	YLR355C	ILV5	ILV5; ILV5X; L9638.7; YLR355C Ketol-acid reductoisomerase (acetohydroxy-acid reductoisomerase, alpha-keto-beta-hydroxylacilreductoisomerase), second step in valine and isoleucine biosynthesis pathway

**202**	YLR387C		YLR387C; L3502.9 Protein possibly involved in proteasome function, has three dispersed C2H2-type zinc fingers
**203**	YML039W		
**204**	YML040W		
**205**	YML045W		
**206**	YML097C	VPS9	VPS9; VPT9; VPL31; YML097C Protein involved in vacuolar sorting
**207**	YML116W	ATR1	ATR1; SNQ1; M_C542 YM8339.03; YML116W Membrane transporter conferring aminotriazole and 4-nitroquinoline-1-oxide (4NQO) resistance,member of the multidrug-resistance 14-spanner (DHA14) family of the major facilitator superfamily (MFS-MDR)
**208**	YMR042W	ARG80	ARG80; ARGR1; ARGRI YM9532.07; YMR042W Component of the ARGR transcription regulatory complex
**209**	YMR045C		
**210**	YMR046C		
**211**	YMR050C		
**212**	YMR051C		
**213**	YMR062C	ECM40	ARG7; argE ECM40; YM9916.01; YMR062C Ornithine acetyltransferase catalyzes the fifth step in ornithine and arginine biosynthesis, also hasacetylglutamate synthase activity
**214**	YMR094W	CTF13	CTF13; CBF3C; YM9582.19; YM6543.01; YMR094W Component (subunit c) of Cbf3 kinetochore complex, contains an F-box domain
**215**	YMR095C	SNO1	SNO1; YM6543.02; YMR095C Putative pyridoxine (vitamin B6) biosynthetic enzyme with similarity to glutamine aminotransferases, has strongsimilarity to Sno1p and Sno3p
**216**	YMR096W	SNZ1	SNZ1; YM6543.03; YMR096W Putative pyridoxine (vitamin B6) biosynthetic enzyme, member of the stationary phase-induced gene family whichincludes Snz2p and Snz3p
**217**	YMR097C		YMR097C; YM6543.04 Protein of unknown function
**218**	YMR108W	ILV2	ILV2; SMR1; YM9718.07; YMR108W Acetolactate synthase (acetohydroxyacid synthase), first step in valine and isoleucine biosynthesispathway
**219**	YMR120C	ADE17	ADE17; YM8564.02; YMR120C 5-aminoimidazole-4-carboxamide ribonucleotide (AICAR) transformylase/IMP cyclohydrolase
**220**	YMR189W	GCV2	GCV2; GSD2; YM9646.01; YMR189W Glycine decarboxylase pyridoxal phosphate containing P subunit (glycine cleavage P protein), functions inthe pathway for glycine degradation
**221**	YMR195W		ICY1; YM9646.08; YMR195W Protein of unknown function, interacts with the cytoskeleton
**222**	YMR271C	URA10	URA10; YM8156.13; YMR271C Orotate phosphoribosyltransferase 2, fifth step in pyrimidine biosynthesis pathway
**223**	YMR300C	ADE4	ADE4; PUR6; YM9952.02; YMR300C Amidophosphoribosyltransferase (glutamine phosphoribosylpyrophosphate amidotransferase) catalyzesthe first step in de novo purine biosynthesis
**224**	YMR321C		YMR321C; YM9924.13 Protein nearly identical to a fragment of Sam4p, possibly a pseudogene
**225**	YNL004W	HRB1	HRB1; TOM34; N2009; YNL004W Protein with similarity to Rlf6p, contains three RNA recognition motif (RRM) domains
**226**	YNL036W	NCE3	NCE103; NCE3; N2695; YNL036W Protein involved in protection against oxidative damage

**227**	YNL103W	MET4	MET4; N2177; YNL103W Transcriptional activator of the sulfur assimilation pathway member of basic leucine zipper (bZIP) family
**228**	YNL104C	LEU4	LEU4; TFL1; N2173; YNL104C 2-Isopropylmalate synthase, first step in leucine biosynthesis pathway
**229**	YNL129W		YNL129W; N1219; N1870 Protein of unknown function
**230**	YNL220W	ADE12	ADE12; BRA9; N1290; YNL220W Adenylosuccinate synthetase, carries out addition of aspartic acid to IMP with GTP hydrolysis
**231**	YNL221C	POP1	POP1; N1285; YNL221C Protein component of both RNase P and RNase MRP, involved in both tRNA maturation (RNase P) and in 5.8S rRNAprocessing (RNase MRP)
**232**	YNL229C	URE2	URE2; N1165; YNL229C Regulator of nitrogen catabolite repression, acts by inhibition of the Gln3p regulator in the presence of preferred nitrogensources, forms [URE3] prion
**233**	YNL239W	LAP3	LAP3; YCP1; BLH1; GAL6; N1118; YNL239W Aminopeptidase of cysteine protease family, homologous to rabbit bleomycin hydrolase
**234**	YNL276C		YNL276C; N0621 Protein of unknown function, questionable ORF
**235**	YNL311C		YNL311C; N0376 Protein of unknown function, may be involved in amino acid metabolism contains an cyclin-associated F-box
**236**	YNR050C	LYS9	LYS9; LYS13; ly9; N3461; YNR050C Saccharopine dehydrogenase (saccharopine reductase NADP+, L-glutamate forming), seventh step inlysine biosynthesis pathway
**237**	YNR056C	BIO5	BIO5; N3502; YNR056C Transmembrane regulator of KAPA/DAPA transport, involved in biotin biosynthesis
**238**	YNR057C	BIO4	BIO4; N3506; YNR057C Dethiobiotin synthase, component of the biotin biosynthesis pathway
**239**	YNR058W	BIO3	BIO3; N3510; YNR058W DAPA aminotransferase, component of the biotin biosynthesis pathway
**240**	YNR065C		YNR065C; N3539 Protein with similarity to Pep1p
**241**	YNR068C		YNR068C; N3551 Protein with similarity to Bul1p ubiquitin ligase binding protein, may be a pseudogene or separated from YNR069C bysequencing errors
**242**	YNR069C		YNR068C; N3551 Protein with similarity to Bul1p ubiquitin ligase binding protein, may be a pseudogene or separated from YNR069C bysequencing errors
**243**	YNR074C		YNR074C; N3815 Protein with similarity to various reductases
**244**	YOL058W	ARG1	ARG1; ARG10; argG O1228; YOL058W Argininosuccinate synthetase (citrulline--aspartate ligase) catalyzes the penultimate step in argininesynthesis
**245**	YOL059W	GPD2	GPD2; (GPD3); O1222; YOL059W Glycerol-3-phosphate dehydrogenase (NAD+) involved in glycerol production convertingglycerol-3-phosphate and NAD+ to dihydroxyacetone phosphate and NADH
**246**	YOL064C	MET22	MET22; HAL2; O1180; YOL064C 3'- (2'-), 5'- Bisphosphate nucleotidase required for sulfate assimilation, involved in salt tolerance and methioninebiogenesis
**247**	YOL091W		SPO21; MPC70; O0932; YOL091W Component of the meiotic plaque, required for formation of the prospore membrane
**248**	YOL106W		YOL106W; O0751 Protein of unknown function
**249**	YOL118C		YOL118C; O0573 Protein of unknown function

**250**	YOL119C		YOL119C; O0569 Protein with weak similarity to mammalian monocarboxylate transporter proteins, member of the monocarboxylate porter (MCP)family of the major facilitator superfamily (MFS)
**251**	YOL140W	ARG8	ARG8; argD AOE423; O0477; YOL140W Acetylornithine aminotransferase catalyzes the fourth step in ornithine and arginine biosynthesis
**252**	YOR044W		YOR044W; O2771 Protein of unknown function
**253**	YOR108W		YOR108W; LEU9; YOR3227; O3227 Alpha-isopropylmalate synthase II
**254**	YOR130C	ARG11	ORT1; ARG11; O3299; YOR3299; YOR130C Ornithine transport protein of mitochondria involved in arginine metabolism, member of themitochondrial carrier (MCF) family
**255**	YOR184W	SER1	SER1; SERC ADE9; O4731; YOR184W 3-phosphoserine transaminase, involved in synthesis of serine from 3-phosphoglycerate
**256**	YOR200W		YOR200W; O4824 Protein of unknown function
**257**	YOR202W	HIS3	HIS3; HIS8; HIS10; O4830; YOR202W Imidazoleglycerolphosphate (IGP) dehydratase, seventh step in histidine biosynthesis pathway
**258**	YOR203W		YOR203W; O4833 Protein of unknown function
**259**	YOR221C		MCT1; YOR50-11; O5058; O5011; YOR221C Malonyl CoA:acyl carrier protein transferase
**260**	YOR222W		ODC2; PTP YOR50-12; O5012; O5064; YOR222W 2-oxodicarboxylate transporter, specific for 2-oxoadipate and 2-oxoglutarate member of themitochondrial carrier family (MCF) of membrane transporters
**261**	YOR226C		ISU2; NUA2; YOR50-16; O5016; O5076; YOR226C Protein with similarity to iron-sulfur cluster nitrogen fixation proteins
**262**	YOR230W	WTM1	WTM1; YOR50-20; O5020; O5091; YOR230W Transcriptional modulator involved in meiotic regulation and silencing
**263**	YOR302W		
**264**	YOR303W	CPA1	CPA1; O5645; YOR303W Carbamoylphosphate synthase of the arginine biosynthetic pathway, amidotransferase small subunit
**265**	YOR306C		YOR306C; O5658 Protein with similarity to human X-linked PEST-containing transporter, member of the monocarboxylate porter (MCP) family ofthe major facilitator superfamily (MFS)
**266**	YOR323C	PRO2	PRO2; SOG1; O6155; YOR323C Gamma-glutamyl phosphate reductase (phosphoglutamate dehydrogenase), proline biosynthetic enzyme
**267**	YOR337W	TEA1	TEA1; O6257; YOR337W Ty1 enhancer activator of the Gal4p-type family of DNA-binding proteins
**268**	YOR339C		UBC11; O6268; YOR339C Protein with similarity to ubiquitin-conjugating enzymes
**269**	YOR363C	PIP2	PIP2; (OAF2); O6655; YOR363C Protein involved in induction of peroxisomal proteins in response to oleic acid
**270**	YPL033C		YPL033C; P7102.16 Protein of unknown function
**271**	YPL092W	SSU1	SSU1; (SUR1); LPG16; YPL092W Plasma membrane transporter involved in sulfite efflux
**272**	YPL135W		ISU1; NUA1; LPI10; YPL135W Protein with similarity to iron-sulfur cluster nitrogen fixation proteins
**273**	YPL140C	MKK2	MKK2; SSP33; LPI6; YPL140C MAP kinase kinase (MEK) serine/threonine protein kinase involved in cell wall integrity (low-osmolarity)pathway
**274**	YPL188W	POS5	POS5; P2205; YPL188W Protein involved in sensitivity to peroxide, has similarity to Utr1p and Yel041p

**275**	YPL212C	PUS1	PUS1; P1805; YPL212C Pseudouridine synthase responsible for specific pseudouridine modifications in tRNA and in U2 snRNA
**276**	YPL250C		ICY2; P1013; YPL250C Protein of unknown function, transcription induced by the drug FK506 in a GCN4-dependent manner
**277**	YPL251W		YPL251W; P1010 Protein of unknown function
**278**	YPL252C		YAH1; P1007; YPL252C Protein with similarity to adrenodoxin and ferrodoxin
**279**	YPL264C		YPL264C; P0373 Protein of unknown function
**280**	YPL273W		SAM4; P0338; YPL273W Putative cobalamin-dependent homocysteine S-methyltransferase
**281**	YPR058W	YMC1	YMC1; YP9499.14; YPR058W Member of the mitochondrial carrier (MCF) family of membrane transporters
**282**	YPR059C		YPR059C Protein of unknown function
**283**	YPR145W	ASN1	ASN1; ASNA P9659.3; YPR145W Asparagine synthetase (L-aspartate: L-glutamine amidoligase [AMP-forming]) Asn1p and Asn2p areisozymes
**284**	YPR167C	MET16	MET16; P9325.8; YPR167C 3'-Phosphoadenylylsulfate reductase (PAPS), part of the sulfate assimilation pathway
**285**	YHR214CB		
**286**	YBR012WB		
**287**	YBL101WB		
**288**	YBR012WA		
**289**	YBL005WB		
**290**	YBL005WA		
**291**	YCL026CA		FRM2; YCLX8C; YCLX08C; YCL026C-A Protein involved in the integration of lipid signaling pathways with cellular homeostasis
**292**	YMR173WA		YMR173W-A Probable membrane protein of unknown function
**293**	YBL107WA		
**294**	YLR035CA		YLR035C-A Protein of unknown function
**295**	YGR122CA		YGR122C-A Protein of unknown function
**296**	YPR002CA		YPR002C-A Protein of unknown function, has similarity to Ylr334p and Yol106p
**297**	YMR046WA		YMR046W-A Protein of unknown function, has similarity to Ylr334p and Yol106p
**298**	YDR170WA		YDR170W-A Protein of unknown function
**299**	YFL002WA		
**300**	YDR525WA		YDR525W-A Protein of unknown function, has similarity to Yjl151p and Ydl123p
**301**	YCR018CA		YCR018C-A Protein of unknown function, has similarity to Ylr334p and Yol106p
**302**	YMR158CB		YMR158C-B Protein of unknown function, has similarity to Hap1p and Ydr340p
**303**	YKL033WA		YKL033W-A Protein with strong similarity to holacid-halidohydrolase

Iron transport genes

Serial #	ORF	Name	description
**1**	YAL061W		YAL061W; FUN50 Protein with similarity to alcohol/sorbitol dehydrogenase member of the zinc-containing alcohol dehydrogenase family
**2**	YBR047W		YBR047W; YBR0422 Protein of unknown function transcription induced during aerobic growth, also induced by the drug FK506 in aGCN4-dependent manner
**3**	YBR207W		FTH1; YBR1448; YBR207W Vacuolar iron transporter with similarity to Ftr1p
**4**	YBR295W	PCA1	PCA1; PAY2; CAD2; YBR2112; YBR295W P-type copper-transporting ATPase, involved in resistance to cadmium
**5**	YDR264C	AKR1	AKR1; YD9230B.03; D9954.9; YDR264C Ankyrin repeat-containing protein has an inhibitory effect on signaling in the pheromone pathway
**6**	YDR269C		YDR269C Protein of unknown function, questionable ORF
**7**	YDR270W	CCC2	CCC2; D9954.6; YDR270W Copper-transporting P-type ATPase, member of the cation transport (E1-E2) ATPase family
**8**	YDR271C		YDR271C Protein of unknown function, questionable ORF
**9**	YDR406W	PDR15	PDR15; D9509.24; YDR406W Member of ATP-binding cassette (ABC) superfamily
**10**	YDR534C		YDR534C; D9719.37 Protein possibly involved in iron uptake
**11**	YEL065W	SIT1	SIT1; ARN3; YEL065W Ferrioxamine B permease, member of the yeast-specific multidrug-resistance (MFS-MDR) family of the major facilitatorsuperfamily (MFS)
**12**	YER145C	FTR1	FTR1; YER145C Iron permease that mediates high-affinity iron uptake
**13**	YER175C		YER175C; SYGP-ORF63 Protein with predicted S-adenosylmethionine-dependent methyltransferase motif, has similarity to Yhr209p; transcriptioninduced by the drug FK506 in a GCN4-dependent manner
**14**	YFL041W		FET5; YFL041W Multicopper oxidase involved in ferrous iron transport
**15**	YFR024C		YFR024C; LSB3 Protein with possible role in the regulation of actin cytoskeletal organization, has similarity to Ysc84p, Rvs167p, Abp1p, andSla1p, has an SH3 domain
**16**	YGL156W	AMS1	AMS1; G1861; YGL156W alpha-Mannosidase, hydrolyzes terminal non-reducing alpha-D-mannose residues from alpha-D-mannosides
**17**	YGR035C		YGR035C; G4082 Protein of unknown function
**18**	YGR065C		VHT1; G4539; YGR065C H+/biotin symporter and member of the allantoate permease family of the major facilitator superfamily (MFS)
**19**	YGR257C		YGR257C; G9175 Member of the mitochondrial carrier family (MCF) of membrane transporters
**20**	YHL035C		YHL035C Member of the ATP-binding cassette (ABC) superfamily
**21**	YHL040C		ARN1; YHL040C Protein involved in the transport of ferrichromes, member of the yeast-specific putative multidrug-resistance family of the majorfacilitator superfamily (MFS)
**22**	YHL047C		TAF1; ARN2; YHL047C Triacetylfusarinine C transporter, member of the yeast-specific putative multidrug-resistance family of the major facilitatorsuperfamily (MFS)
**23**	YHR175W	CTR2	CTR2; YHR175W Putative low-affinity copper transport protein
**24**	YKL220C	FRE2	FRE2; F711; YKL220C Ferric and cupric reductase, paralog of Fre1p subject to regulation by iron

**25**	YKR052C	MRS4	MRS4; YKR052C Splicing protein and member of the mitochondrial carrier (MCF) family, suppresses mitochondrial splicing defects
**26**	YKR076W	ECM4	ECM4; YKR076W Protein possibly involved in cell wall structure or biosynthesis
**27**	YLL051C	FRE6	FRE6; L0593; YLL051C Protein with similarity to ferric reductase Fre2p, subject to regulation by iron
**28**	YLR034C		SMF3; L1779; YLR034C Possible metal transporter, has similarity to Smf1p and Smf2p
**29**	YLR046C		YLR046C; L2111 Protein with similarity to Rtm1p
**30**	YLR126C		YLR126C; L9233.12; L3105 Protein with weak similarity to Pseudomonas aeroginosa anthranilate synthase component II
**31**	YLR127C	RSI1	APC2; RSI1; TID2; L9233.13; L3108; YLR127C Component of the anaphase-promoting complex, required for Clb2p degradation and for themetaphase-anaphase transition
**32**	YLR136C	TIS11	TIS11; YTIS11; CTH2; L3143; L9606.12; YLR136C Protein of the inducible CCCH zinc finger family
**33**	YLR214W	FRE1	FRE1; L8167.2; YLR214W Membrane-associated flavocytochrome with ferric and cupric reductase activities acts on ferric iron chelates externalto the cell to facilitate iron uptake
**34**	YLR346C		YLR346C; L8300.14 Protein of unknown function
**35**	YMR058W	FET3	FET3; YM9796.11; YMR058W Cell surface ferroxidase required for high-affinity ferrous iron uptake
**36**	YMR102C		YMR102C Protein of unknown function, contains WD (WD-40) repeats
**37**	YMR251W		YMR251W; YM9920.05 Putative paralog of Ecm4p
**38**	YNL037C	IDH1	IDH1; N2690; YNL037C Isocitrate dehydrogenase (NAD+) subunit 1, mitochondrial, required for oxidative function of the tricarboxylic acid cycle
**39**	YNL237W	YTP1	YTP1; N1129; YNL237W Protein with similarity to mitochondrial electron transport proteins
**40**	YNL259C	ATX1	ATX1; N0840; YNL259C Antioxidant protein and metal homeostasis factor functions in a copper trafficking pathway
**41**	YNR056C	BIO5	BIO5; N3502; YNR056C Transmembrane regulator of KAPA/DAPA transport, involved in biotin biosynthesis
**42**	YOL158C		ENB1; ARN4; O0270; YOL158C Protein involved in iron uptake via a siderophore enterobactin member of the yeast-specific putativemultidrug-resistance family of the major facilitator superfamily (MFS)
**43**	YOR049C		YOR049C; O2787 Protein of unknown function
**44**	YOR135C		YOR135C; O3323 Protein of unknown function
**45**	YOR136W	IDH2	IDH2; O3326; YOR3326; YOR136W Isocitrate dehydrogenase (NAD+) subunit 2, mitochondrial, required for oxidative function of thetricarboxylic acid cycle
**46**	YOR152C		YOR152C; O3536 Protein of unknown function
**47**	YOR153W	PDR5	PDR5; (STS1); LEM1; (YDR1); O3542; YOR153W Drug-efflux pump involved in resistance to multiple drugs, member of the ATP-bindingcassette (ABC) superfamily
**48**	YOR316C	COT1	COT1; O6131; YOR316C Protein involved in cobalt accumulation
**49**	YOR381W	FRE3	FRE3; O6754; YOR381W Protein involved in the uptake of iron from siderophores rhodotorulic acid, triacetylfusarinine C, ferrichrome andferrioximine B
**50**	YOR382W		YOR382W; O6760 Protein possibly involved in iron uptake
**51**	YOR383C		YOR383C; O6762 Protein possibly involved in iron uptake

**52**	YOR384W	FRE5	FRE5; O6765; YOR384W Protein with similarity to Fre2p, subject to regulation by iron
**53**	YPL135W		ISU1; NUA1; LPI10; YPL135W Protein with similarity to iron-sulfur cluster nitrogen fixation proteins
**54**	YPL154C	PEP4	PEP4; PRA1; PHO9; P2585; YPL154C Proteinase A (PrA/yscA/saccharopepsin) aspartyl protease required for activation of various degradativeenzymes

**PAU genes**			
**Serial #**	**ORF**	**Name**	**description**
**1**	YAL068C		YAL068C Member of the seripauperin (PAU) family
**2**	YBR005W		YBR005W; YBR0111 Protein of unknown function, mRNA abundance is reduced by the drug FK506 in a calcineurin- and immunophilin-dependentmanner
**3**	YBR301W		YBR301W; YBR2120A Member of the seripauperin (PAU) family
**4**	YCR104W	PAU3	PAU3; YCR104W Member of the seripauperin (PAU) family
**5**	YDL234C	GYP7	GYP7; D0802; YDL234C GTPase-activating protein for Ypt7p
**6**	YDR391C		YDR391C; D9509.11 Protein of unknown function
**7**	YDR542W		YDR542W; D3703.3 Member of the seripauperin (PAU) family
**8**	YEL049W	PAU2	PAU2; SYGP-ORF12; YEL049W Member of the seripauperin (PAU) family
**9**	YEL060C	PRB1	PRB1; CVT1; YEL060C Protease B (yscB/PrB/cerevisin), serine protease of the subtilisin family with broad proteolytic specificity
**10**	YER158C		YER158C Protein with similarity to Afr1p
**11**	YFL020C	PAU5	PAU5; YFL020C Member of the seripauperin (PAU) family
**12**	YGL156W	AMS1	AMS1; G1861; YGL156W alpha-Mannosidase, hydrolyzes terminal non-reducing alpha-D-mannose residues from alpha-D-mannosides
**13**	YGL165C		YGL165C; G1814 Protein of unknown function
**14**	YGL261C		YGL261C; NRF120; NRB120; G0328 Member of the seripauperin (PAU) family
**15**	YGR032W	GSC2	GSC2; FKS2; (GLS2); G4074; YGR032W Component of beta-1,3-glucan synthase, probably functions as an alternate subunit with Fks1p withwhich is has strong similarity
**16**	YGR161C		YGR161C; G7008 Protein of unknown function, has phosphopantetheine attachment site
**17**	YGR213C	RTA1	RTA1; G7811; YGR213C Protein involved in 7-aminocholesterol resistance
**18**	YGR294W		YGR294W; G9599 Member of the seripauperin (PAU) family
**19**	YHL046C		YHL046C Member of the seripauperin (PAU) family
**20**	YHR030C	SLT2	SLT2; MPK1; SLK2; BYC2; CRV3; TSL6; LYT2; SIW9; YHR030C Serine/threonine protein kinase of MAP kinase family involved in the cell wallintegrity (low-osmolarity) pathway
**21**	YHR097C		YHR097C Protein of unknown function
**22**	YHR138C		YHR138C Protein possibly involved in vacuolar fusion, has similarity to Pbi2p, which is a protease B inhibitor
**23**	YHR209W		YHR209W Putative SAM-dependent methyltransferase has similarity to Yer175p

**24**	YIL176C		YIL176C; YI9402.02 Member of the seripauperin (PAU) family (PAU1 and YIL176C code for identical proteins)
**25**	YIR041W		YIR041W; YI8224.03 Member of the seripauperin (PAU) family
**26**	YJL151C		YJL151C; J0630 Protein of unknown function, has similarity to Ydr525W-Ap and Ydl123p
**27**	YJL171C		YJL171C; J0512 Protein of unknown function
**28**	YJL223C		PAU1; J0208; HRD120; YJL223C Member of the seripauperin (PAU) family (PAU1 and YIL176C code for identical proteins)
**29**	YKL065C	YET1	YET1; YKL331; YKL065C Transmembrane protein of the endoplasmic reticulum
**30**	YKL142W	MRP8	MRP8; YKL3; YKL142W Mitochondrial ribosomal protein of the small subunit
**31**	YKL224C		YKL224C; D123 Member of the seripauperin (PAU) family subtelomerically encoded
**32**	YKR091W	SRL3	SRL3; YKR411; YKR091W Protein of unknown function
**33**	YLL025W		YLL025W; L0968 Member of the seripauperin (PAU) family
**34**	YLL064C		YLL064C; L0543 Member of the seripauperin (PAU) family (YLL064C and PAU6 code for identical proteins)
**35**	YLR037C		YLR037C; L1909 Member of the seripauperin (PAU) family
**36**	YLR120C	YAP3	YPS1; (YAP3); L9233.9; L2961; YLR120C Yapsin 1, GPI-anchored aspartyl protease that cleaves C-terminal to paired basic residues(Aspergillopepsin I)
**37**	YLR121C		YPS3; (YPS4); L9233.10; L2964; YLR121C GPI-anchored aspartyl protease
**38**	YLR194C		YLR194C; L8167.13 Protein of unknown function
**39**	YLR327C		YLR327C; L8543.1 Protein with strong similarity to Stf2p
**40**	YLR414C		YLR414C; L9931.8 Protein of unknown function
**41**	YLR461W	PAU4	PAU4; L9122.1; YLR461W Member of the seripauperin (PAU) family
**42**	YMR007W		YMR007W; YM8270.09 Protein involved in tunicamycin sensitivity
**43**	YMR008C	PLB1	PLB1; YM8270.10; YMR008C Phospholipase B, preferentially deacylates phosphatidylcholine and phosphatidylethanolamine
**44**	YMR020W	FMS1	FMS1; YM9711.09; YMR020W Protein involved in the biosynthesis of pantothenic acid, has similarity to Candida albicans corticosteroid-bindingprotein CBP1
**45**	YMR040W		YMR040W; YM9532.05 Protein with similarity to Ykl065p
**46**	YMR181C		YMR181C; YM8010.11 Protein of unknown function
**47**	YMR316W		DIA1; YM9924.08; YMR316W Protein of unknown function containing a zinc carboxypeptidase motif PS00133; mRNA abundance is reduced bythe drug FK506 in a calcineurin- and immunophilin-dependent manner
**48**	YMR325W		YMR325W; YM9924.17 Member of the seripauperin (PAU) family
**49**	YNL208W		YNL208W; N1338 Protein of unknown function
**50**	YNR059W		MNT4; N3514; YNR059W Protein with strong similarity to mannosyltransferases Mnn1p, Mnt2p and Mnt3p
**51**	YNR076W	PAU6	PAU6; N3825; YNR076W Member of the seripauperin (PAU) family (YLL064C and PAU6 code for identical proteins)
**52**	YOL016C	CMK2	CMK2; O2325; YOL016C Calcium/calmodulin-dependent serine/threonine protein kinase (CaM kinase) type II
**53**	YOL161C		YOL161C; O0240 Member of the seripauperin (PAU) family subtelomerically-encoded

**54**	YOR036W	PEP12	PEP12; VPL6; VPT13; VPS6; OR26.29; O2750; YOR036W Syntaxin homolog (t-SNARE) involved in Golgi to vacuole transport
**55**	YOR134W	BAG7	BAG7; YOR3320; O3320; YOR134W Putative GTPase-activating protein (GAP)
**56**	YOR208W	PTP2	PTP2; O4849; YOR208W Protein tyrosine phosphatase (PTPase), involved in Hog1p MAP kinase high-osmolarity sensing pathway and Fus3pMAP kinase pheromone response pathway
**57**	YOR220W		YOR220W; YOR50-10; O5010; O5050 Protein of unknown function
**58**	YOR289W		YOR289W; O5498W Protein of unknown function
**59**	YOR385W		YOR385W; O6768 Protein of unknown function
**60**	YOR394W		YOR394W; O7527; (ORF2) Member of the seripauperin (PAU) family (YPL282C and YOR394W code for identical proteins)
**61**	YPL088W		YPL088W; LPG20 Putative aryl alcohol dehydrogenase, may participate in late steps of degradation of aromatic compounds that arise from thedegradation of lignocellulose
**62**	YPL282C		YPL282C; P0301 Member of the seripauperin (PAU) family (YPL282C and YOR394W code for identical proteins)
**63**	YPR154W		YPR154W; LSB2; P9584.5 Protein with one SH3 domain, has similarity to myosin ID and IC heavy chains, human growth factor receptor-boundgrb2 protein, C. elegans sex muscle abnormal protein 5
**64**	YMR316CA		YMR316C-A Protein of unknown function

**Phosphate + iron utilization genes**			
**Serial #**	**ORF**	**Name**	**description**
**1**	YAR071W	PHO11	PHO11; YAR071W Acid phosphatase, secreted
**2**	YBR092C	PHO3	PHO3; YBR0813; YBR092C Acid phosphatase, constitutive, thiamine-binding protein of the periplasmic space
**3**	YBR093C	PHO5	PHO5; YBR0814; YBR093C Acid phosphatase, repressible, requires glycosylation for activity
**4**	YBR296C		PHO89; ITN1; YBR2113; YBR296C High-affinity Na+-dependent phosphate transporter
**5**	YDL039C	PRM7	PRM7; D2723; YDL039C Protein of unknown function
**6**	YDR264C	AKR1	AKR1; YD9230B.03; D9954.9; YDR264C Ankyrin repeat-containing protein has an inhibitory effect on signaling in the pheromone pathway
**7**	YDR270W	CCC2	CCC2; D9954.6; YDR270W Copper-transporting P-type ATPase, member of the cation transport (E1-E2) ATPase family
**8**	YDR281C		PHM6; D9954.14; YDR281C Protein of unknown function, has a role in phosphate metabolism
**9**	YDR481C	PHO8	PHO8; D8035.24; YDR481C Vacuolar alkaline phosphatase (ALP), repressible, carries out dephosphorylation of phosphopeptides
**10**	YDR534C		YDR534C; D9719.37 Protein possibly involved in iron uptake
**11**	YEL065W	SIT1	SIT1; ARN3; YEL065W Ferrioxamine B permease, member of the yeast-specific multidrug-resistance (MFS-MDR) family of the major facilitatorsuperfamily (MFS)
**12**	YER072W		VTC1; NRF1; YER072W Protein involved in the distribution of V-ATPase and other membrane proteins

**13**	YER145C	FTR1	FTR1; YER145C Iron permease that mediates high-affinity iron uptake
**14**	YFL004W		VTC2; PHM1; YFL004W Putative polyphosphate synthetase
**15**	YGR035C		YGR035C; G4082 Protein of unknown function
**16**	YGR233C	PHO81	PHO81; (SPL1); ORF1178; G8567; YGR233C Cyclin-dependent kinase (CDK) inhibitor for Pho80p-Pho85p cyclin-dependent protein kinasecomplex, positive regulator of phosphate pathway
**17**	YGR234W	YHB1	YHB1; YHB ORF399; G8572; YGR234W Flavohemoglobin involved in protection from nitrosative stress, distantly related to animal hemoglobins
**18**	YHL040C		ARN1; YHL040C Protein involved in the transport of ferrichromes, member of the yeast-specific putative multidrug-resistance family of the majorfacilitator superfamily (MFS)
**19**	YHL047C		TAF1; ARN2; YHL047C Triacetylfusarinine C transporter, member of the yeast-specific putative multidrug-resistance family of the major facilitatorsuperfamily (MFS)
**20**	YHR136C	SPL2	SPL2; YHR136C Putative inhibitor of Pho80p-Pho85p cyclin-dependent protein kinase, may block phosphorylation of novel factors other thanPho4p
**21**	YHR215W	PHO12	PHO12; PHO10; H9177.3; YHR215W Acid phosphatase, secreted
**22**	YJL012C		VTC4; PHM3; J1345; YJL012C Putative polyphosphate synthetase
**23**	YJL117W	PHO86	PHO86; J0744; YJL117W Protein associated with phosphate transport complex, regulates Pho81p
**24**	YJR151C		YJR151C; J2223 Member of the seripauperin (PAU) family of possible cell wall mannoproteins
**25**	YLL051C	FRE6	FRE6; L0593; YLL051C Protein with similarity to ferric reductase Fre2p, subject to regulation by iron
**26**	YLR056W	ERG3	ERG3; SYR1; PSO6; NYS3; L2150; YLR056W C-5 sterol desaturase, an iron, non-heme, oxygen-requiring enzyme of the ergosterol biosynthesispathway
**27**	YLR214W	FRE1	FRE1; L8167.2; YLR214W Membrane-associated flavocytochrome with ferric and cupric reductase activities acts on ferric iron chelates externalto the cell to facilitate iron uptake
**28**	YLR410W		VIP1; L9931.7; YLR410W Protein with possible methionine N(alpha)-acetyltransferase activity involved in cortical actin function
**29**	YML123C	PHO84	PHO84; YM7056.03; YML123C High-affinity inorganic phosphate/H+ symporter, member of the phosphate:H[+] symporter (PHS) family of themajor facilitator superfamily (MFS)
**30**	YMR058W	FET3	FET3; YM9796.11; YMR058W Cell surface ferroxidase required for high-affinity ferrous iron uptake
**31**	YMR251W		YMR251W; YM9920.05 Putative paralog of Ecm4p
**32**	YNL217W		YNL217W; N1306 Protein possibly involved in cytoplasmic ribosome function
**33**	YNL259C	ATX1	ATX1; N0840; YNL259C Antioxidant protein and metal homeostasis factor functions in a copper trafficking pathway
**34**	YOL130W	ALR1	ALR1; O0522; YOL130W Putative cytoplasmic magnesium and cobalt transporter, member of the metal ion transporter family of membranetransporters
**35**	YOL158C		ENB1; ARN4; O0270; YOL158C Protein involved in iron uptake via a siderophore enterobactin member of the yeast-specific putativemultidrug-resistance family of the major facilitator superfamily (MFS)
**36**	YOR153W	PDR5	PDR5; (STS1); LEM1; (YDR1); O3542; YOR153W Drug-efflux pump involved in resistance to multiple drugs, member of the ATP-bindingcassette (ABC) superfamily

**37**	YOR382W		YOR382W; O6760 Protein possibly involved in iron uptake
**38**	YOR383C		YOR383C; O6762 Protein possibly involved in iron uptake
**39**	YPL017C		YPL017C; LPB14 Protein with similarity to Lpd1p
**40**	YPL018W	CTF19	CTF19; LPB13; YPL018W Protein important for chromosome segregation
**41**	YPL019C		VTC3; PHM2; LPB12; YPL019C Putative polyphosphate synthetase
**42**	YPL110C		YPL110C; LPH16 Protein with similarity to Pho81p

**Calcium calmodulin genes**			
**Serial #**	**ORF**	**Name**	**description**
**1**	YBR005W		YBR005W; YBR0111 Protein of unknown function, mRNA abundance is reduced by the drug FK506 in a calcineurin- and immunophilin-dependentmanner
**2**	YDL234C	GYP7	GYP7; D0802; YDL234C GTPase-activating protein for Ypt7p
**3**	YDR001C		NTH1; NTH YD8119.07; D2955; YDR001C Neutral trehalase
**4**	YDR391C		YDR391C; D9509.11 Protein of unknown function
**5**	YEL060C	PRB1	PRB1; CVT1; YEL060C Protease B (yscB/PrB/cerevisin), serine protease of the subtilisin family with broad proteolytic specificity
**6**	YGR136W		YGR136W; LSB1; G6409 Protein with weak similarity to chicken growth factor receptor-binding protein, has an SH3 domain
**7**	YGR213C	RTA1	RTA1; G7811; YGR213C Protein involved in 7-aminocholesterol resistance
**8**	YHL021C		YHL021C Protein of unknown function
**9**	YHR030C	SLT2	SLT2; MPK1; SLK2; BYC2; CRV3; TSL6; LYT2; SIW9; YHR030C Serine/threonine protein kinase of MAP kinase family involved in the cell wallintegrity (low-osmolarity) pathway
**10**	YHR097C		YHR097C Protein of unknown function
**11**	YHR138C		YHR138C Protein possibly involved in vacuolar fusion, has similarity to Pbi2p, which is a protease B inhibitor
**12**	YHR209W		YHR209W Putative SAM-dependent methyltransferase has similarity to Yer175p
**13**	YJL171C		YJL171C; J0512 Protein of unknown function
**14**	YKL065C	YET1	YET1; YKL331; YKL065C Transmembrane protein of the endoplasmic reticulum
**15**	YKL142W	MRP8	MRP8; YKL3; YKL142W Mitochondrial ribosomal protein of the small subunit
**16**	YKR091W	SRL3	SRL3; YKR411; YKR091W Protein of unknown function
**17**	YLR120C	YAP3	YPS1; (YAP3); L9233.9; L2961; YLR120C Yapsin 1, GPI-anchored aspartyl protease that cleaves C-terminal to paired basic residues(Aspergillopepsin I)
**18**	YLR121C		YPS3; (YPS4); L9233.10; L2964; YLR121C GPI-anchored aspartyl protease
**19**	YLR194C		YLR194C; L8167.13 Protein of unknown function
**20**	YLR414C		YLR414C; L9931.8 Protein of unknown function

**21**	YMR008C	PLB1	PLB1; YM8270.10; YMR008C Phospholipase B, preferentially deacylates phosphatidylcholine and phosphatidylethanolamine
**22**	YMR315W		YMR315W; YM9924.07 Protein of unknown function
**23**	YMR316W		DIA1; YM9924.08; YMR316W Protein of unknown function containing a zinc carboxypeptidase motif PS00133; mRNA abundance is reduced bythe drug FK506 in a calcineurin- and immunophilin-dependent manner
**24**	YNL192W	CHS1	CHS1; N1404; YNL192W Chitin synthase I, has a repair function during cell separation
**25**	YNL208W		YNL208W; N1338 Protein of unknown function
**26**	YOL016C	CMK2	CMK2; O2325; YOL016C Calcium/calmodulin-dependent serine/threonine protein kinase (CaM kinase) type II
**27**	YOR036W	PEP12	PEP12; VPL6; VPT13; VPS6; OR26.29; O2750; YOR036W Syntaxin homolog (t-SNARE) involved in Golgi to vacuole transport
**28**	YOR208W	PTP2	PTP2; O4849; YOR208W Protein tyrosine phosphatase (PTPase), involved in Hog1p MAP kinase high-osmolarity sensing pathway and Fus3pMAP kinase pheromone response pathway
**29**	YOR220W		YOR220W; YOR50-10; O5010; O5050 Protein of unknown function
**30**	YOR385W		YOR385W; O6768 Protein of unknown function
**31**	YPL088W		YPL088W; LPG20 Putative aryl alcohol dehydrogenase, may participate in late steps of degradation of aromatic compounds that arise from thedegradation of lignocellulose
**32**	YPR154W		YPR154W; LSB2; P9584.5 Protein with one SH3 domain, has similarity to myosin ID and IC heavy chains, human growth factor receptor-boundgrb2 protein, C. elegans sex muscle abnormal protein 5
**33**	YMR316CA		YMR316C-A Protein of unknown function

**Ty retro-transposons genes**			
**Serial #**	**ORF**	**Name**	**description**
**1**	YAR009C		
**2**	YAR010C		
**3**	YBR012C		YBR012C; YBR0205 Protein of unknown function, has a peroxisomal targeting signal
**4**	YBR013C		YBR013C; YBR0209 Protein of unknown function
**5**	YBR083W	TEC1	TEC1; (ROC1); YBR0750; YBR083W Transcriptional activator, involved with Ste12p in pseudohyphal formation
**6**	YCL019W		
**7**	YCL020W		
**8**	YCL030C	HIS4	HIS4; YCL183; YCL030C Phosphoribosyl-AMP cyclohydrolase / phosphoribosyl-ATP pyrophosphohydrolase / histidinol dehydrogenase,second, third, and tenth steps of histidine biosynthesis pathway
**9**	YDL066W	IDP1	IDP1; D2522; YDL066W Isocitrate dehydrogenase (NADP+), mitochondrial
**10**	YDR366C		YDR366C; D9481.10 Protein of unknown function
**11**	YER138C		
**12**	YER160C		

**13**	YER175C		YER175C; SYGP-ORF63 Protein with predicted S-adenosylmethionine-dependent methyltransferase motif, has similarity to Yhr209p; transcriptioninduced by the drug FK506 in a GCN4-dependent manner
**14**	YFR030W	MET10	MET10; YFR030W Assimilatory sulfite reductase subunit, flavin-binding (alpha) subunit, part of the sulfate assimilation pathway
**15**	YGL117W		YGL117W; G2935 Protein of unknown function transcription induced by the drug FK506 in a GCN4-dependent manner
**16**	YGL260W		YGL260W; NRB76; G0332 Protein with strong similarity to Yir040p and members of the Ybl108p/Ycr103p/Ykl223p family
**17**	YGR156W		YGR156W; G6670 Protein of unknown function
**18**	YHR213W		YHR213W; H9177.5 Protein with similarity to the N-terminus of Flo1p and identical to Yar062p, probable pseudogene
**19**	YIL060W		YIL060W Protein of unknown function
**20**	YIL117C	PRM5	PRM5; YIL117C Protein of unknown function regulated by pheromone
**21**	YJR025C	HAD1	BNA1; HAD1; J1550; YJR025C 3-hydroxyanthranilate 3,4-dioxygenase, involved in biosynthesis of nicotinic acid from tryptophan
**22**	YJR026W		
**23**	YJR027W		
**24**	YJR028W		
**25**	YJR029W		
**26**	YKL218C		SRY1; YKL218C Pyridoxal 5'phosphate-dependent enzyme, has strong similarity to mouse glial serine racemase and E. coli threonine dehydratase
**27**	YLR042C		YLR042C; L1929 Putative GPI-anchored protein of unknown function
**28**	YLR256W	HAP1	HAP1; (CYP1); L9672.1; YLR256W Transcription factor with heme-dependent DNA-binding activity, responsible for heme-dependent activationof many genes
**29**	YLR334C		YLR334C; L8300.11 Protein of unknown function
**30**	YLR343W		YLR343W; L8300.5; GAS2 Protein with strong similarity to Gas1p, member of the GPI-anchored beta(1-3)glucanosyltransferases family
**31**	YML039W		
**32**	YML040W		
**33**	YML045W		
**34**	YMR045C		
**35**	YMR046C		
**36**	YMR050C		
**37**	YMR051C		
**38**	YMR094W	CTF13	CTF13; CBF3C; YM9582.19; YM6543.01; YMR094W Component (subunit c) of Cbf3 kinetochore complex, contains an F-box domain

**39**	YMR095C	SNO1	SNO1; YM6543.02; YMR095C Putative pyridoxine (vitamin B6) biosynthetic enzyme with similarity to glutamine aminotransferases, has strongsimilarity to Sno1p and Sno3p
**40**	YMR096W	SNZ1	SNZ1; YM6543.03; YMR096W Putative pyridoxine (vitamin B6) biosynthetic enzyme, member of the stationary phase-induced gene family whichincludes Snz2p and Snz3p
**41**	YMR166C		YMR166C; YM8520.15 Member of the mitochondrial carrier (MCF) protein family of membrane transporters
**42**	YOL104C	NDJ1	NDJ1; TAM1; HRF352; O0763; YOL104C Meiotic telomere protein, involved in normal chromosomal synapsis and segregation
**43**	YOL106W		YOL106W; O0751 Protein of unknown function
**44**	YPR167C	MET16	MET16; P9325.8; YPR167C 3'-Phosphoadenylylsulfate reductase (PAPS), part of the sulfate assimilation pathway
**45**	YHR214CB		
**46**	YBR012WB		
**47**	YBL101WB		
**48**	YBR012WA		
**49**	YBL005WB		
**50**	YBL005WA		
**51**	YBL101WA		
**52**	YBL107WA		
**53**	YLR035CA		YLR035C-A Protein of unknown function
**54**	YGR122CA		YGR122C-A Protein of unknown function
**55**	YPR002CA		YPR002C-A Protein of unknown function, has similarity to Ylr334p and Yol106p
**56**	YMR046WA		YMR046W-A Protein of unknown function, has similarity to Ylr334p and Yol106p
**57**	YDR170WA		YDR170W-A Protein of unknown function
**58**	YFL002WA		
**59**	YCR018CA		YCR018C-A Protein of unknown function, has similarity to Ylr334p and Yol106p
**60**	YMR158CB		YMR158C-B Protein of unknown function, has similarity to Hap1p and Ydr340p
**61**	YKR035WA		DID2; FTI1; YKR035W-A Class E vacuolar protein-sorting (vps) factor

**Peroxide shock genes**			
**Serial #**	**ORF**	**Name**	**description**
**1**	YBL064C		YBL064C; PRX1; YBL0503; MTP1; mTPx YBL0524 Mitochondrial thiol peroxidase
**2**	YBR008C	FLR1	FLR1; YBR0120; YBR008C Member of the multidrug-resistance 12-spanner (DHA12) family of the major facilitator superfamily (MFS-MDR)
**3**	YBR244W		GPX2; AMI1; YBR1632; YBR244W Glutathione peroxidase, has a role in mitochondrial morphology
**4**	YCR102C		YCR102C Protein with similarity to B subtilis sorbitol dehydrogenase

**5**	YDL243C		AAD4; D0752; YDL243C Putative aryl-alcohol dehydrogenase, may participates in late steps of degradation of aromatic compounds that arise fromthe degradation of lignocellulose
**6**	YDR453C		YDR453C; D9461.38; cTPxII Cytoplasmic thiol peroxidase
**7**	YDR513W	TTR1	TTR1; GRX2; TTR D9719.17; YDR513W Glutaredoxin (thioltransferase, glutathione reductase)
**8**	YDR533C		YDR533C; D9719.36 Protein of unknown function
**9**	YFL056C		AAD6; YFL056C Probable aryl alcohol dehydrogenase, may participate in late steps of degradation of aromatic compounds that arise from thedegradation of lignocellulose
**10**	YFL057C		YFL057C Putative aryl alcohol dehydrogenase, may participate in late steps of degradation of aromatic compounds that arise from the degradationof lignocellulose
**11**	YJR096W		YJR096W; J1926 Protein with similarity to aldolase reductase
**12**	YKL071W		YKL071W Protein with similarity to proteins of the short-chain alcohol dehydrogenase family expression is regulated by Yap1p transcription factor
**13**	YKL103C	LAP4	LAP4; APE1; API YSC1; YKL455; YKL103C Aminopeptidase I (yscI, API) of the vacuole
**14**	YKR076W	ECM4	ECM4; YKR076W Protein possibly involved in cell wall structure or biosynthesis
**15**	YLL060C		GTT2; L0560; YLL060C Glutathione transferase
**16**	YLR108C		YLR108C; L9354.6; L2913 Protein of unknown function
**17**	YLR460C		YLR460C; L9122.7 Protein with strong similarity to Ycr102p
**18**	YML131W		YML131W; YM4987.04 Putative NAD-dependent oxidoreductase
**19**	YMR090W		YMR090W; YM9582.15 Protein with similarity to malate dehydrogenases
**20**	YMR173W	DDR48	DDR48; FSP YM8010.03; YMR173W Stress protein induced by heat shock, DNA damage, or osmotic stress
**21**	YNL134C		YNL134C; N1214; N1847 Protein with similarity to C. carbonum toxD gene
**22**	YNL331C		AAD14; N0300; YNL331C Putative aryl alcohol dehydrogenase, may participate in late steps of degradation of aromatic compounds that arise fromthe degradation of lignocellulose
**23**	YOL150C		YOL150C; AOE103; O0448 Protein of unknown function questionable ORF
**24**	YOL151W	GRE2	GRE2; AOA342; O0446; YOL151W Protein involved in diamide tolerance and induced by osmotic stress, member of a family (GRE2, YGL039W,YGL157W, YDR541C) with similarity to plant dihydroflavonol-4-reductases
**25**	YOL165C		AAD15; O0205; YOL165C Putative aryl alcohol dehydrogenase, may participate in late steps of degradation of aromatic compounds that arisefrom the degradation of lignocellulose

**TCA cycle genes**			
**Serial #**	**ORF**	**Name**	**description**
**1**	YCR005C	CIT2	CIT2; YCR043; YCR005C Citrate synthase, peroxisomal (nonmitochondrial), converts acetyl-CoA and oxaloacetate into citrate plus CoA
**2**	YLR304C	ACO1	ACO1; GLU1; L8003.22; YLR304C Aconitate hydratase (aconitase), converts citrate to cis-aconitate

**3**	YNL037C	IDH1	IDH1; N2690; YNL037C Isocitrate dehydrogenase (NAD+) subunit 1, mitochondrial, required for oxidative function of the tricarboxylic acid cycle
**4**	YNR001C	CIT1	CIT1; GLU3; N2019; YNR001C Citrate synthase, mitochondrial, converts acetyl-CoA and oxaloacetate into citrate plus CoA
**5**	YOR135C		YOR135C; O3323 Protein of unknown function
**6**	YOR136W	IDH2	IDH2; O3326; YOR3326; YOR136W Isocitrate dehydrogenase (NAD+) subunit 2, mitochondrial, required for oxidative function of thetricarboxylic acid cycle
**7**	YPL087W		YDC1; LPG21; YPL087W Alkaline ceramidase with specificity for dihydroceramide
**8**	YPL135W		ISU1; NUA1; LPI10; YPL135W Protein with similarity to iron-sulfur cluster nitrogen fixation proteins
**9**	YPL154C	PEP4	PEP4; PRA1; PHO9; P2585; YPL154C Proteinase A (PrA/yscA/saccharopepsin) aspartyl protease required for activation of various degradativeenzymes
**10**	YPR002W		PDH1; YP9723.02; LPZ2W; YPR002W Protein possibly involved in propionate utilization

**Gluconeogenesis genes**			
**Serial #**	**ORF**	**Name**	**description**
**1**	YCR010C		SPG2; YCR010C Protein of unknown function, has strong similarity to Ydr384p and Ynr002p
**2**	YER065C	ICL1	ICL1; YER065C Isocitrate lyase, carries out part of the glyoxylate cycle, required for gluconeogenesis
**3**	YFL030W		YFL030W Putative alanine glyoxylate aminotransferase (serine pyruvate aminotransferase)
**4**	YGR067C		YGR067C; G4548 Protein of unknown function, has two tandem zinc finger domains
**5**	YGR236C		SPG1; ORF129; YGR236C; G8578 Protein of unknown function, induced during aerobic growth
**6**	YGR243W		YGR243W; G8620 Protein of unknown function
**7**	YHR096C	HXT5	HXT5; YHR096C Member of the hexose transporter family of the major facilitator superfamily (MFS)
**8**	YIL057C		YIL057C Protein of unknown function
**9**	YIL136W	OM45	OM45; YIL136W Protein of the outer mitochondrial membrane
**10**	YKL026C		GPX1; YKL026C Glutathione peroxidase
**11**	YKL093W	MBR1	MBR1; YKL425; YKL440; YKL093W Protein involved in mitochondrial biogenesis
**12**	YKL187C		YKL187C Protein with similarity to 4-mycarosyl isovaleryl-CoA transferase
**13**	YKL217W	JEN1	JEN1; YKL217W Pyruvate and lactate-proton symporter, member of the major facilitator superfamily (MFS)
**14**	YKR097W	PCK1	PCK1; PPC1; JPM2; PEPC YKR097W Phosphoenolpyruvate carboxykinase (ATP), rate limiting gluconeogenic enzyme
**15**	YLR174W	IDP2	IDP2; L9470.12; YLR174W Isocitrate dehydrogenase (NADP+), cytosolic
**16**	YLR377C	FBP1	FBP1; L8039.18; YLR377C Fructose-1,6-bisphosphatase, gluconeogenic enzyme, activity is inhibited by protein kinase A-mediatedphosphorylation
**17**	YML054C	CYB2	CYB2; YM9958.08; YML054C Cytochrome b2 [L-(+)-lactate cytochrome c oxidoreductase] converts L-lactate to pyruvate

**18**	YMR081C	ISF1	ISF1; MBR3; YM9582.06; YMR081C Protein that participates with Nam7p/Upf1p in suppression of mitochondrial splicing defect
**19**	YMR107W		YMR107W; YM9718.06 Protein of unknown function, induced during aerobic growth
**20**	YMR206W		YMR206W; YM8325.07 Protein of unknown function
**21**	YNL117W	MLS1	MLS1; N1921; YNL117; YNL117W Malate synthase 1, functions in glyoxylate cycle, has near identity to Dal7p
**22**	YNL194C		YNL194C; N1394 Protein with similarity to Sur7p
**23**	YNL195C		YNL195C; N1390 Protein of unknown function, induced during aerobic growth
**24**	YNR002C	FUN34	YNR002C; N2029; (FUN34) Protein of unknown function, has strong similarity to Ycr010p and Ydr384p
**25**	YOR178C	GAC1	GAC1; O4625; YOR178C Regulatory subunit for protein serine/threonine phosphatase Glc7p
**26**	YPL054W	LEE1	LEE1; LPE18; YPL054W Protein of unknown function
**27**	YPL147W	PXA1	PXA1; PAL1; SSH2; LPI1; PAT2; P2607; YPL147W Protein required for long-chain fatty acid transport across the peroxisomal membrane,member of the ATP-binding cassette (ABC) superfamily, has similarity to a human gene associated with adrenoleukodystrophy
**28**	YPL186C		YPL186C; P2213 Protein of unknown function
**29**	YPL230W		USV1; P1421; YPL230W Putative finger transcripton factor, has a two tandem C2H2-type zinc fingers
**30**	YPR030W		CSR2; MRG19; YP9367.10; YPR030W Protein of unknown function
