# Supplementary material for: Strategy of Transcription Regulation in the Budding Yeast
Source: PLoS One. 2007 Feb 28;2(2):e250. doi: 10.1371/journal.pone.0000250 (PMC1803021; doi:10.1371/journal.pone.0000250)
Supplement: Table S2 — Correlations between growth and the modules average expression (0.07 MB PDF) [file pone.0000250.s005.pdf]

Gene Group	No. of genes	Continuous culture		Compendium data	
		Correlation	P-value	Correlation	P-value
Cell-cycle G2/M	56	0.33	0.03	**0.54**	**$2{\cdot}10^{-16}$**
Cell-cycle M/G1	63	0.34	0.03	0.17	0.01
Cell-cycle G1	280	0.09	0.32	**0.58**	**$6{\cdot}10^{-19}$**
Histones	43	0.11	0.28	**0.50**	**$8{\cdot}10^{-14}$**
Ribosomal proteins	117	0.21	0.12	**0.53**	**$2{\cdot}10^{-15}$**
Mitochondrial ribosomal proteins	100	0.26	0.07	-0.06	0.44
Phospholipids metabolism	22	0.30	0.05	**0.45**	**$5{\cdot}10^{-11}$**
Oxidative phosphorylation	51	0.29	0.06	-0.03	0.68
Cell wall	24	0.11	0.27	**0.43**	**$3{\cdot}10^{-10}$**
**Ribosomal biogenesis**	**231**	**0.09**	**0.33**	**0.03**	**0.67**
PAU	64	-0.42	0.02	**-0.49**	**$4{\cdot}10^{-13}$**
Calcium calmodulin	33	-0.38	0.03	**-0.37**	**10^{-7}**
Amino-acid biosynthesis	303	0.03	0.43	**-0.41**	**$3{\cdot}10^{-9}$**
Iron transport	54	-0.12	0.29	**-0.47**	**$2{\cdot}10^{-12}$**
Phosphate + iron utilization	42	0.06	0.37	**-0.29**	**$4{\cdot}10^{-5}$**
TCA cycle	10	0.15	0.21	**-0.44**	**10^{-10}**
Ty retro-transposons	61	0.20	0.13	**-0.39**	**$2{\cdot}10^{-8}$**
Peroxide shock	25	-0.19	0.18	**-0.45**	**$6{\cdot}10^{-11}$**
Stress	255	-0.16	0.23	-0.19	$8{\cdot}10^{-3}$
Gluconeogenesis	30	0.08	0.33	**-0.31**	**10^{-5}**

Table S2. Correlations between growth and the modules average expression. The table summarizes the analysis which spanned over hundreds of gene modules. For each gene group and experiment we calculated the correlation between the cells growth rate and the group average expression. Significant (P-value $< 10^{-3}$) positive (blue) and negative (red) correlations were found only in the compendium data. Notably, in both experiments the correlation between growth rate and ribosomal biogenesis genes is very low.
