# Supplementary material for: Strategy of Transcription Regulation in the Budding Yeast
Source: PLoS One. 2007 Feb 28;2(2):e250. doi: 10.1371/journal.pone.0000250 (PMC1803021; doi:10.1371/journal.pone.0000250)
Supplement: Table S3 — Effect of medium limitations on the enrichment of positive and negative correlated genes with gene modules. (0.09 MB PDF) [file pone.0000250.s006.pdf]

Gene group	No. of genes	Histidine depletion		Glucose depletion		Both	
		Enrichment [%]	P-value	Enrichment [%]	P-value	Enrichment [%]	P-value
Cell-cycle G2-M	56	14	$6 \cdot 10^{-3}$	13	0.02	**25**	$\mathbf{4 \cdot 10^{-7}}$
Cell-cycle M-G1	63	**40**	$\mathbf{< 10^{-15}}$	**17**	$\mathbf{2 \cdot 10^{-4}}$	**37**	$\mathbf{< 10^{-15}}$
Cell-cycle G1	280	7	0.11	3	0.97	5	0.65
Histones	43	16	$5 \cdot 10^{-3}$	2	0.89	12	0.06
Ribosomal proteins	117	1	1.00	1	1.00	1	1.00
Mitochondrial ribosomal proteins	100	9	0.06	**30**	$\mathbf{8 \cdot 10^{-13}}$	**26**	$\mathbf{2 \cdot 10^{-12}}$
Phospholipids metabolism	22	0	1	**27**	$\mathbf{6 \cdot 10^{-4}}$	23	$4 \cdot 10^{-3}$
Oxidative phosphorylation	51	12	0.04	**25**	$\mathbf{8 \cdot 10^{-7}}$	**29**	$\mathbf{10^{-8}}$
Cell wall	24	**25**	$\mathbf{9 \cdot 10^{-4}}$	0	1	4	0.71
**Ribosomal biogenesis**	**231**	**6**	**0.26**	**1**	**1.00**	**0**	**1.00**
PAU	64	**22**	$\mathbf{2 \cdot 10^{-6}}$	**23**	$\mathbf{4 \cdot 10^{-7}}$	**19**	$\mathbf{6 \cdot 10^{-5}}$
Calcium calmodulin	33	**30**	$\mathbf{3 \cdot 10^{-6}}$	18	$5 \cdot 10^{-3}$	**30**	$\mathbf{3 \cdot 10^{-6}}$
Amino-acid biosynthesis	303	6	0.34	8	0.03	5	0.66
Iron transport	54	9	0.13	6	0.51	0	1
Phosphate + iron utilization	42	12	0.06	12	0.06	0	1
TCA cycle	10	10	0.40	30	0.01	10	0.40
Ty retro-transposons	61	10	0.08	3	0.82	5	0.59
Peroxide shock	25	4	0.72	20	$7 \cdot 10^{-3}$	20	$7 \cdot 10^{-3}$
Stress	255	**14**	$\mathbf{9 \cdot 10^{-9}}$	**11**	$\mathbf{2 \cdot 10^{-4}}$	4	0.90
Gluconeogenesis	30	0	1	**27**	$\mathbf{8 \cdot 10^{-5}}$	10	0.19

Table S3. Effect of medium limitations on the enrichment of positive and negative correlated genes with previously defined gene modules. For each gene group and experiment we calculated the enrichment with the positive (blue) and negative (red) correlated genes. Notably, most of the observed scores are specific to a particular experimental condition (histidine-limited vs. glucose-limited chemostat). Note that none of the gene modules were enriched with both positive and negative correlated genes in the same experiment, and only the highest score is displayed. Less significant enrichments are shown in black (P-value > 10^{-3}).
