# Supplementary material for: Strategy of Transcription Regulation in the Budding Yeast
Source: PLoS One. 2007 Feb 28;2(2):e250. doi: 10.1371/journal.pone.0000250 (PMC1803021; doi:10.1371/journal.pone.0000250)
Supplement: Text S1 — Supplementary Methods (0.02 MB PDF) [file pone.0000250.s007.pdf]

Supplementary Methods

RNA extraction, microarray hybridization, scanning and quantification

Total RNA was extracted from sampled frozen cells samples using yeast RNA purification kit (Epicenter) and subjected to reverse-transcription reaction using M-MLV Reverse Transcriptase RNase H Minus (Promega). Arrays were purchased from the Microarray Centre, University Health Network, Ontario, where PCR products are printed.

For each hybridization, cDNA samples were labeled with Cy3 and Cy5 and combined with blockers: 5µg Herring sperm (Promega), 5µg tRNA (Gibco) and 17.5µg Poly A (Poly A oligos were synthesized with mixed length of 40, 50 and 60 Adenine residues). The labeled cDNAs were concentrated to 40µl using Microcon (Millipore) and 40µl of hybridization x2 solution (10x SSC, 50% formamide and 0.2% SDS) was added. Microarrays containing all yeast ORFs were pre-hybridized by incubating at 42ºC for 45 minutes in a solution containing 1% BSA, 25% formamide, 5x SSC and 0.1% SDS. The slides were washed in sterile water and dried by centrifugation (3 minutes, 2000 rpm). The labeled samples were boiled for 5 minutes, centrifuged for 1 minute, hybridized on the slide and placed in a hybridization chamber (Corning) for overnight incubation at 42ºC. The slides were then washed for 5 minutes at 42ºC with a solution containing 2x SSC and 0.1% SDS. Additional wash was performed at room temperature with a solution containing 0.1x SSC and 0.1% SDS, followed by three additional washes at room temperature in 0.1x SSC solution.

Images were obtained either using ScanArray 4000 scanner (Packard BioScience) or Agilent's DNA microarray scanner. Image analysis was performed using QuantArray version 3 software (Perkin-Elmer Life Sciences, Boston, MA) or SpotReader (Niles Scientific). Note that microarrays during each experiment were analyzed with the same scanner and image analysis software.

Calculation of dynamic range

The dynamic range of a gene indicates the extent for which the gene expression average level is affected by the environmental perturbations. The dynamic range (R) of a gene in a given perturbation (i) was defined as the difference between the average two highest expression levels (E_1, E_2) and the average two lowest expression levels (E_{n-1}, E_n): $R_i = \left(\frac{E_1 + E_2}{2}\right) - \left(\frac{E_{n-1} + E_n}{2}\right)$. The dynamic range of a gene was defined as the average dynamic range over all perturbations: $R = \sum_{i=1}^{10} R_i$.

In order to reduce the noise in our correlation analysis we ignored genes with low dynamic range. Specifically, we set the threshold on 2/3 of the median genes dynamic range. Genes for which the dynamic range was lower than this threshold were ignored (~15% of the genes).
